# Supplementary material for: Computational Analysis of S1PR1 SNPs Reveals Drug Binding Modes Relevant to Multiple Sclerosis Treatment
Source: Pharmaceutics. 2024 Nov 3;16(11):1413. doi: 10.3390/pharmaceutics16111413 (PMC11597183; doi:10.3390/pharmaceutics16111413)
Supplement: Supplementary file 1 [file pharmaceutics-16-01413-s001.zip › pharmaceutics-3262166-supplementary.pdf]

Supplementary Materials

for

Computational Analysis of S1PR1 SNPs Reveals Drug Binding Modes Relevant to Multiple Sclerosis Treatment

Katarina Kores [1], Samo Lešnik [1,2] and Urban Bren [1,2,3,*]

[1] Laboratory of Physical Chemistry and Chemical Thermodynamics, Faculty of Chemistry and Chemical Engineering, University of Maribor, Smetanova 17, SI-2000 Maribor, Slovenia; katarina.kores@um.si (K.K.); samo.lesnik@um.si (S.L.)
[2] Institute of Environmental Protection and Sensors, IOS, Beloruska 7, SI-2000 Maribor, Slovenia
[3] Faculty of Mathematics, Natural Sciences and Information Technologies, University of Primorska, Glagoljaška 8, SI-6000 Koper, Slovenia
* Correspondence: urban.bren@um.si (U.B.)

Table S1 – Computed results for method validation with S1P bound to mutated S1PR1 for all the parallels.

System	Parallel	E^{vdW}	E^{elec}	ΔG_{bind}	System	Parallel	E^{vdW}	E^{elec}	ΔG_{bind}	System	Parallel	E^{vdW}	E^{elec}	ΔG_{bind}
water	1	-16.87	-332.72	/	E121$^{3.29}$A	1	-32.18	-374.04	-9.37	W269$^{6.48}$E	1	-28.34	-458.59	-15.22
	2	-16.72	-343.68	/		2	-32.74	-372.27	-9.47		2	-30.60	-402.90	-11.25
	3	-16.65	-351.93	/		3	-33.57	-383.60	-10.87		3	-28.25	-461.06	-15.40
	4	-16.83	-334.69	/		4	-30.70	-470.46	-17.37		4	-31.66	-412.39	-12.59
	5	-18.24	-372.77	/		5	-33.19	-384.81	-10.81		5	-30.38	-460.41	-16.32
WT	1	-34.36	-412.61	-13.85	E121$^{3.29}$Q	1	-32.56	-387.02	-10.72	R292$^{7.34}$A	1	-33.80	-414.91	-13.80
	2	-31.51	-401.35	-11.52		2	-29.96	-420.37	-12.52		2	-32.44	-389.15	-10.85
	3	-35.87	-401.89	-13.58		3	-32.66	-393.46	-11.34		3	-32.56	-415.77	-13.31
	4	-35.37	-415.71	-14.59		4	-33.46	-365.77	-9.22		4	-30.54	-431.58	-13.80
	5	-33.55	-432.57	-15.27		5	-28.88	-479.82	-17.37		5	-28.37	-463.26	-15.65
N101$^{2.60}$I	1	-32.51	-404.11	-12.23	W269$^{6.48}$A	1	-28.65	-421.97	-12.06	R292$^{7.34}$V	1	-33.98	-434.98	-15.69
	2	-29.19	-413.55	-11.55		2	-31.07	-397.86	-11.01		2	-31.41	-417.29	-12.91
	3	-32.11	-382.85	-10.14		3	-31.40	-409.24	-12.18		3	-33.28	-402.53	-12.44
	4	-31.35	-401.65	-11.48		4	-30.34	-398.03	-10.69		4	-35.49	-414.70	-14.55
	5	-37.49	-410.88	-15.13		5	-33.10	-375.83	-9.96		5	-31.31	-391.70	-10.56
N101$^{2.60}$K	1	-34.09	-416.43	-14.07										
	2	-31.80	-416.67	-13.04										
	3	-32.13	-415.02	-13.04										
	4	-28.95	-453.18	-15.01										
	5	-33.16	-407.05	-12.80										

Table S2 – Calculated binding free energies of S1P and investigated drugs to the S1PR1 binding site in combination with different SNPs for all the parallels.

System	Parallel	S1P			fingolimod			siponimod			ozanimod			ponesimod		
		E^{vdW}	E^{elec}	$\Delta G_{\mathbf{bind}}$	E^{vdW}	E^{elec}	$\Delta G_{\mathbf{bind}}$	E^{vdW}	E^{elec}	$\Delta G_{\mathbf{bind}}$	E^{vdW}	E^{elec}	$\Delta G_{\mathbf{bind}}$	E^{vdW}	E^{elec}	$\Delta G_{\mathbf{bind}}$
	1	-34.20	-406.31	-13.99	-29.69	-390.03	-10.41	-57.96	-195.56	-15.98	-54.57	-133.80	-13.08	-63.60	-26.11	-13.44
	2	-29.60	-428.61	-13.29	-31.50	-401.43	-12.30	-58.76	-202.33	-16.96	-55.69	-122.71	-12.80	-62.72	-26.17	-12.97
M124$^{3.32}$T	3	-34.62	-412.77	-14.73	-29.38	-402.65	-11.26	-59.80	-183.51	-16.02	-55.58	-117.94	-12.36	-64.41	-26.79	-13.93
	4	-32.75	-414.10	-13.83	-31.02	-393.39	-11.40	-57.84	-193.30	-15.74	-56.36	-116.62	-12.68	-61.51	-32.05	-12.78
	5	-31.73	-397.62	-11.96	-31.57	-385.45	-11.06	-55.68	-201.95	-15.26	-56.46	-116.08	-12.69	-61.38	-33.04	-12.79
	1	-29.82	-407.33	-11.70	-30.60	-402.93	-11.94	-59.32	-202.96	-17.31	-56.01	-117.60	-12.56	-63.04	-21.64	-12.78
	2	-34.64	-397.17	-13.49	-32.45	-374.59	-10.67	-56.62	-190.84	-14.88	-58.57	-113.08	-13.58	-62.94	-31.39	-13.50
V132$^{3.40}$M	3	-33.66	-386.99	-12.15	-32.47	-377.43	-10.90	-59.37	-194.97	-16.70	-55.98	-115.41	-12.37	-63.78	-23.76	-13.35
	4	-33.63	-399.34	-13.12	-29.16	-389.86	-10.11	-58.19	-199.56	-16.43	-57.01	-117.44	-13.09	-64.46	-24.12	-13.74
	5	-31.34	-421.02	-13.62	-30.98	-390.41	-11.14	-56.36	-197.73	-15.29	-56.56	-109.42	-12.21	-64.78	-24.94	-13.98
	1	-31.32	-408.99	-12.65	-28.84	-383.67	-9.44	-58.33	-202.29	-16.72	-56.11	-130.60	-13.66	-64.27	-27.83	-13.94
	2	-32.18	-389.71	-11.57	-29.59	-391.52	-10.48	-58.38	-197.53	-16.37	-56.08	-116.80	-12.54	-65.16	-29.88	-14.58
F205$^{5.42}$L	3	-29.18	-452.51	-14.97	-31.90	-388.38	-11.48	-57.04	-203.30	-16.11	-55.67	-115.43	-12.21	-64.74	-30.46	-14.40
	4	-30.87	-443.57	-15.17	-30.86	-408.36	-12.51	-59.18	-201.04	-17.08	-56.30	-117.63	-12.73	-64.65	-30.00	-14.31
	5	-34.14	-396.76	-13.19	-31.47	-375.92	-10.24	-58.98	-205.13	-17.30	-55.01	-113.03	-11.66	-61.27	-27.13	-12.26
	1	-34.52	-400.25	-13.68	-30.54	-399.52	-11.63	-57.55	-201.19	-16.21	-53.48	-116.11	-11.08	-63.67	-36.34	-14.29
	2	-30.47	-416.28	-12.77	-30.37	-389.17	-10.71	-58.07	-199.87	-16.39	-55.16	-115.45	-11.94	-65.08	-21.55	-13.87
T207$^{5.44}$I	3	-31.94	-427.52	-14.46	-34.52	-384.11	-12.55	-60.00	-200.88	-17.51	-53.71	-131.56	-12.44	-65.38	-26.78	-14.45
	4	-33.56	-406.29	-13.64	-32.98	-386.48	-11.90	-58.69	-196.55	-16.46	-55.66	-115.67	-12.22	-64.87	-20.43	-13.67
	5	-30.09	-408.13	-11.91	-32.08	-368.98	-10.02	-59.65	-186.43	-16.17	-54.25	-116.79	-11.55	-64.18	-29.27	-14.00
	1	-29.73	-433.44	-13.75	-30.80	-383.38	-10.48	-57.65	-201.82	-16.32	-56.69	-117.54	-12.93	-62.14	-26.72	-12.70
	2	-29.16	-432.59	-13.37	-29.69	-411.12	-12.10	-56.81	-195.41	-15.35	-58.11	-114.13	-13.42	-63.23	-20.13	-12.76
T211$^{5.48}$P	3	-32.86	-405.48	-13.20	-30.87	-398.87	-11.76	-59.18	-189.68	-16.17	-54.85	-116.61	-11.86	-63.45	-23.09	-13.11
	4	-32.94	-396.98	-12.56	-29.29	-419.69	-12.57	-56.73	-192.14	-15.05	-55.22	-115.56	-11.97	-63.77	-22.26	-13.22
	5	-33.77	-400.77	-13.31	-29.10	-420.08	-12.50	-59.12	-200.68	-17.02	-54.59	-115.98	-11.67	-64.68	-23.82	-13.84
A293$^{7.35}$T	1	-31.16	-403.98	-12.16	-31.85	-386.08	-11.26	-57.85	-202.01	-16.44	-53.86	-123.18	-11.85	-66.57	-25.10	-14.96

	2	-30.14	-406.41	-11.80	-32.54	-374.17	-10.68	-56.11	-198.41	-15.21	-55.85	-114.56	-12.23	-63.03	-29.62	-13.41
	3	-34.30	-395.97	-13.21	-33.31	-392.43	-12.56	-60.01	-198.35	-17.31	-55.12	-116.48	-12.00	-64.64	-28.29	-14.17
	4	-34.56	-410.12	-14.48	-30.26	-408.22	-12.17	-59.88	-198.50	-17.26	-53.68	-136.12	-12.79	-65.17	-21.72	-13.93
	5	-32.25	-379.80	-10.81	-32.16	-401.73	-12.68	-59.77	-200.03	-17.32	-55.99	-119.49	-12.70	-64.82	-24.61	-13.98
$A293^{7.35}V$	1	-31.78	-400.79	-12.24	-29.24	-400.63	-11.02	-58.16	-206.24	-16.95	-57.64	-119.28	-13.58	-65.73	-24.22	-14.44
	2	-31.31	-398.64	-11.81	-33.52	-401.13	-13.37	-59.03	-193.60	-16.40	-54.96	-117.04	-11.95	-63.35	-27.19	-13.39
	3	-33.36	-394.82	-12.61	-31.00	-388.84	-11.02	-57.88	-198.37	-16.17	-55.95	-113.02	-12.16	-64.62	-26.69	-14.03
	4	-34.53	-402.74	-13.88	-30.33	-396.60	-11.28	-57.60	-199.18	-16.08	-55.72	-115.38	-12.23	-65.17	-27.79	-14.42
	5	-31.29	-411.35	-12.82	-32.91	-386.74	-11.89	-58.83	-202.72	-17.03	-55.26	-114.96	-11.95	-66.00	-26.38	-14.76

Table S3 – A detailed interaction analysis for all the systems with bound S1P using PLIP. For better clarity of the obtained results, we focused on the interactions with ≥ 25.0% occupancy throughout each MD simulation. The ligand-atom numbering for S1P is presented in Figure S54.

System	Hydrogen bonds				Hydrophobic interactions				Salt bridges			
	Atom (ligand)	AA	Atom (AA)	Occupancy	Atom (ligand)	AA	Atom (AA)	Occupancy	Atom (ligand)	AA	Atom (AA)	Occupancy
WT	O3	Y29	OH	74.7%	C7	V194	CG1	25.2%	[P,O1,O3,O4,O5]	K34	NZ	79.7%
	O4	Y29	OH	54.7%	C18	L213	CD1	26.1%	[P,O1,O3,O4,O5]	K46	NZ	37.7%
	O5	Y29	OH	38.4%	C6	A293	CB	35.0%	[P,O1,O3,O4,O5]	R120	[NE,NH1,NH2]	95.9%
	O2	N101	OD1	31.7%	C4	L297	CD2	25.4%				
	O5	S105	OG	37.6%								
	O3	G106	N	25.0%								
	O3	T109	OG1	41.3%								
	O5	T109	OG1	25.3%								
	N	R120	NH2	44.3%								
	N	E121	OE2	44.8%								
	N	E121	OE1	37.4%								
	O2	E121	OE1	26.3%								
	O2	E121	OE2	25.7%								
M124$^{3.32}$T	O4	Y29	OH	40.4%					[P,O1,O3,O4,O5]	K34	NZ	79.2%
	O3	Y29	OH	63.6%					[P,O1,O3,O4,O5]	K41	NZ	79.2%
	O5	Y29	OH	60.5%					[P,O1,O3,O4,O5]	R120	[NE,NH1,NH2]	74.7%
	N	N101	ND2	29.1%								
	N	N101	OD1	25.1%								
	O4	S105	OG	43.1%								
	N	R120	NH1	41.3%								
	N	E121	OE2	38.3%								
	N	E121	OE1	29.6%								
	O2	E121	OE1	27.6%								
V132$^{3.40}$M	O4	Y29	OH	28.3%	C18	V209	CG1	25.9%	[P,O1,O3,O4,O5]	K34	NZ	84.9%
	O5	Y29	OH	29.8%					[P,O1,O3,O4,O5]	R120	[NE,NH1,NH2]	87.5%
	O3	Y29	OH	64.3%								
	N	R120	NH2	37.5%								
	N	E121	OE2	51.4%								
	N	E121	OE1	31.3%								
F205$^{5.42}$L	O4	Y29	OH	54.9%	C18	V209	CG1	34.9%	[P,O1,O3,O4,O5]	K34	NZ	91.3%

	O3	Y29	OH	38.9%					[P,O1,O3,O4,O5]	R120	[NE,NH1,NH2]	79.5%
	O7	Y29	OH	28.9%								
	N	N101	OD1	29.7%								
	O5	S105	OG	41.8%								
	N	R120	NH1	26.3%								
	N	E121	OE1	27.3%								
	N	E121	OE2	36.9%								
	02	E121	OE1	29.2%								
	O4	Y29	OH	66.1%	C18	V209	CG1	26.0%	[P,O1,O3,O4,O5]	K34	NZ	83.6%
	O5	Y29	OH	52.4%	C4	L297	CD2	25.5%	[P,O1,O3,O4,O5]	K46	NZ	32.1%
	O2	N101	OD1	31.8%					[P,O1,O3,O4,O5]	R120	[NE,NH1,NH2]	72.4%
T207$^{5.44}$I	O1	N101	ND2	25.7%								
	O3	Y110	OH	27.9%								
	N	R120	NH2	42.7%								
	N	E121	OE2	41.3%								
	N	E121	OE1	40.7%								
	O5	Y29	OH	93.2%					[P,O1,O3,O4,O5]	K34	NZ	90.8%
	O4	Y29	OH	50.2%					[P,O1,O3,O4,O5]	R120	[NE,NH1,NH2]	91.5%
	O3	Y29	OH	56.5%								
T211$^{5.48}$P	O5	T109	OG1	35.2%								
	N	R120	NH2	52.1%								
	N	E121	OE1	36.1%								
	N	E121	OE2	51.4%								
	O5	Y29	OH	42.1%					[P,O1,O3,O4,O5]	K34	NZ	82.1%
	O3	Y29	OH	53.5%					[P,O1,O3,O4,O5]	R120	[NE,NH1,NH2]	68.6%
	O4	Y29	OH	76.6%								
A293$^{7.35}$T	N	R120	NH2	37.7%								
	N	E121	OE2	40.2%								
	N	E121	OE1	38.9%								
	O2	E121	OE2	35.4%								
	O4	Y29	OH	31.3%	C18	V209	CG1	26.5%	[P,O1,O3,O4,O5]	K34	NZ	74.3%
	O5	Y29	OH	65.2%					[P,O1,O3,O4,O5]	K46	NZ	29.9%
A293$^{7.35}$V	O3	S105	OG	26.5%					[P,O1,O3,O4,O5]	R120	[NE,NH1,NH2]	72.9%
	O5	S105	OG	27.6%								
	N	R120	NH1	25.4%								

	N	R120	NH2	38.3%
	N	E121	OE1	41.5%
	N	E121	OE2	31.7%

Table S4 – A detailed interaction analysis for all the systems with bound fingolimod using PLIP. For better clarity of the obtained results, we focused on the interactions with ≥ 25.0% occupancy throughout each MD simulation. The ligand-atom numbering for fingolimod phosphate is presented in Figure S55.

System	Hydrogen bonds				Hydrophobic interactions				Salt bridges			
	**Atom (ligand)**	**AA**	**Atom (AA)**	**Occupancy**	**Atom (ligand)**	**AA**	**Atom (AA)**	**Occupancy**	**Atom (ligand)**	**AA**	**Atom (AA)**	**Occupancy**
WT	N	R120	NH2	35.5%					[P,O2,O3,O4,O5]	K34	NZ	77.2%
	N	E121	OE1	36.9%					[P,O2,O3,O4,O5]	R120	[NE,NH1,NH2]	46.1%
	N	E121	OE2	40.9%								
M124$^{3.32}$T	O3	Y29	OH	40.5%					[P,O2,O3,O4,O5]	K34	NZ	70.8%
	N	N101	ND2	31.4%					[P,O2,O3,O4,O5]	R120	[NE,NH1,NH2]	67.1%
	O5	S105	OG	25.7%								
	O3	S105	OG	32.9%								
	N	R120	NH2	48.7%								
	N	E121	OE1	38.6%								
	N	E121	OE2	33.3%								
V132$^{3.40}$M	N	N101	OD1	32.0%	C15	L195	CD2	38.0%	[P,O2,O3,O4,O5]	K34	NZ	68.7%
	O5	S105	OG	43.3%					[P,O2,O3,O4,O5]	R120	[NE,NH1,NH2]	63.3%
	N	R120	NH2	28.4%								
	N	R120	NH1	27.4%								
	N	E121	OE2	38.2%								
	N	E121	OE1	25.1%								
F205$^{5.42}$L	O4	Y29	OH	25.2%	C15	L195	CD2	26.5%	[P,O2,O3,O4,O5]	K34	NZ	69.8%
	N	N101	OD1	37.5%					[P,O2,O3,O4,O5]	K46	NZ	38.0%
	O5	S105	OG	56.4%					[P,O2,O3,O4,O5]	R120	[NE,NH1,NH2]	38.2%
	O3	S105	OG	36.1%								
	N	R120	NH2	26.1%								
T207$^{5.44}$I	N	N101	OD1	61.2%	C15	L195	CD2	30.4%	[P,O2,O3,O4,O5]	K34	NZ	69.7%
	O1	N101	ND2	31.7%					[P,O2,O3,O4,O5]	K41	NZ	38.4%
	O4	S105	OG	63.3%					[P,O2,O3,O4,O5]	R120	[NE,NH1,NH2]	47.7%
	O5	S105	OG	40.1%								
	O1	R120	NH2	34.6%								
	N	R120	NH2	33.6%								
	O1	E121	OE2	30.4%								
T211$^{5.48}$P	N	N101	OD1	36.9%	C15	L195	CD2	29.7%	[P,O2,O3,O4,O5]	K34	NZ	67.9%
	O4	S105	OG	26.1%					[P,O2,O3,O4,O5]	K41	NZ	96.6%

	O1	R120	NH2	32.0%					[P,O2,O3,O4,O5]	R120	[NE,NH1,NH2]	34.3%
	O1	E121	OE2	27.5%								
	O1	E121	OE1	32.9%								
	N	E121	OE2	31.0%								
	O1	V194	O	36.8%								
A293$^{7.35}$T	N	N101	OD1	53.0%	C15	L195	CD2	29.2%	[P,O2,O3,O4,O5]	K34	NZ	79.3%
	N	N101	ND2	44.3%					[P,O2,O3,O4,O5]	R120	[NE,NH1,NH2]	43.0%
	O5	S105	OG	66.3%								
	N	R120	NH2	25.8%								
	N	R120	NH1	29.6%								
	O1	E121	OE1	29.4%								
A293$^{7.35}$V	N	N101	OD1	61.9%	CD2	L195	CD2	25.4%	[P,O2,O3,O4,O5]	K34	NZ	52.6%
	N	N101	ND2	33.2%					[P,O2,O3,O4,O5]	R120	[NE,NH1,NH2]	37.9%
	O5	S105	OG	52.5%								
	O3	S105	OG	57.9%								
	O4	S105	OG	45.6%								
	O1	R120	NH2	35.1%								
	N	R120	NH2	26.7%								
	O1	E121	OE1	37.1%								
	O1	V194	O	38.8%								

Table S5 – A detailed interaction analysis for all systems with bound siponimod using PLIP. For better clarity of the obtained results, we focused on the interactions with ≥ 25.0% occupancy throughout each MD simulation. The ligand-atom numbering for siponimod is presented in Figure S56.

System	Hydrogen bonds				Hydrophobic interactions				Salt bridges			
	Atom (ligand)	AA	Atom (AA)	Occupancy	Atom (ligand)	AA	Atom (AA)	Occupancy	Atom (ligand)	AA	Atom (AA)	Occupancy
WT	O2	Y29	OH	48.8%	C16	F125	CD2	43.5%	[O2,O3]	R120	[NE,NH1,NH2]	99.8%
	O3	Y29	OH	50.8%	C16	F125	CD1	26.6%	N2	E121	[OE1,OE2]	100.0%
	O2	S105	OG	58.2%	C22	F125	CZ	58.6%	N2	E294	[OE1,OE2]	43.4%
	O3	S105	OG	35.1%	C24	F125	CB	47.8%				
	O3	T109	OG1	26.6%	C16	F128	CD1	30.0%				
	N2	R120	NH1	32.2%	C3	V194	CG1	28.6%				
					C21	V209	CG1	31.1%				
					C19	L213	CD1	34.9%				
					C13	W269	CZ3	35.9%				
					C12	L276	CD1	49.7%				
					C3	A293	CB	55.4%				
					C6	L297	CD2	47.0%				
M124$^{3.32}$T	O2	Y29	OH	50.3%	C16	F125	CD2	30.1%	[O2,O3]	R120	[NE,NH1,NH2]	100.0%
	O3	Y29	OH	47.9%	C24	F125	CB	48.5%	N2	E121	[OE1,OE2]	100.0%
	N2	N101	ND2	26.2%	C22	F125	CZ	59.0%	N2	E294	[OE1,OE2]	54.6%
	O3	S105	OG	26.9%	C16	F125	CD1	47.0%				
	O2	S105	OG	25.6%	C15	F125	CE1	30.6%				
	N2	R120	NH1	28.8%	C16	F128	CB	25.2%				
					C20	L174	CD1	27.3%				
					C3	V194	CG1	27.8%				
					C21	V209	CG1	34.1%				
					C19	L213	CD1	32.1%				
					C13	W269	CZ3	35.3%				
					C12	L276	CD1	40.3%				
					C3	A293	CB	53.5%				
					C3	E294	CG	29.6%				
V132$^{3.40}$M	O2	Y29	OH	25.4%	C16	F125	CD2	27.7%	[O2,O3]	K34	NZ	29.3%
	O3	Y29	OH	47.6%	C16	F125	CD1	40.9%	[O2,O3]	R120	[NE,NH1,NH2]	78.7%
	O2	S105	OG	52.0%	C22	F125	CZ	49.5%	N2	E121	[OE1,OE2]	98.2%
	N2	R120	NH1	27.6%	C24	F125	CB	38.3%	N2	E294	[OE1,OE2]	41.8%

					C16	F128	CD1	31.8%				
					C16	F128	CB	31.8%				
					C19	M132	CB	36.3%				
					C3	V194	CG1	34.8%				
					C10	L195	CD2	25.5%				
					C21	V209	CG1	31.3%				
					C12	L276	CD1	29.9%				
					C3	A293	CB	47.1%				
					C6	L297	CD2	38.4%				
F205$^{5.42}$L	O2	Y29	OH	49.7%	C16	F125	CD1	54.8%	[O2,O3]	K34	NZ	26.6%
	O3	Y29	OH	38.8%	C24	F125	CB	58.6%	[O2,O3]	R120	[NE,NH1,NH2]	100.0%
	N2	N101	ND2	27.6%	C22	F125	CZ	57.2%	N2	E121	[OE1,OE2]	100.0%
	N2	N101	OD1	27.0%	C15	F125	CE1	31.9%	N2	E294	[OE1,OE2]	56.6%
	O3	S105	OG	55.8%	C16	F128	CD1	34.2%				
	O2	S105	OG	32.1%	C3	V194	CG1	32.8%				
	N2	R120	NH1	35.8%	C10	L195	CD2	26.1%				
					C21	V209	CG1	38.4%				
					C19	L213	CD1	27.0%				
					C13	W269	CZ3	34.9%				
					C12	L276	CD1	48.5%				
					C3	A293	CB	52.0%				
					C3	E294	CG	29.7%				
					C6	L297	CD2	45.1%				
T207$^{5.44}$I	O2	Y29	OH	32.9%	C22	F125	CZ	63.4%	[O2,O3]	R120	[NE,NH1,NH2]	100.0%
	O3	Y29	OH	59.4%	C24	F125	CB	44.0%	N2	E121	[OE1,OE2]	100.0%
	N2	N101	ND2	28.3%	C16	F125	CD1	52.9%	N2	E294	[OE1,OE2]	43.2%
	O3	S105	OG	29.8%	C15	F125	CE1	30.8%				
	O2	S105	OG	78.7%	C16	F128	CD1	35.4%				
	O2	T109	OG1	30.5%	C3	V194	CG1	31.7%				
	N2	R120	NH1	36.4%	C10	L195	CD2	26.0%				
					C21	V209	CG1	36.4%				
					C13	W269	CZ3	29.6%				
					C12	W269	CZ3	27.0%				
					C12	L276	CD1	41.3%				
					C3	A293	CB	53.6%				

					C6	L297	CD2	43.8%				
T211$^{5.48}$P	O2	Y29	OH	67.5%	C24	F125	CB	51.2%	[O2,O3]	R120	[NE,NH1,NH2]	99.8%
	N2	N101	ND2	25.4%	C22	F125	CZ	58.6%	N2	E121	[OE1,OE2]	100.0%
	O3	S105	OG	72.9%	C16	F125	CD1	44.2%	N2	E294	[OE1,OE2]	42.2%
	O3	T109	OG1	27.0%	C15	F125	CE1	34.1%				
	N2	R120	NH1	28.2%	C16	F128	CD1	29.2%				
					C3	V194	CG1	32.1%				
					C10	L195	CD2	26.2%				
					C21	V209	CG1	25.9%				
					C13	W269	CZ3	35.5%				
					C12	W269	CZ3	27.4%				
					C12	L276	CD1	55.0%				
					C3	A293	CB	47.5%				
					C6	L297	CD2	40.5%				
A293$^{7.35}$T	O2	Y29	OH	49.9%	C16	F125	CD2	43.0%	[O2,O3]	R120	[NE,NH1,NH2]	100.0%
	O3	Y29	OH	49.4%	C16	F125	CD1	28.2%	N2	E121	[OE1,OE2]	100.0%
	N2	N101	OD1	29.9%	C24	F125	CB	51.8%	N2	E294	[OE1,OE2]	42.9%
	O3	S105	OG	55.6%	C22	F125	CZ	62.6%				
	O2	S105	OG	67.6%	C15	F125	CE2	26.7%				
	O3	T109	OG1	25.6%	C16	F128	CD1	37.1%				
	O2	T109	OG1	27.1%	C3	V194	CG1	37.2%				
	N2	R120	NH1	37.7%	C21	V209	CG1	34.1%				
					C12	W269	CZ3	29.6%				
					C12	L276	CD1	42.4%				
					C3	E294	CG	30.3%				
					C6	L297	CD2	45.9%				
A293$^{7.35}$V	O2	Y29	OH	48.4%	C16	F125	CD2	33.3%	[O2,O3]	K34	NZ	29.3%
	O3	Y29	OH	49.9%	C16	F125	CD1	40.1%	[O2,O3]	R120	[NE,NH1,NH2]	100.0%
	N2	N101	ND2	34.2%	C24	F125	CB	58.4%	N2	E121	[OE1,OE2]	100.0%
	O2	S105	OG	55.1%	C22	F125	CZ	61.5%	N2	E294	[OE1,OE2]	64.1%
	O3	S105	OG	34.8%	C15	F125	CE1	27.4%				
	O3	T109	OG1	26.0%	C16	F128	CD1	30.2%				
	N2	R120	NH1	37.3%	C21	V209	CG1	35.9%				
					C19	L213	CD1	26.2%				
					C13	W269	CZ3	30.3%				

C12	W269	CZ3	29.4%
C12	L276	CD1	35.1%
C3	V293	CG1	29.8%
C3	E294	CG	37.2%
C6	L297	CD2	42.5%

Table S6 – A detailed interaction analysis for all systems with bound ozanimod using PLIP. For better clarity of the obtained results, we focused on the interactions with ≥ 25.0% occupancy throughout each MD simulation. The ligand-atom numbering for ozanimod is presented in Figure S57.

System	Hydrogen bonds				Hydrophobic interactions				Pi-stack			
	Atom (ligand)	AA	Atom (AA)	Occupancy	Atom (ligand)	AA	Atom (AA)	Occupancy	Atom (ligand)	AA	Atom (AA)	Occupancy
WT	N1	E121	OE1	49.5%	C23	L174	CD2	37.5%	[C5,C6,N2,N3,O2]	F125	[CG,CD1,CE1,CZ,CD2,CE2]	25.8%
	N1	E121	OE2	59.8%	C2	V194	CG1	30.2%				
	O1	P196	O	50.3%	C23	V209	CG1	42.1%				
					C19	W269	CZ3	26.9%				
					C4	L297	CD1	36.6%				
M124$^{3.32}$T	N1	E121	OE1	54.2%	C8	F128	CB	25.0%				
	N1	E121	OE2	52.5%	C23	L174	CD2	39.6%				
	O1	P196	O	28.5%	C2	V194	CG1	42.9%				
					C14	L195	CD2	25.7%				
					C4	L195	CD2	26.0%				
					C23	V209	CG1	51.7%				
					C3	L272	CD1	25.1%				
					C4	L297	CD1	31.0%				
V132$^{3.40}$M	N1	E121	OE1	80.4%	C7	F125	CE1	27.8%				
	N1	E121	OE2	43.7%	C23	L174	CD2	37.0%				
	O1	P196	O	50.5%	C2	V194	CG1	31.6%				
					C4	L195	CD2	29.5%				
					C23	V209	CG1	41.4%				
					C4	L297	CD1	33.2%				
F205$^{5.42}$L	N1	E121	OE1	52.2%	C7	F125	CE1	32.4%	[C5,C6,N2,N3,O2]	F125	[CG,CD1,CE1,CZ,CD2,CE2]	27.3%
	N1	E121	OE2	44.1%	C8	F128	CB	31.6%				
					C22	V132	CG2	29.3%				
					C23	L174	CD2	39.3%				
					C2	V194	CG1	42.5%				
					C4	L195	CD2	27.7%				
					C23	V209	CG1	40.4%				
					C3	L272	CD1	28.9%				
T207$^{5.44}$I	N1	E121	OE2	62.5%	C8	F128	CB	30.9%				
	N1	E121	OE1	35.6%	C23	L174	CD2	33.8%				
	O1	E294	OE2	28.9%	C2	V194	CG1	48.0%				

	O1	E294	OE1	26.9%	C14	L195	CD2	28.2%
					C4	L195	CD2	26.7%
					C23	V209	CG1	34.1%
					C3	L272	CD1	34.7%
					C4	L297	CD1	26.7%
T211$^{5.48}$P	N1	E121	OE1	54.3%	C23	L174	CD2	32.0%
	N1	E121	OE2	46.0%	C2	V194	CG1	40.9%
	O1	E294	OE2	38.3%	C23	V209	CG1	34.6%
	O1	E294	OE1	34.1%	C3	L272	CD1	34.1%
					C19	L276	CD1	33.9%
A293$^{7.35}$T	N1	E121	OE1	46.0%	C7	F125	CE1	25.8%
	N1	E121	OE2	67.8%	C8	F128	CB	26.4%
					C23	L174	CD2	32.7%
					C2	V194	CG1	27.7%
					C23	V209	CG1	46.1%
A293$^{7.35}$V	N1	E121	OE1	65.3%	C7	F125	CE1	26.1%
	N1	E121	OE2	56.7%	C23	L174	CD2	36.0%
	O1	P196	O	25.0%	C2	V194	CG1	29.8%
					C4	L195	CD2	27.6%
					C23	V209	CG1	49.1%
					C22	L213	CD1	26.2%

Table S7 – A detailed interaction analysis for all systems with bound ponesimod using PLIP. For better clarity of the obtained results, we focused on the interactions with ≥ 25.0% occupancy throughout each MD simulation. The ligand-atom numbering for ponesimod is presented in Figure S58.

System	Hydrogen bonds				Hydrophobic interactions			
	Atom (ligand)	AA	Atom (AA)	Occupancy	Atom (ligand)	AA	Atom (AA)	Occupancy
WT	O2	S129	N	49.6%	CL	M124	CB	26.0%
	N2	C206	O	26.3%	C17	F125	CE1	34.1%
					C7	L128	CD1	48.3%
					C15	V132	CB	27.6%
					C15	V132	CG2	34.6%
					C15	F210	CD1	29.9%
					C19	L213	CD1	50.8%
					C23	W269	CZ3	31.6%
					C23	W269	CE3	34.8%
					C6	W269	CH2	32.5%
					C14	L276	CD1	27.3%
$M124^{3.32}T$	O2	S129	N	41.5%	C17	T124	CG2	32.1%
					C7	F125	CD2	30.2%
					C17	F125	CE2	25.3%
					C7	L128	CD1	45.9%
					C15	V132	CG2	36.9%
					C15	V132	CB	28.2%
					CL	L195	CD2	25.7%
					C19	L213	CD1	45.3%
					C23	W269	CE3	46.0%
					C6	W269	CH2	29.2%
					C14	L276	CD1	39.9%
$V132^{3.40}M$	O2	S129	N	33.4%	CL	E121	CB	38.0%
	N2	C206	O	27.8%	C17	M124	CB	25.9%
					CL	M124	CB	26.6%
					C17	F125	CE1	38.6%
					C7	L128	CD1	50.5%
					C15	M132	CB	50.0%
					CL	L195	CD2	34.0%
					C23	W269	CZ3	32.2%

					C23	W269	CE3	30.4%
					C14	L276	CD1	28.5%
F205$^{5.42}$L	O2	S129	N	39.7%	CL	E121	CB	51.4%
	N2	C206	O	28.7%	CL	M124	CB	27.3%
	O3	E294	OE1	25.7%	C17	F125	CE1	45.7%
					C7	L128	CD1	49.6%
					C15	V132	CG2	39.9%
					CL	L195	CD2	37.0%
					C23	F210	CD2	26.2%
					C15	F210	CD1	41.3%
					C19	L213	CD1	46.9%
					C6	W269	CH2	33.4%
					C23	W269	CZ3	33.9%
					C23	W269	CE3	34.0%
					C14	L276	CD1	28.3%
					C21	L297	CD1	35.2%
T207$^{5.44}$I	O2	S129	N	49.1%	CL	E121	CB	35.8%
	N2	C206	O	29.5%	CL	E121	CG	28.0%
	O3	A293	O	34.5%	CL	M124	CB	44.0%
	O4	E294	OE1	27.1%	C17	F125	CE1	41.7%
	O3	E294	OE1	35.5%	C17	F125	CE2	25.7%
					C7	L128	CD1	56.2%
					C15	V132	CG2	38.0%
					C15	V132	CB	28.2%
					CL	L195	CD2	40.7%
					C14	I207	CG1	27.7%
					C15	F210	CD1	25.7%
					C19	L213	CD1	48.2%
					C23	W269	CE3	38.4%
					C6	W269	CH2	30.2%
					C23	W269	CZ3	31.4%
T211$^{5.48}$P	O2	S129	N	37.0%	CL	E121	CB	33.8%
	N2	C206	O	26.6%	CL	M124	CB	40.5%
	O3	A293	O	32.0%	C17	F125	CE1	42.4%
	O4	A293	O	30.3%	C7	L128	CD1	40.2%

					C15	V132	CG2	31.2%
					CL	L195	CD2	43.5%
					C14	F210	CB	27.7%
					C19	L213	CD1	49.3%
					C23	W269	CE3	27.4%
					C6	W269	CH2	29.4%
					C23	W269	CZ3	34.2%
A293$^{7.35}$T	O2	S129	N	48.9%	CL	E121	CB	39.3%
	N2	C206	O	31.6%	CL	M124	CB	35.5%
	O3	T293	O	30.1%	C17	F125	CE1	41.2%
					C7	L128	CD1	48.9%
					C15	V132	CG2	34.4%
					C15	V132	CB	27.5%
					CL	L195	CD2	47.1%
					C15	F210	CD1	35.5%
					C19	L213	CD1	55.8%
					C23	W269	CE3	28.7%
					C6	W269	CH2	40.3%
					C23	W269	CZ3	35.1%
A293$^{7.35}$V	O2	S129	N	44.8%	CL	E121	CB	42.3%
	N2	C206	O	29.5%	CL	M124	CB	36.1%
					C12	F125	CD2	26.3%
					C17	F125	CE1	50.6%
					C7	L128	CD1	48.2%
					C15	V132	CB	28.4%
					C15	V132	CG2	35.9%
					CL	L195	CD2	33.5%
					C15	F210	CD1	26.2%
					C19	L213	CD1	51.3%
					C6	W269	CH2	37.9%
					C23	W269	CE3	31.4%
					C23	W269	CZ3	40.8%
					C21	L297	CD1	34.8%

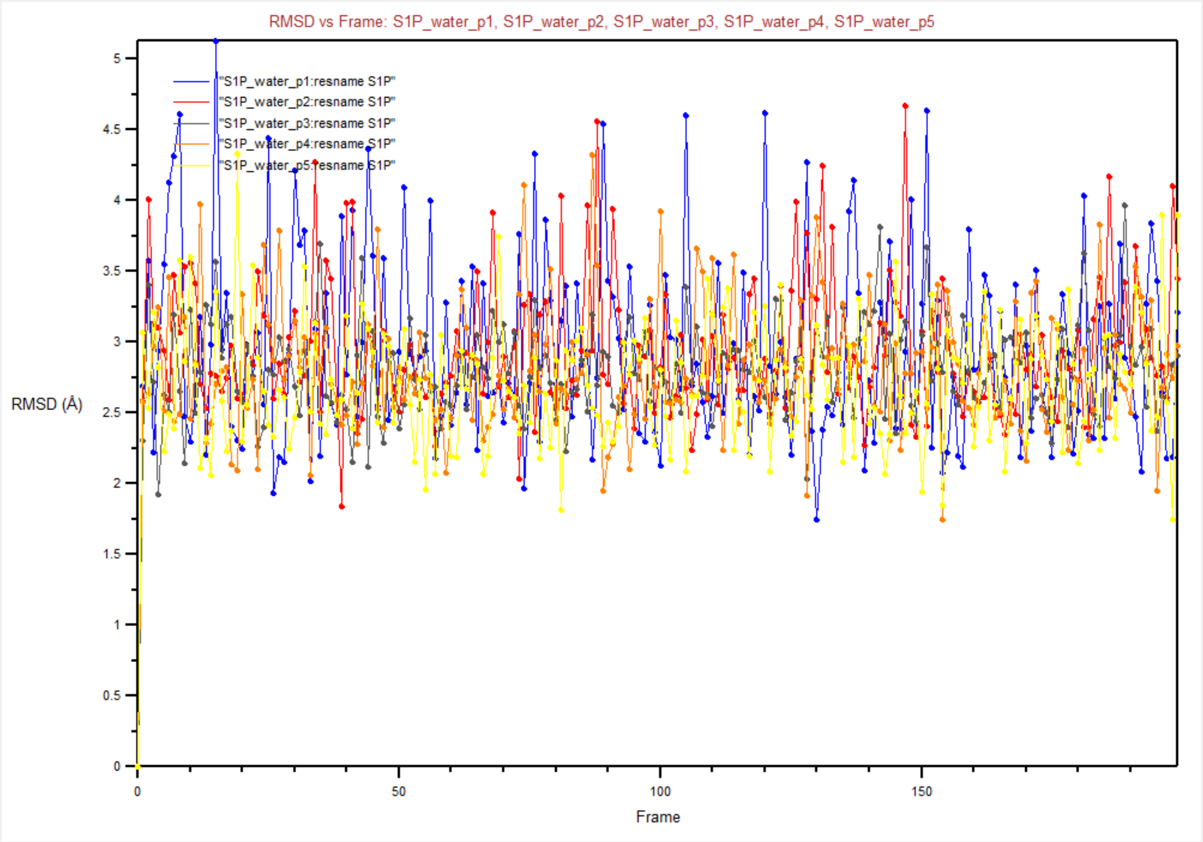

Figure S1 – RMSD of ligand S1P in water in relation to the frames with five parallel simulations. A total of 200 frames coincided with 100 ns of simulation.

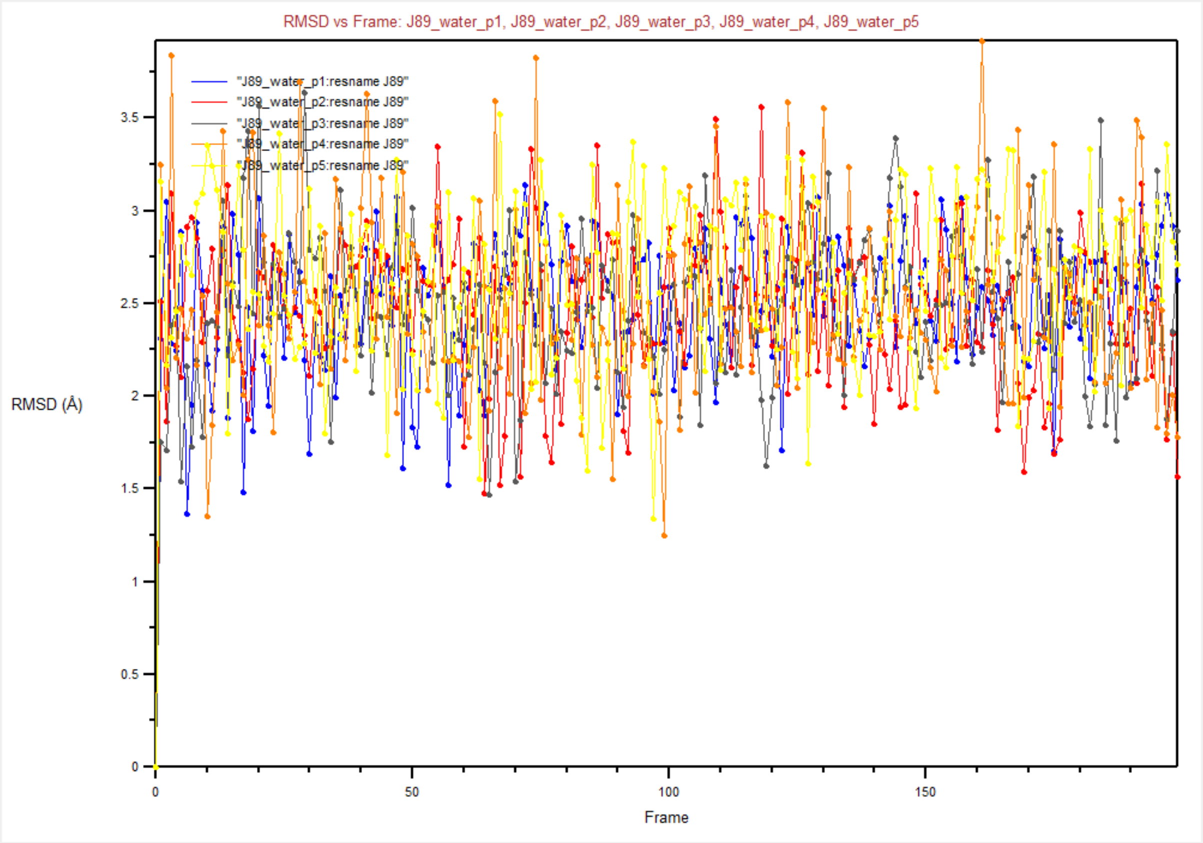

Figure S2 – RMSD of fingolimod in water in relation to the frames with five parallel simulations. A total of 200 frames coincided with 100 ns of simulation.

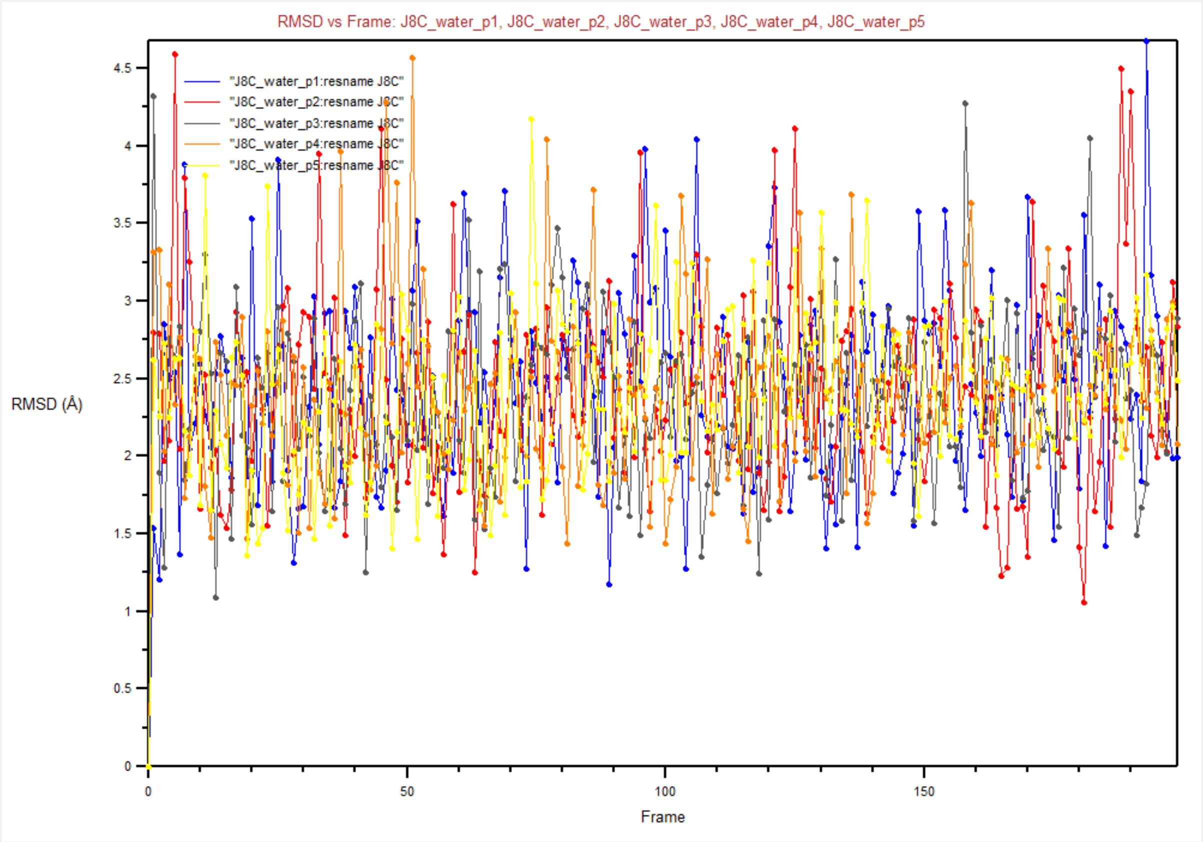

Figure S3 – RMSD of siponimod in water in relation to the frames with five parallel simulations. A total of 200 frames coincided with 100 ns of simulation.

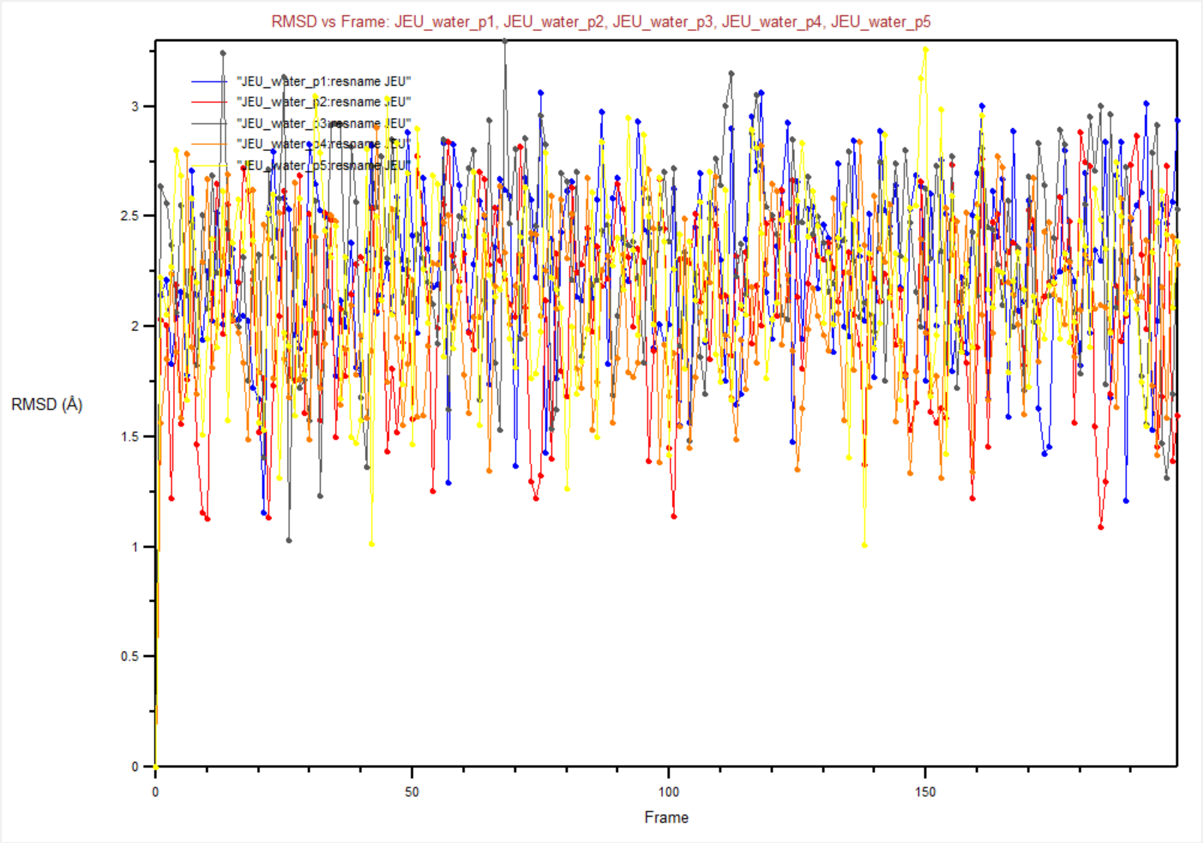

Figure S4 – RMSD of ozanimod in water in relation to the frames with five parallel simulations. A total of 200 frames coincided with 100 ns of simulation.

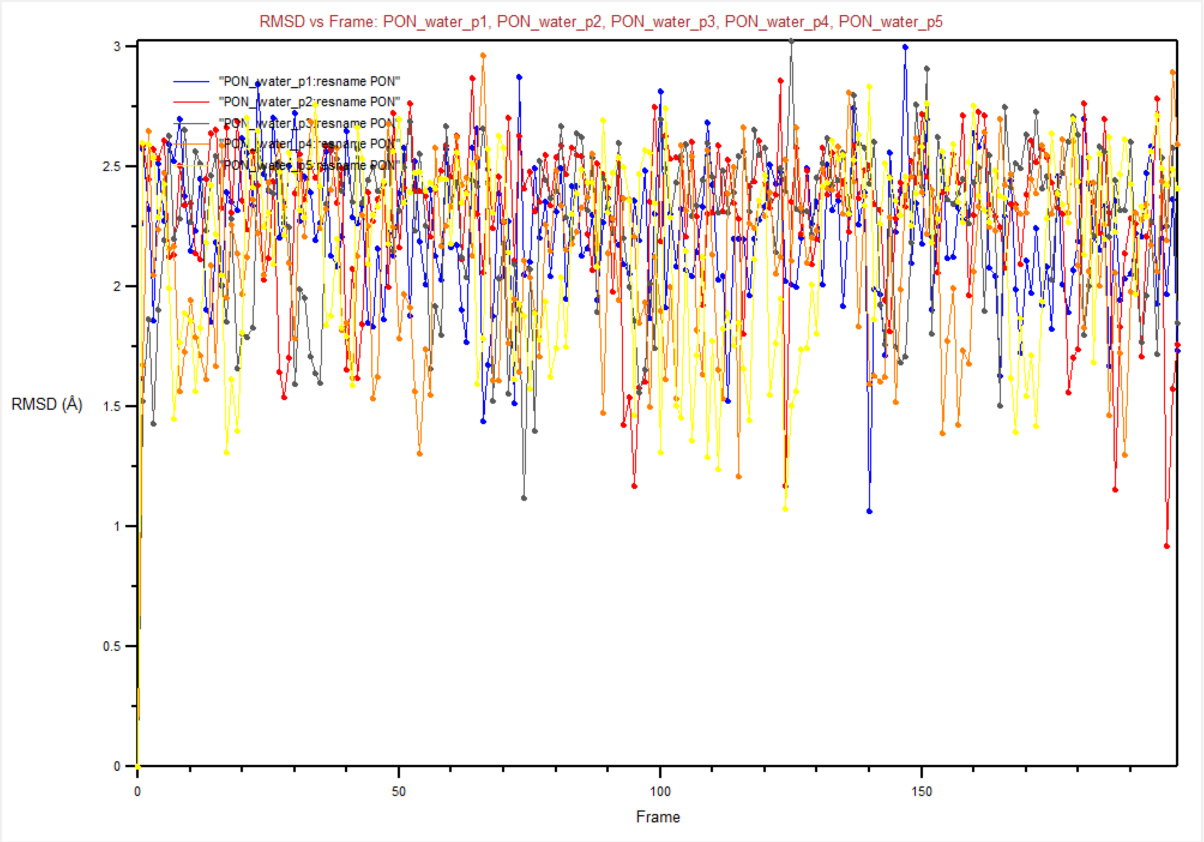

Figure S5 – RMSD of ponesimod in water in relation to the frames with five parallel simulations. A total of 200 frames coincided with 100 ns of simulation.

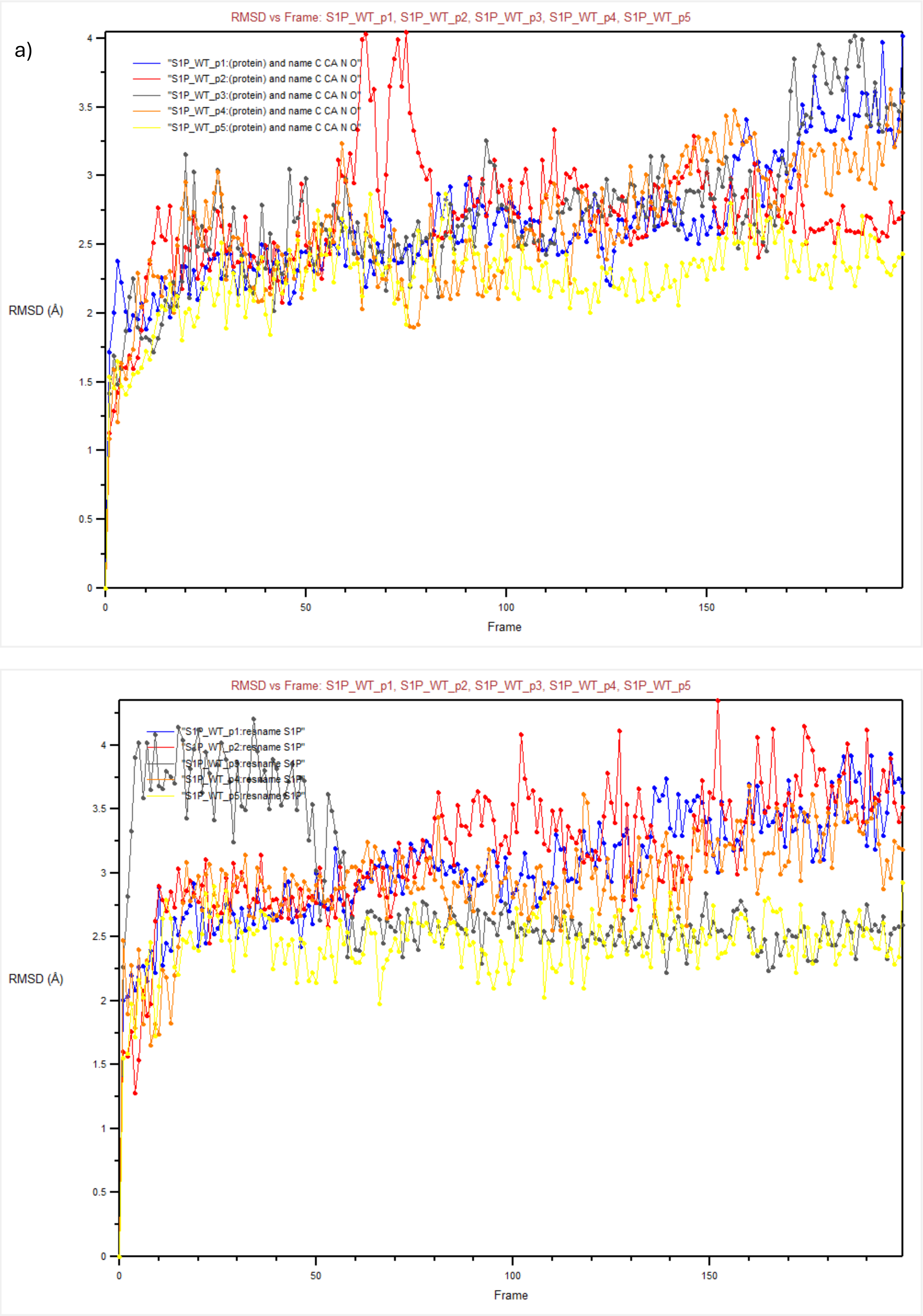

Figure S6 – RMSD of a) wild type S1PR1 protein and b) S1P in relation to the frames with five parallel simulations. A total of 200 frames coincided with 100 ns of simulation.

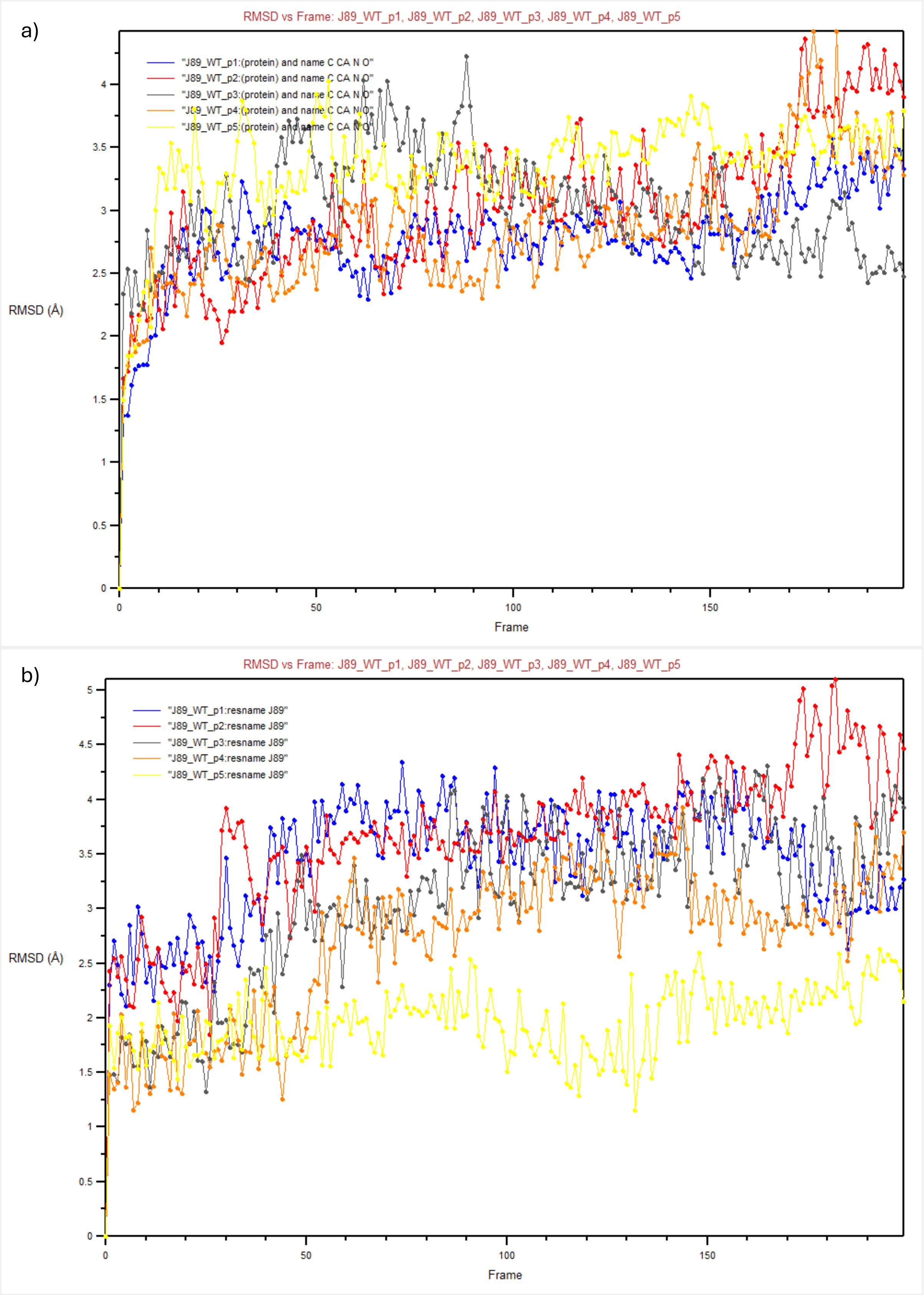

Figure S7 – RMSD of a) wild type S1PR1 protein and b) fingolimod in relation to the frames with five parallel simulations. A total of 200 frames coincided with 100 ns of simulation.

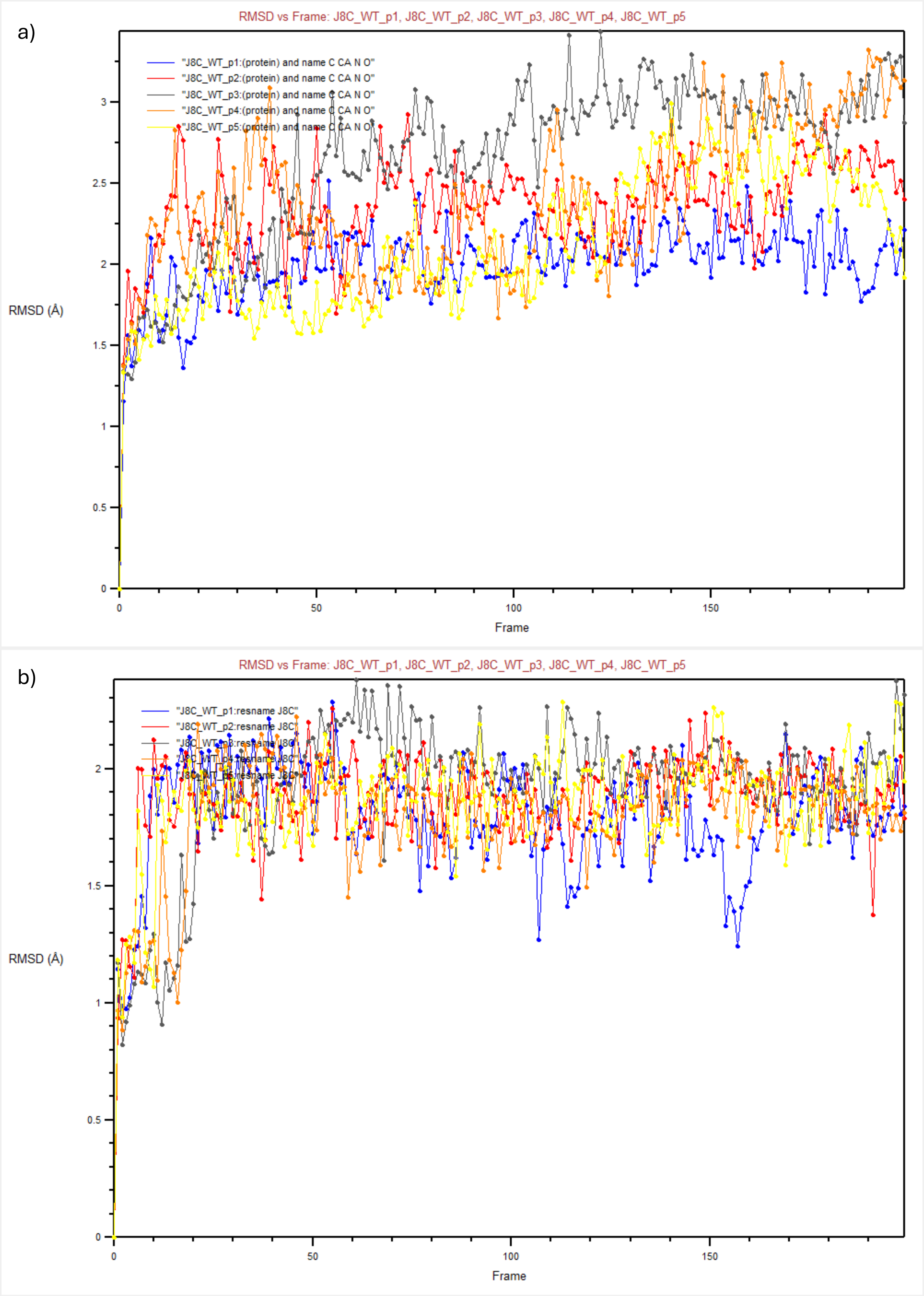

Figure S8 – RMSD of a) wild type S1PR1 protein and b) siponimod in relation to the frames with five parallel simulations. A total of 200 frames coincided with 100 ns of simulation.

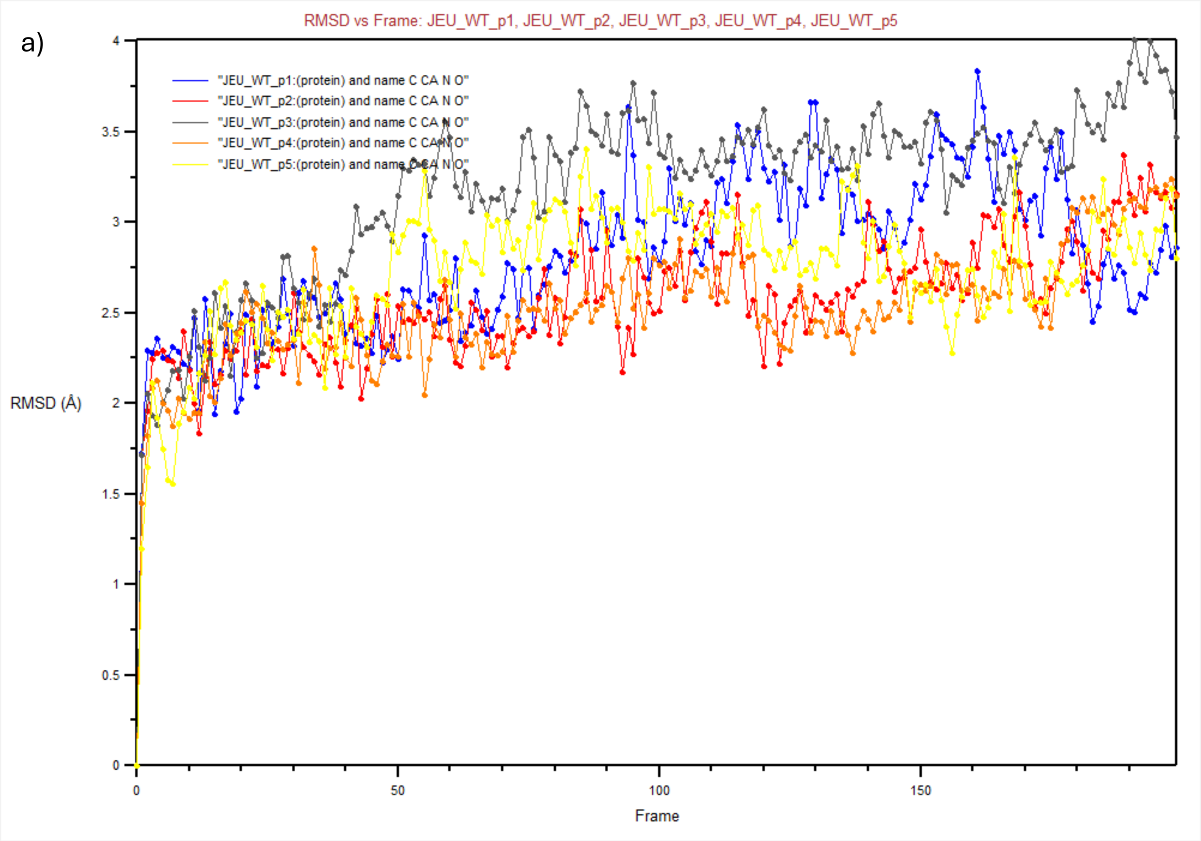

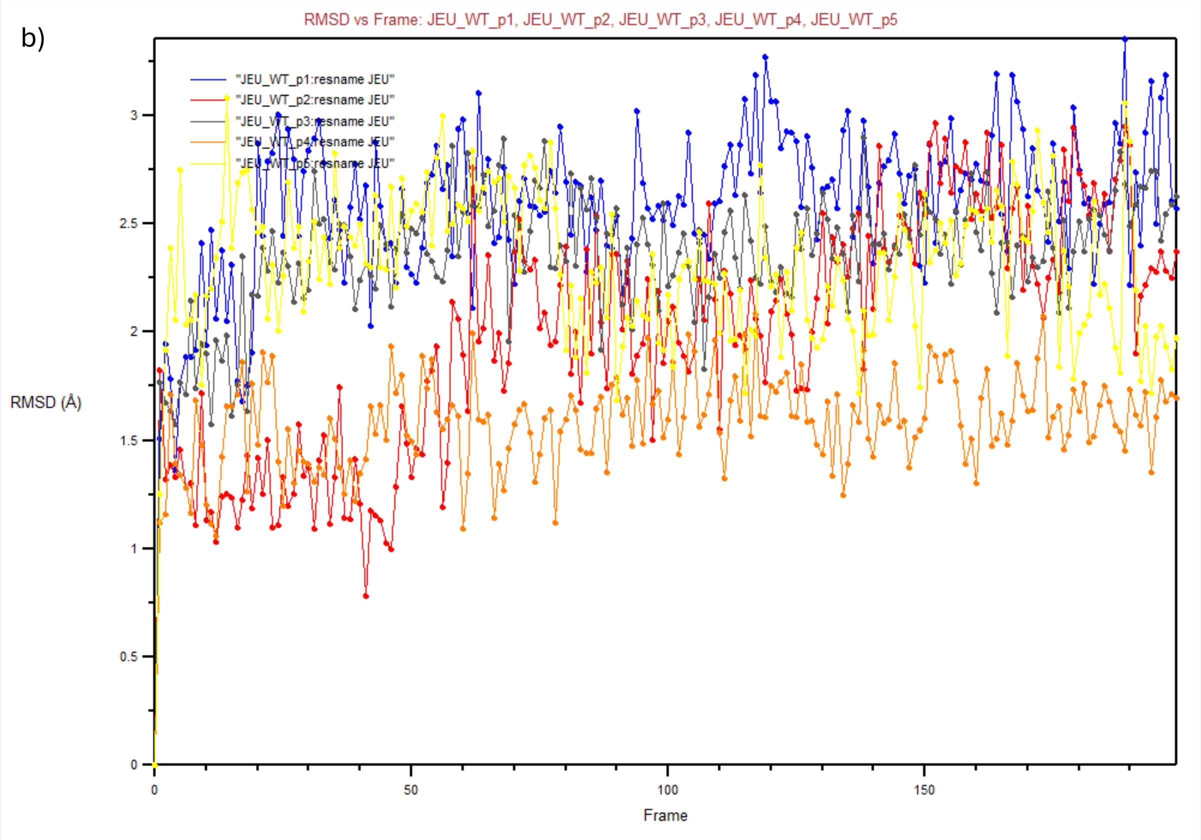

Figure S9 – RMSD of a) wild type S1PR1 protein and b) ozanimod in relation to the frames with five parallel simulations. A total of 200 frames coincided with 100 ns of simulation.

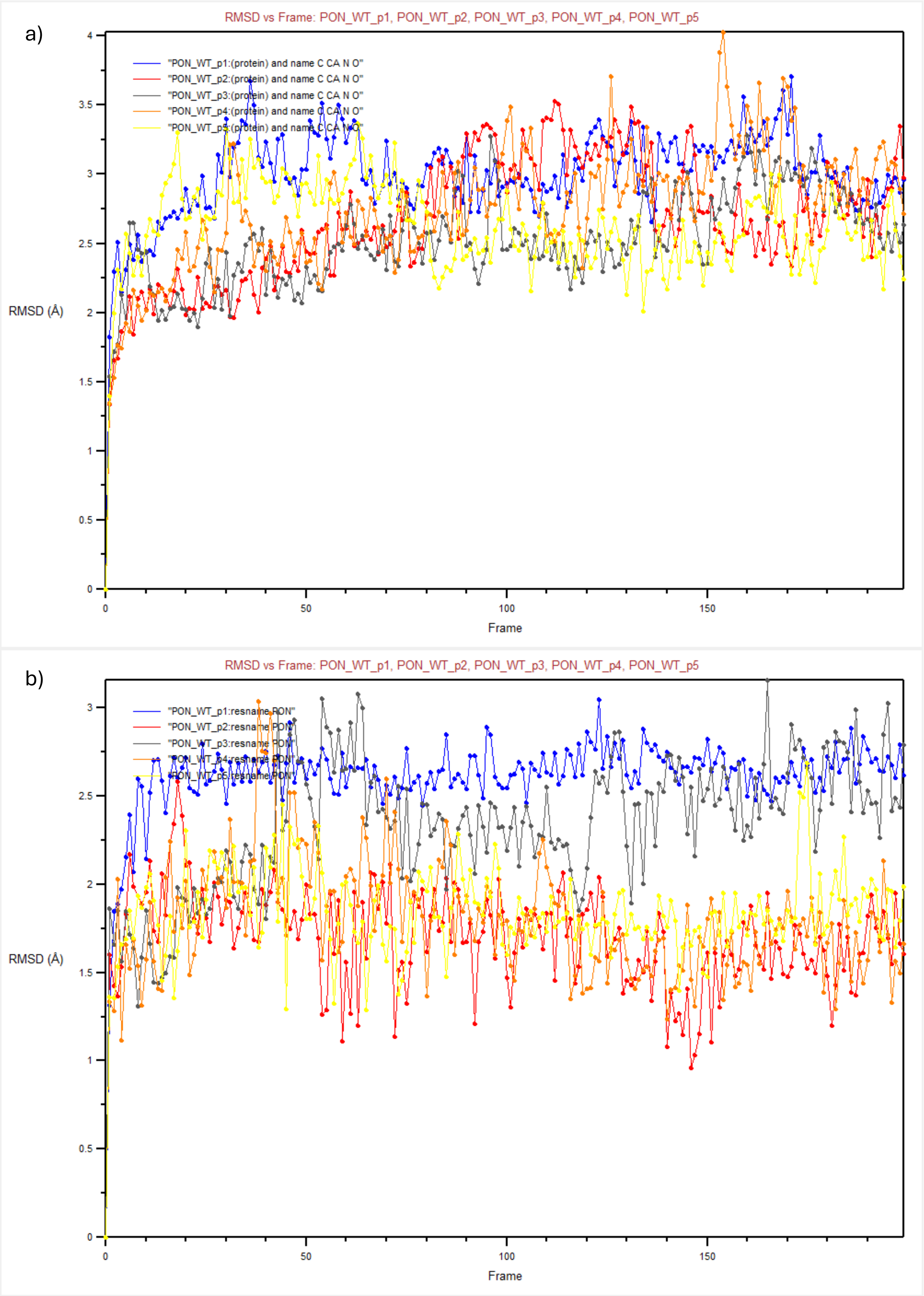

Figure S10 – RMSD of a) wild type S1PR1 protein and b) ponesimod in relation to the frames with five parallel simulations. A total of 200 frames coincided with 100 ns of simulation.

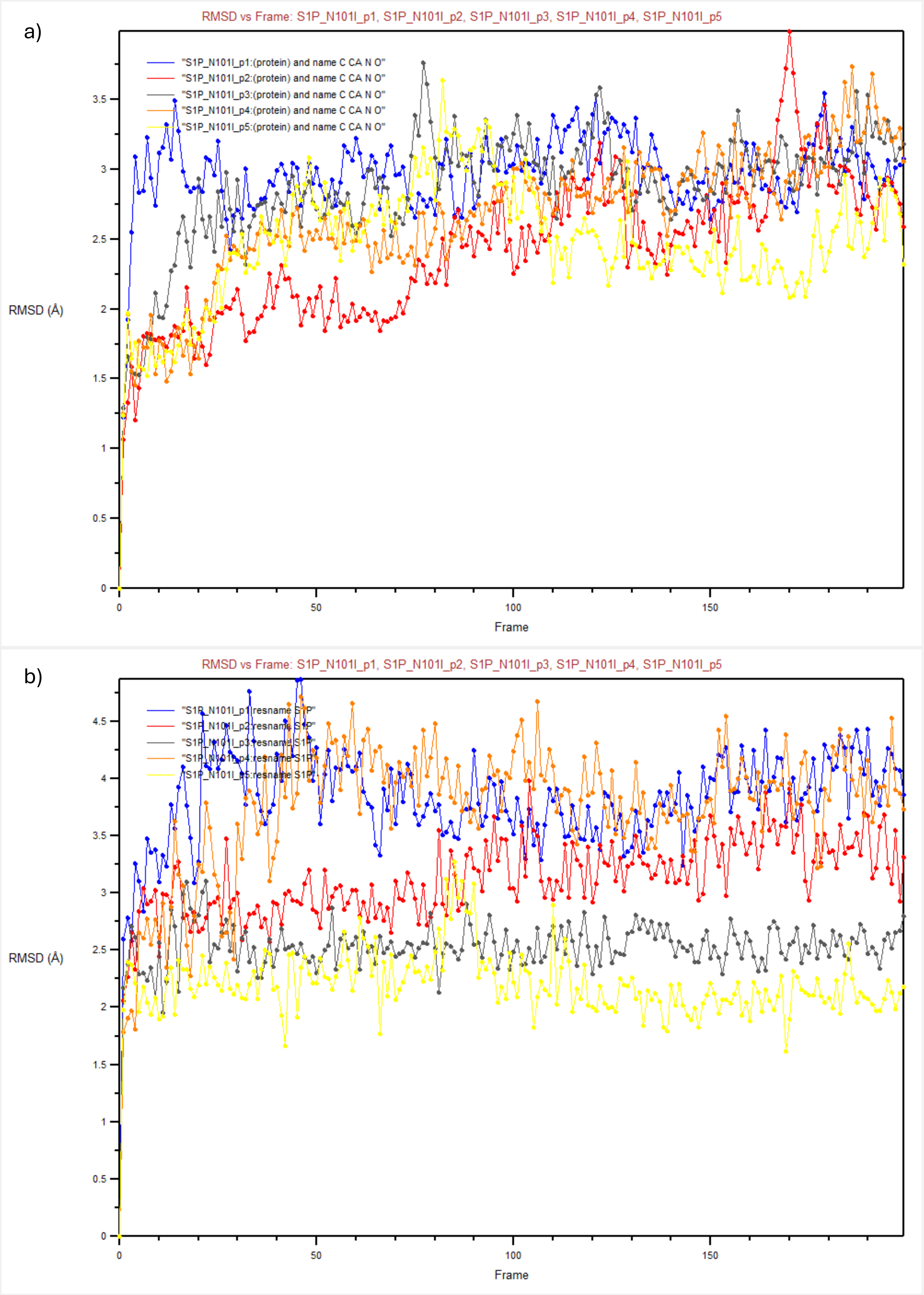

Figure S11 – RMSD of a) protein with N101$^{2.60}$I mutation and b) S1P in relation to the frames with five parallel simulations. A total of 200 frames coincided with 100 ns of simulation.

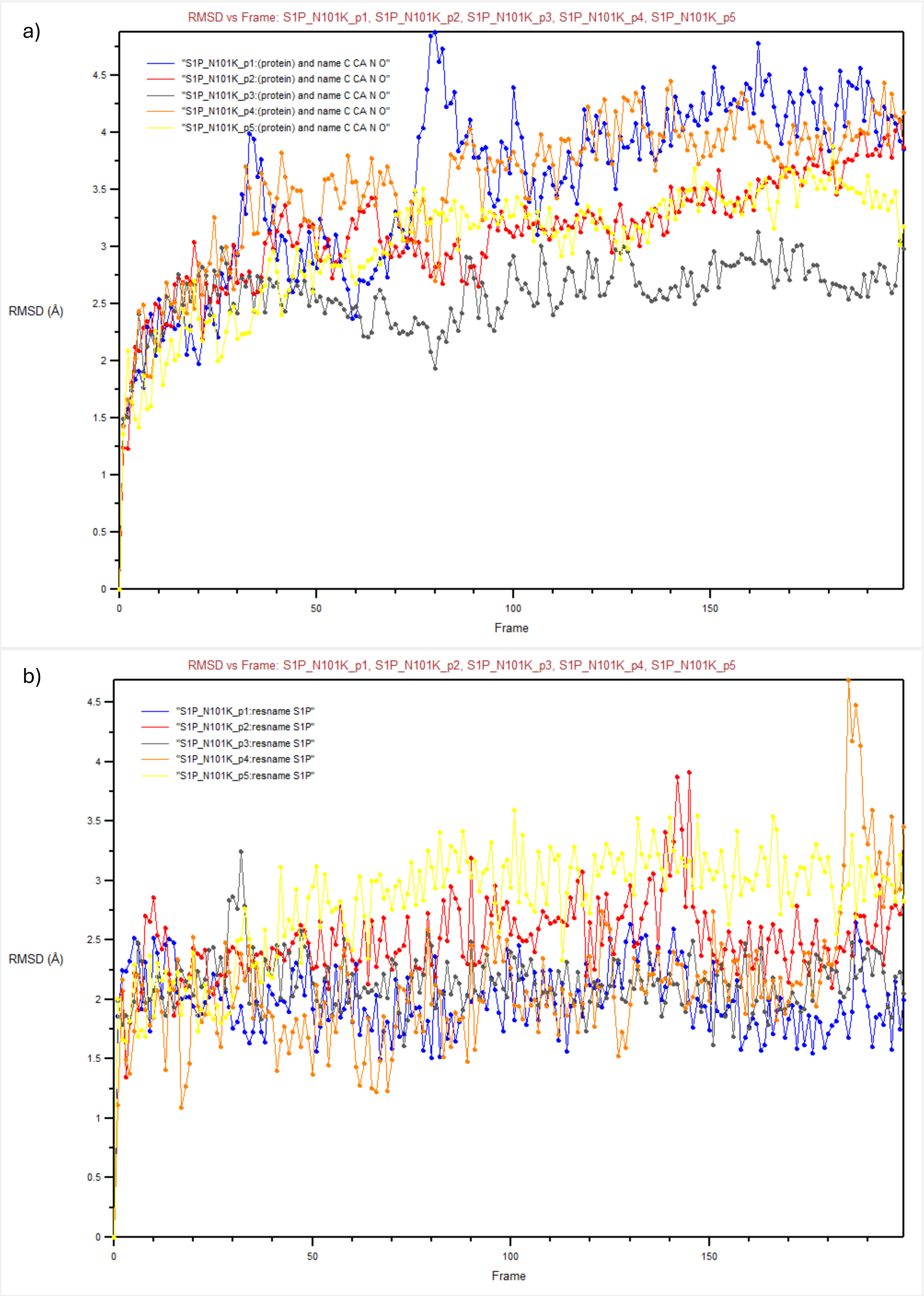

Figure S12 – RMSD of a) protein with N101$^{2.60}$K mutation and b) S1P in relation to the frames with five parallel simulations. A total of 200 frames coincided with 100 ns of simulation.

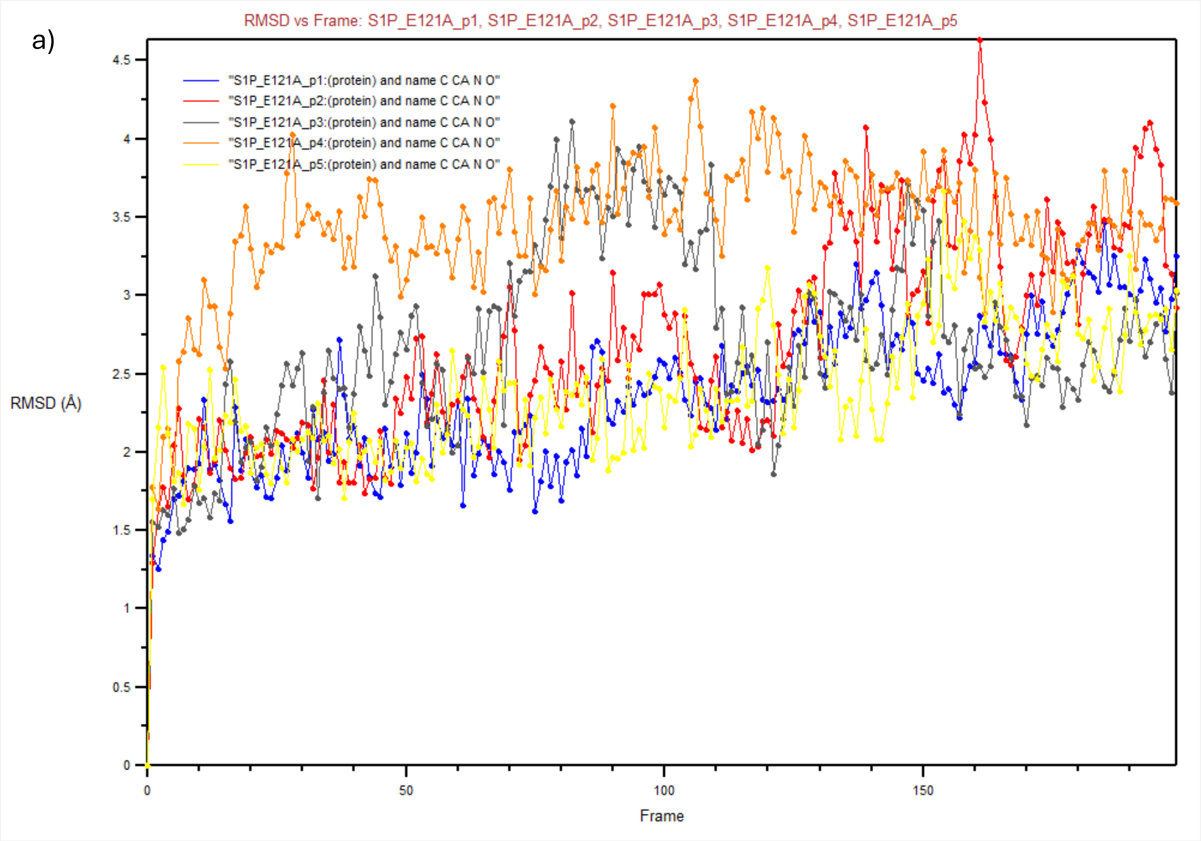

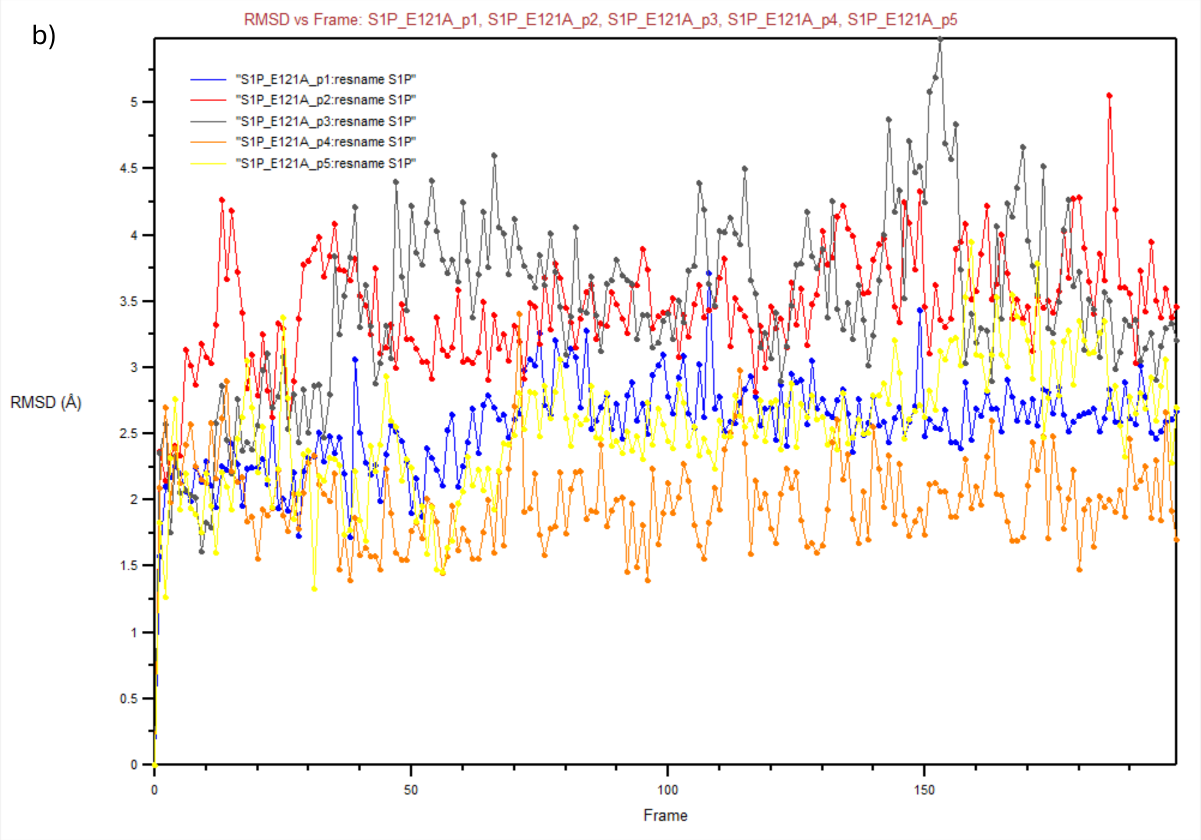

Figure S13 – RMSD of a) protein with E121$^{3.29}$A mutation and b) S1P in relation to the frames with five parallel simulations. A total of 200 frames coincided with 100 ns of simulation.

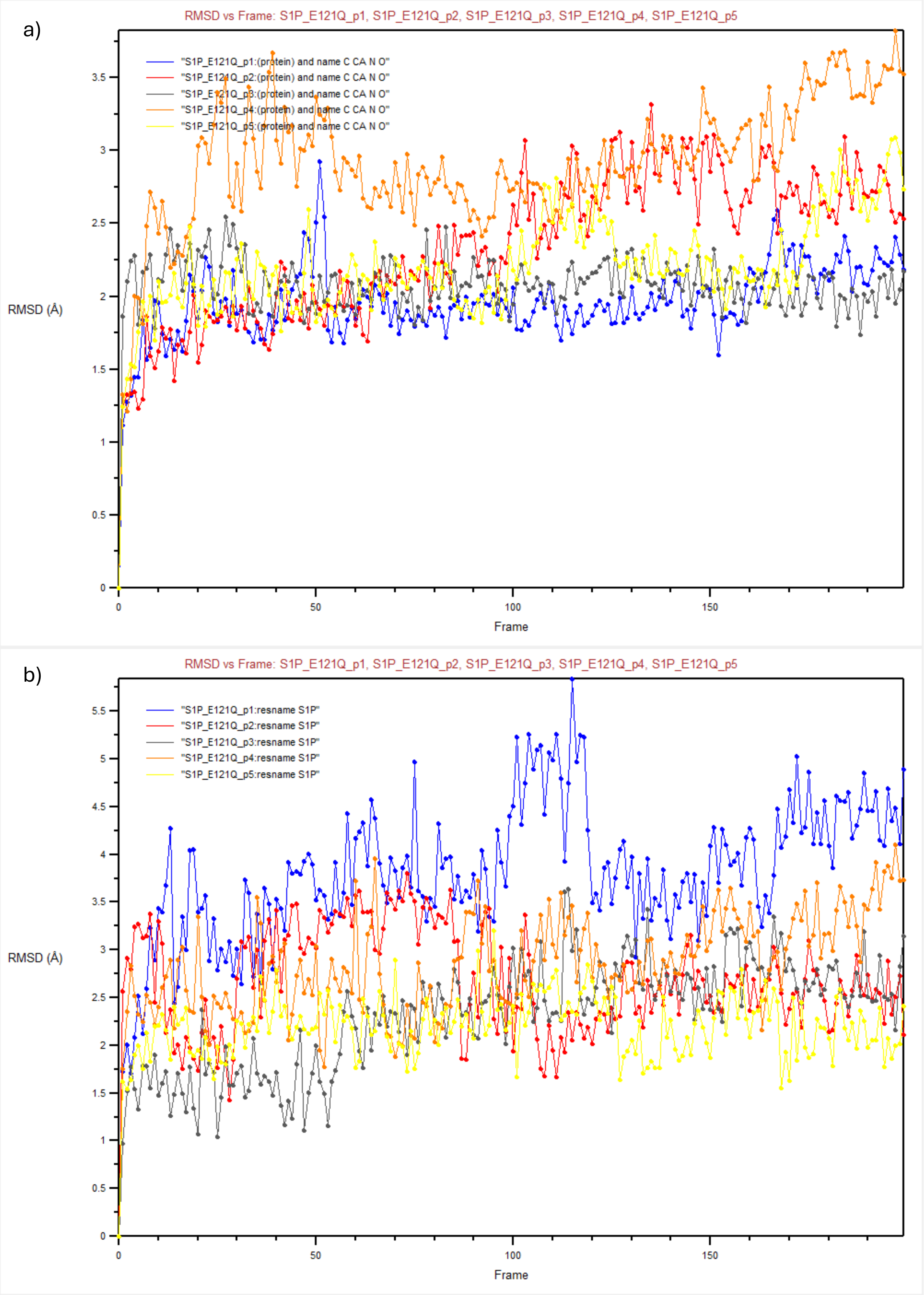

Figure S14 – RMSD of a) protein with E121$^{3.29}$Q mutation and b) S1P in relation to the frames with five parallel simulations. A total of 200 frames coincided with 100 ns of simulation.

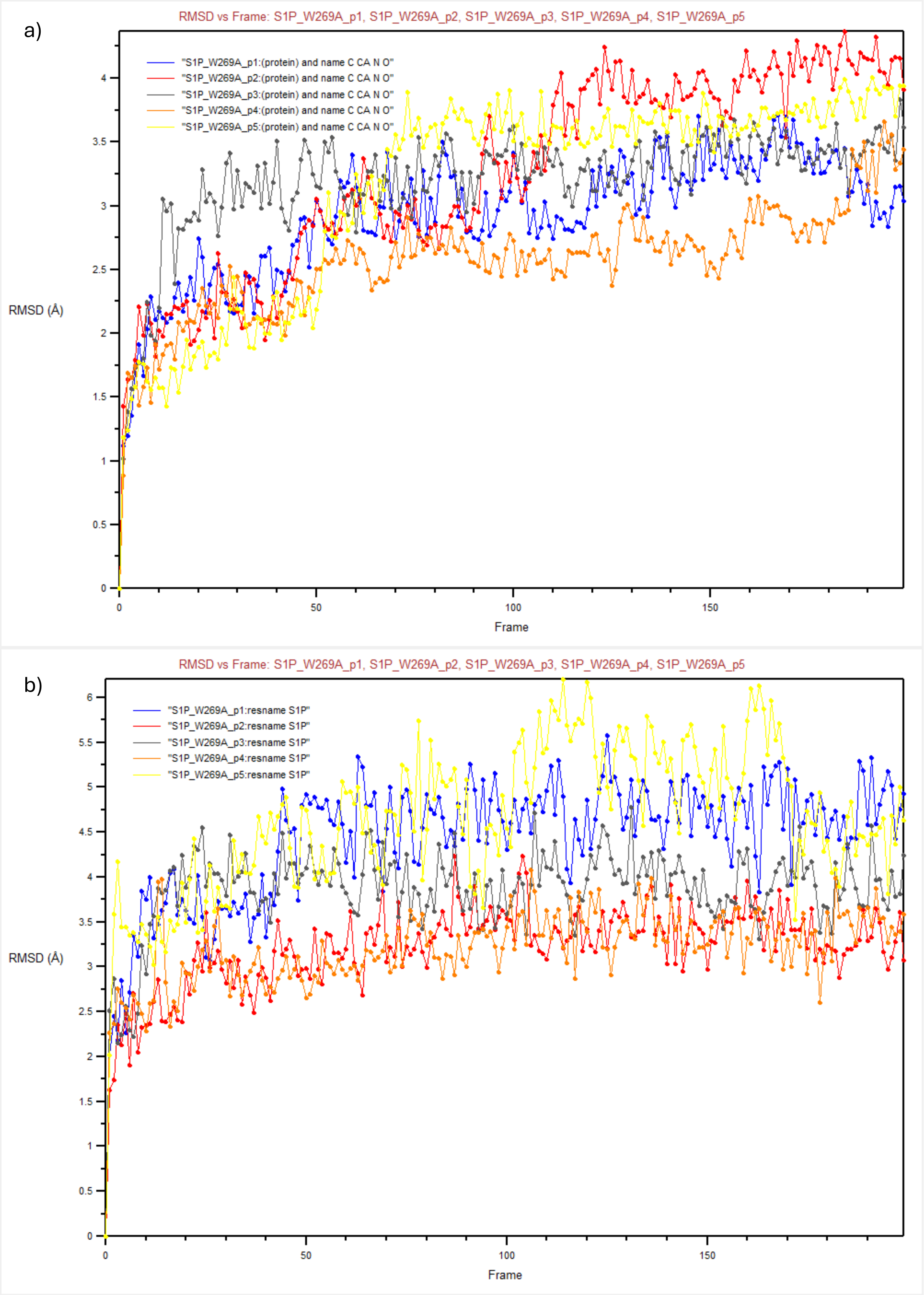

Figure S15 – RMSD of a) protein with W269$^{6.48}$A mutation and b) S1P in relation to the frames with five parallel simulations. A total of 200 frames coincided with 100 ns of simulation.

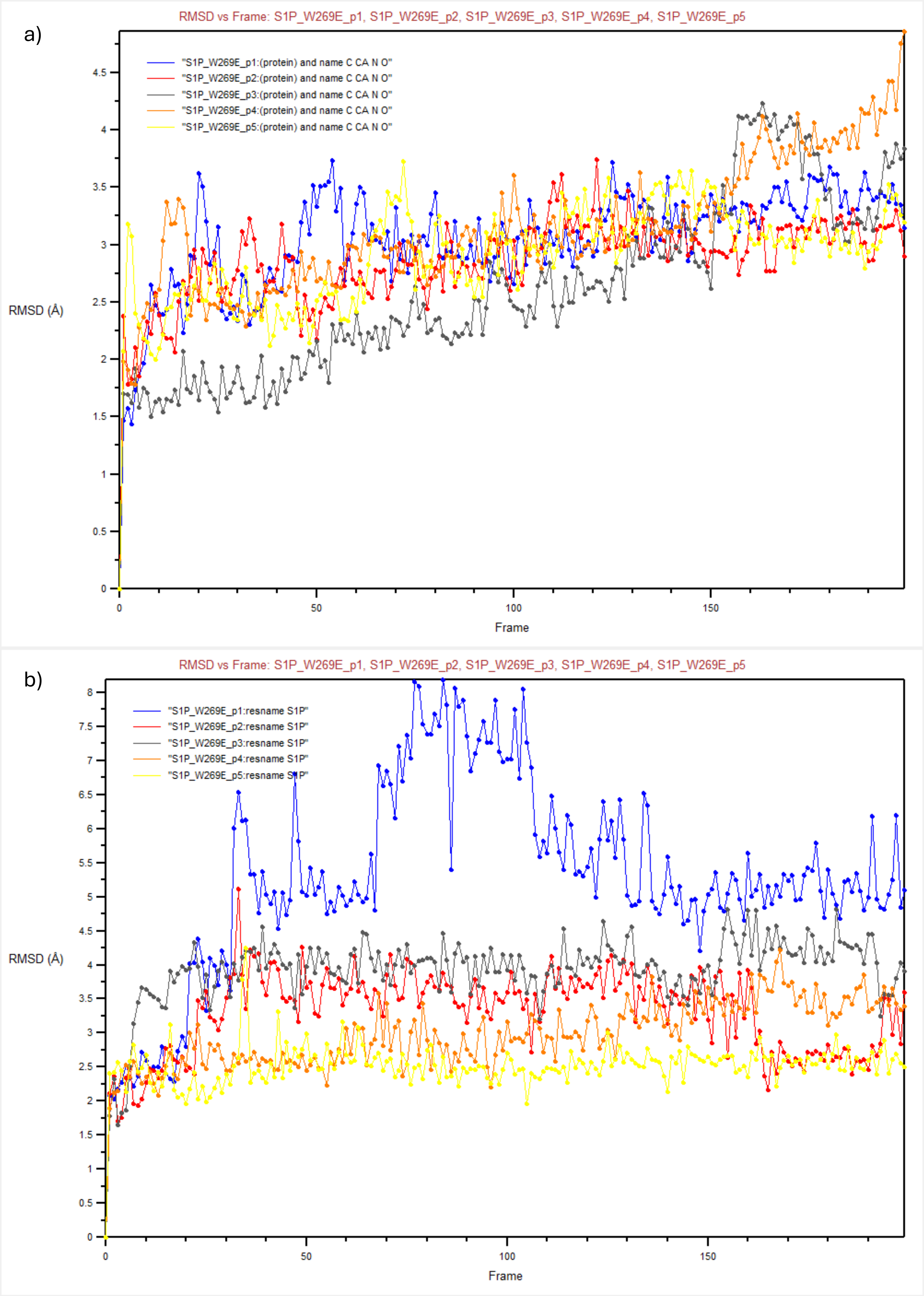

Figure S16 – RMSD of a) protein with W269$^{6.48}$E mutation and b) S1P in relation to the frames with five parallel simulations. A total of 200 frames coincided with 100 ns of simulation.

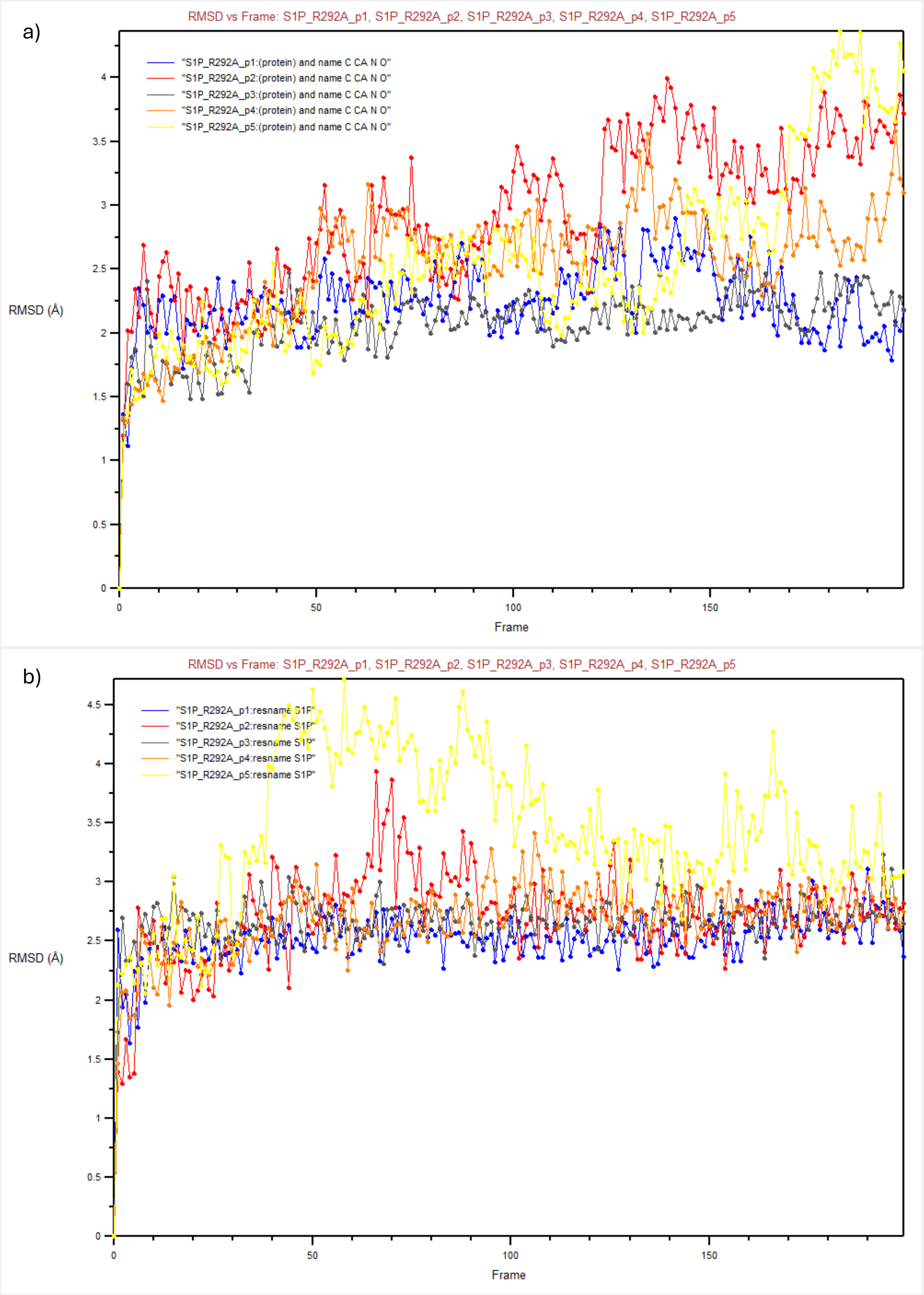

Figure S17 – RMSD of a) protein with R292$^{7.34}$A mutation and b) S1P in relation to the frames with five parallel simulations. A total of 200 frames coincided with 100 ns of simulation.

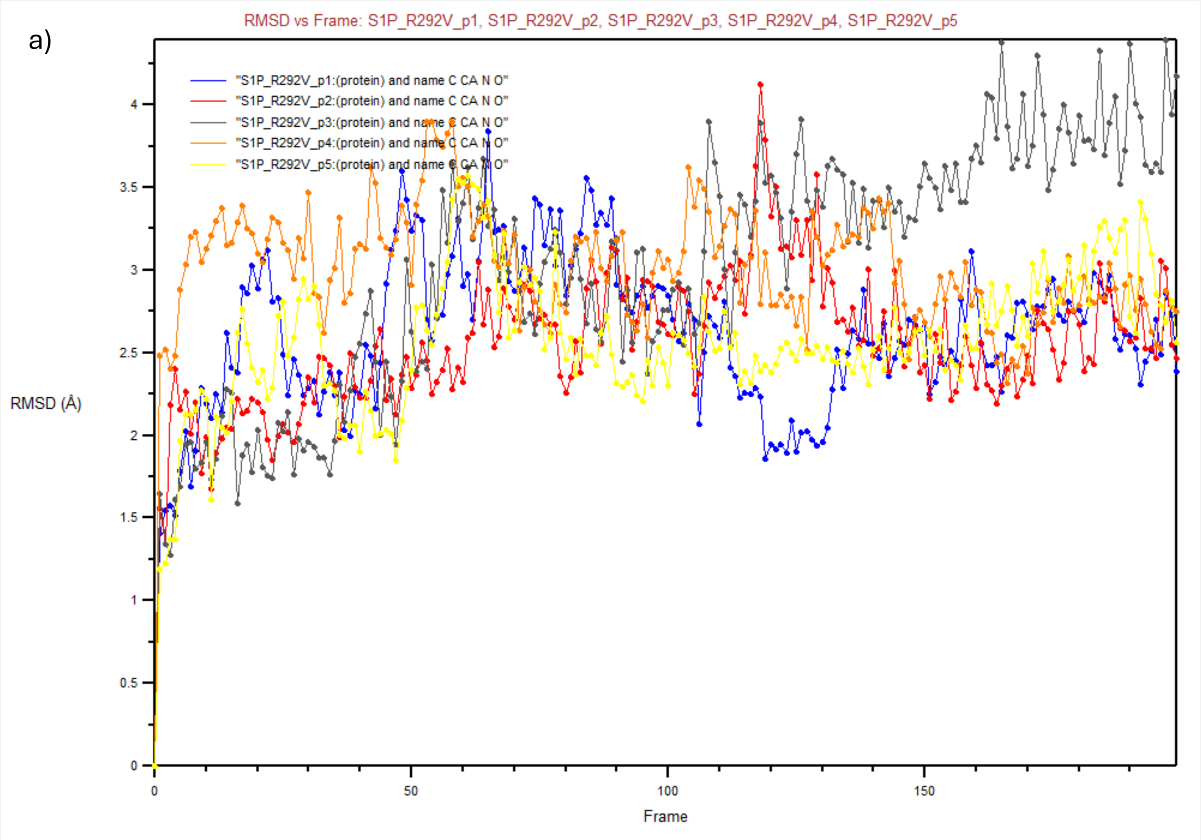

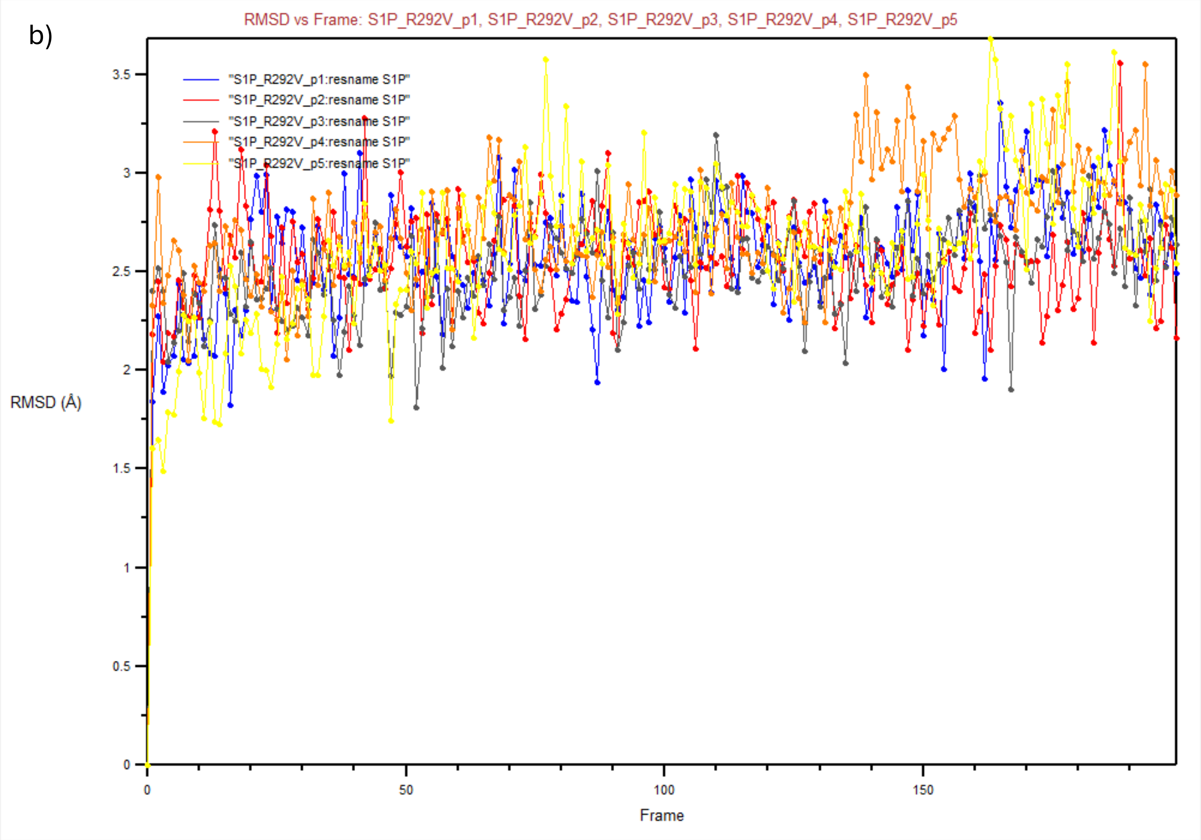

Figure S18 – RMSD of a) protein with R292$^{7.34}$V mutation and b) S1P in relation to the frames with five parallel simulations. A total of 200 frames coincided with 100 ns of simulation.

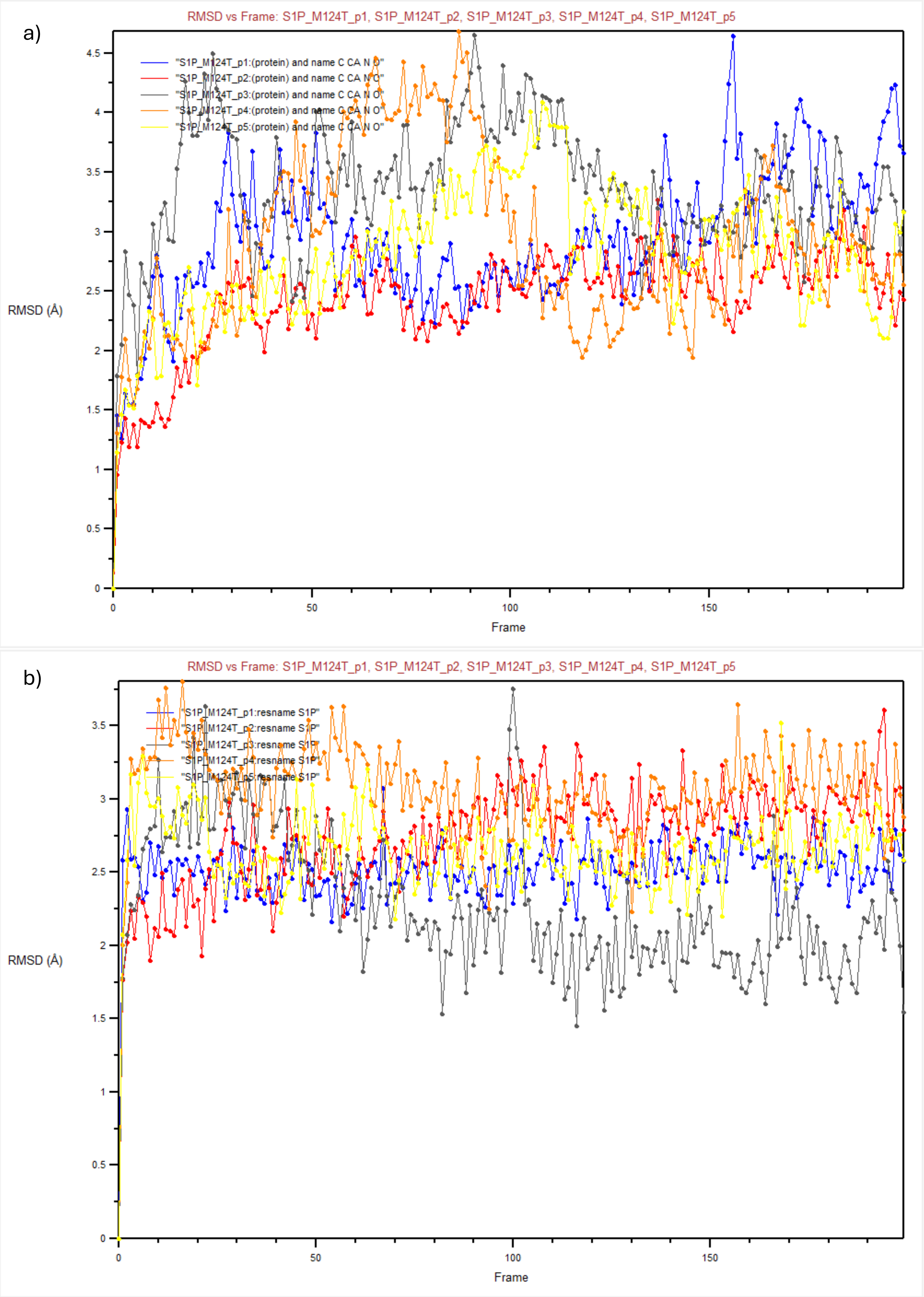

Figure S19 – RMSD of a) protein with M124$^{3.32}$T mutation and b) S1P in relation to the frames with five parallel simulations. A total of 200 frames coincided with 100 ns of simulation.

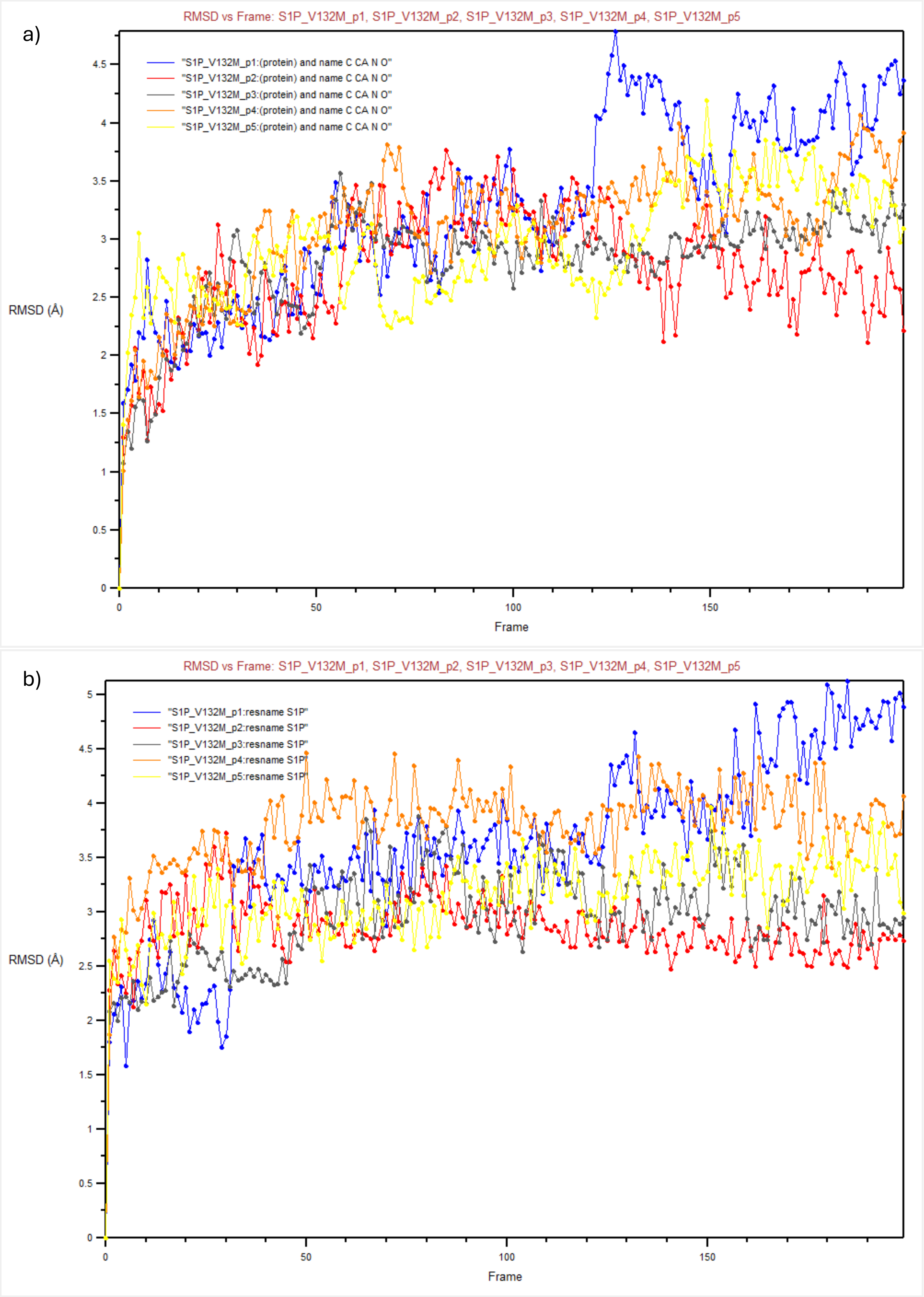

Figure S20 – RMSD of a) protein with V132$^{3.40}$M mutation and b) S1P in relation to the frames with five parallel simulations. A total of 200 frames coincided with 100 ns of simulation.

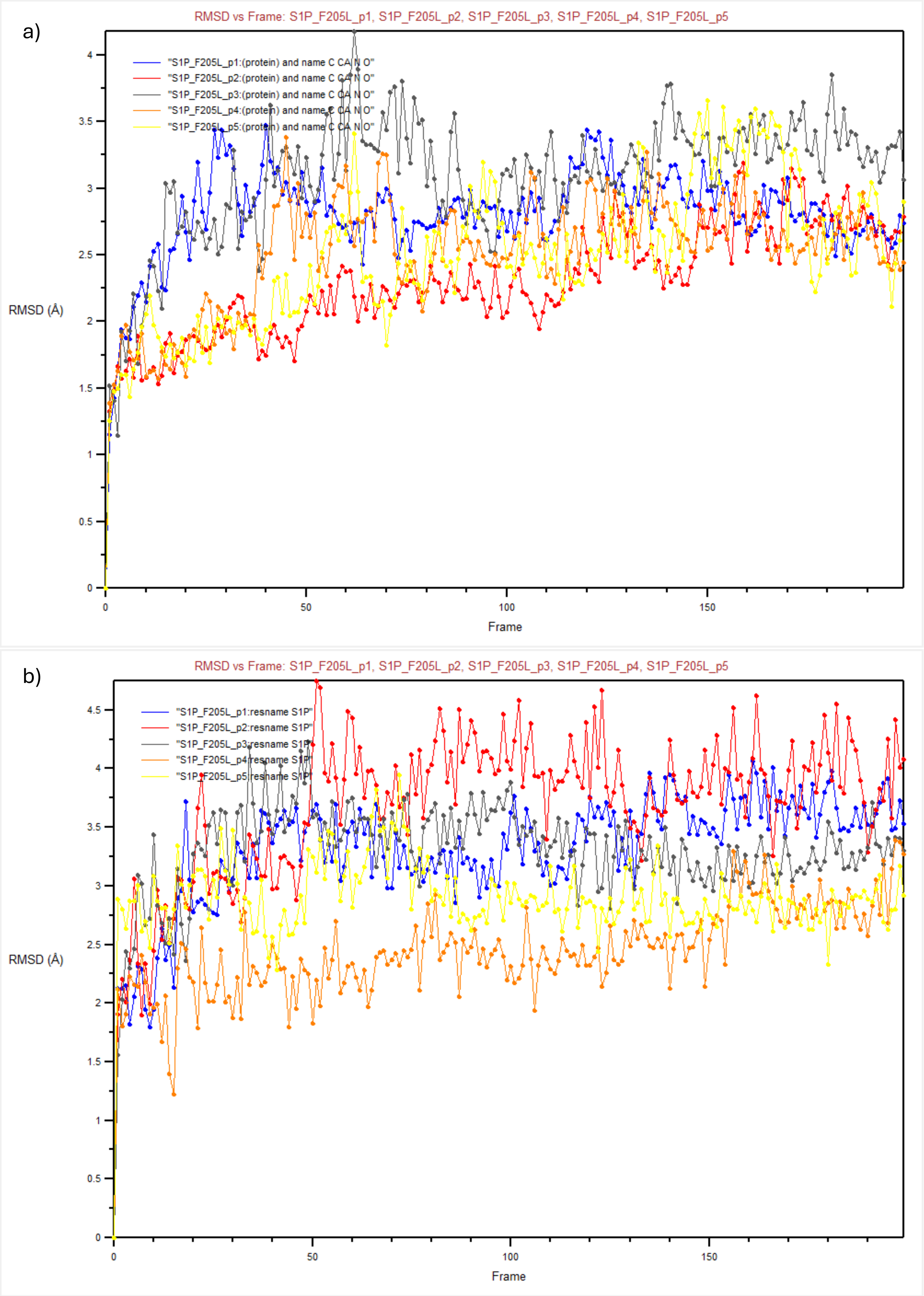

Figure S21 – RMSD of a) protein with F205$^{5.42}$L mutation and b) S1P in relation to the frames with five parallel simulations. A total of 200 frames coincided with 100 ns of simulation.

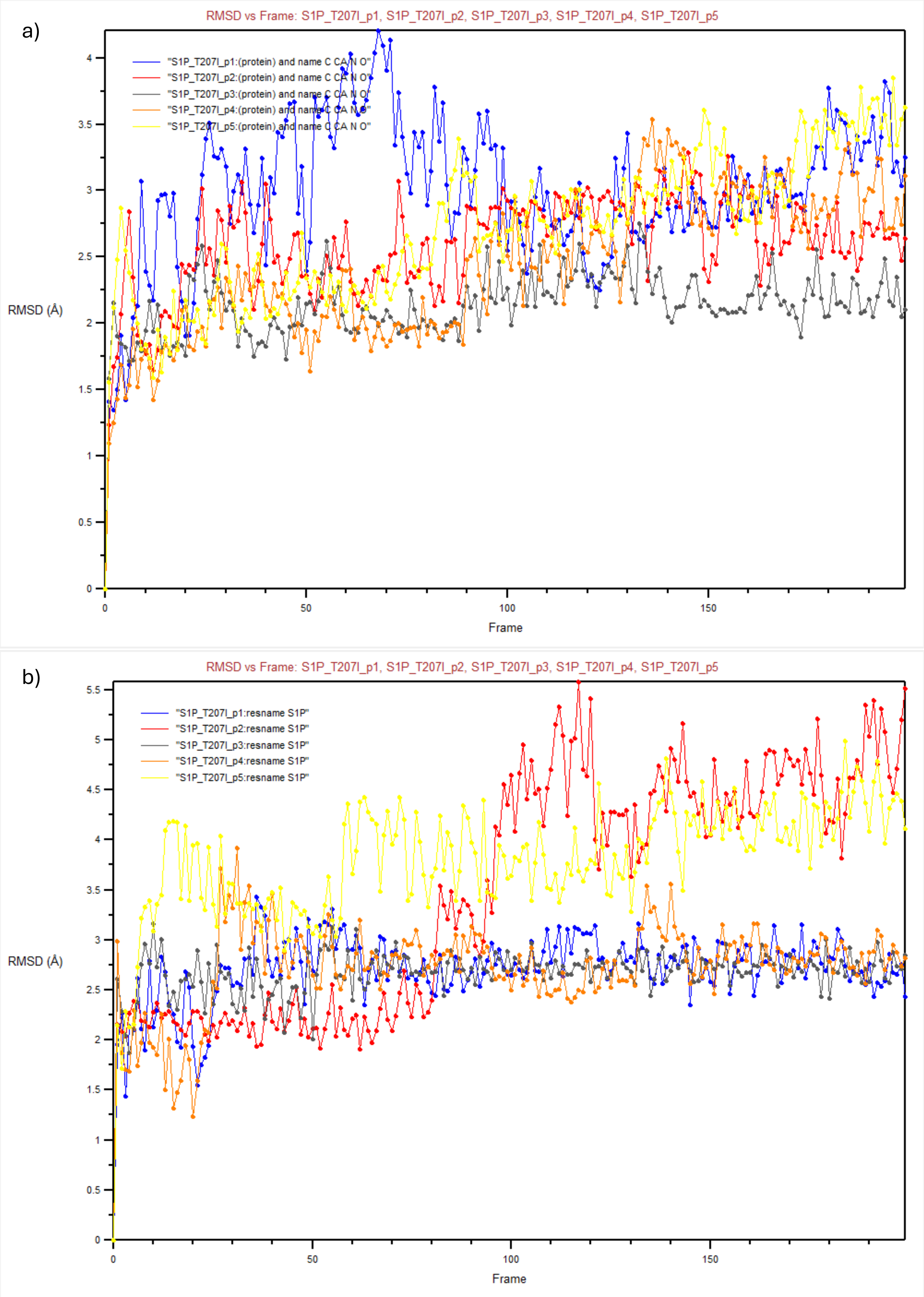

Figure S22 – RMSD of a) protein with T207$^{5.44}$I mutation and b) S1P in relation to the frames with five parallel simulations. A total of 200 frames coincided with 100 ns of simulation.

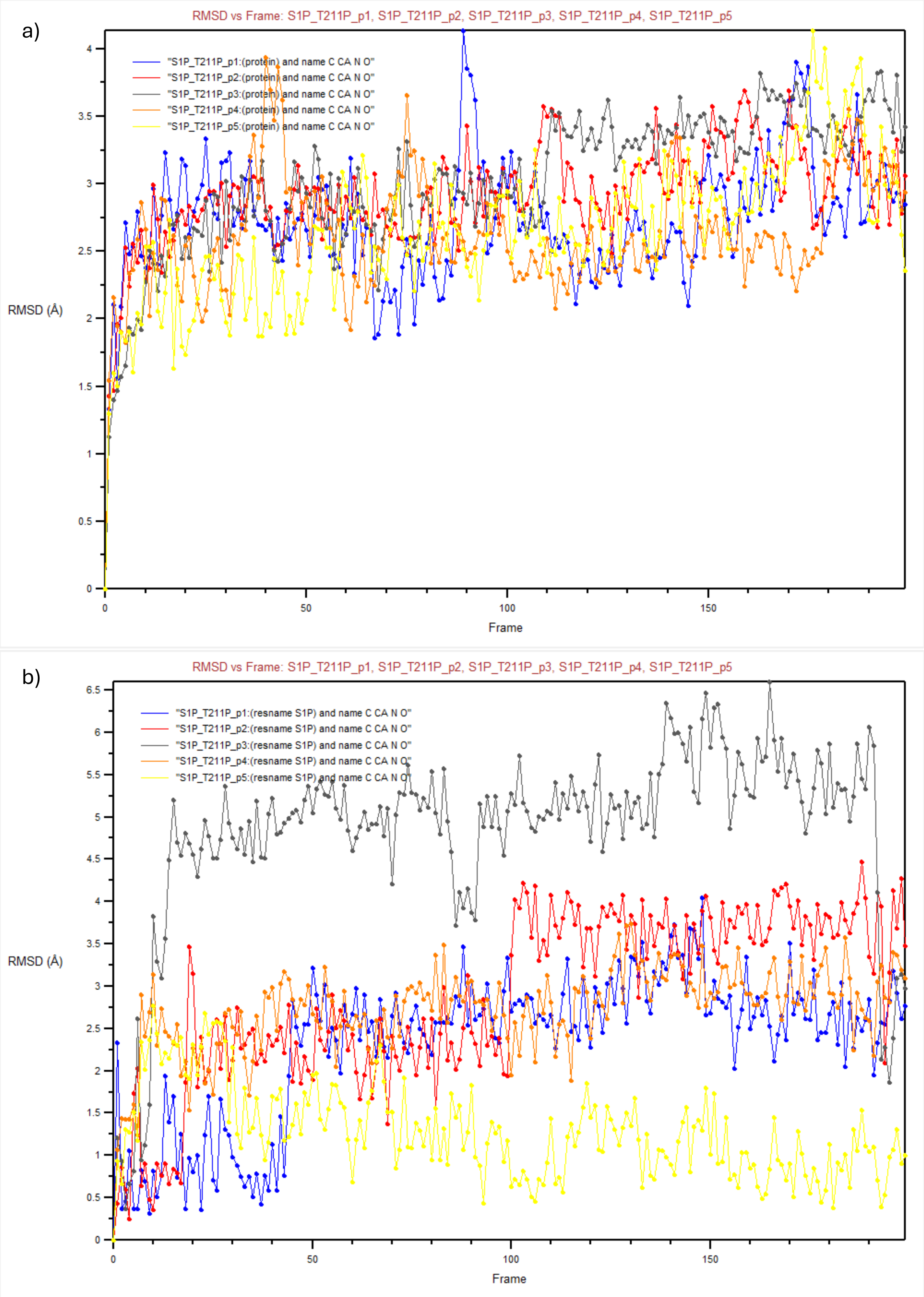

Figure S23 – RMSD of a) protein with T211$^{5.48}$P mutation and b) S1P in relation to the frames with five parallel simulations. A total of 200 frames coincided with 100 ns of simulation.

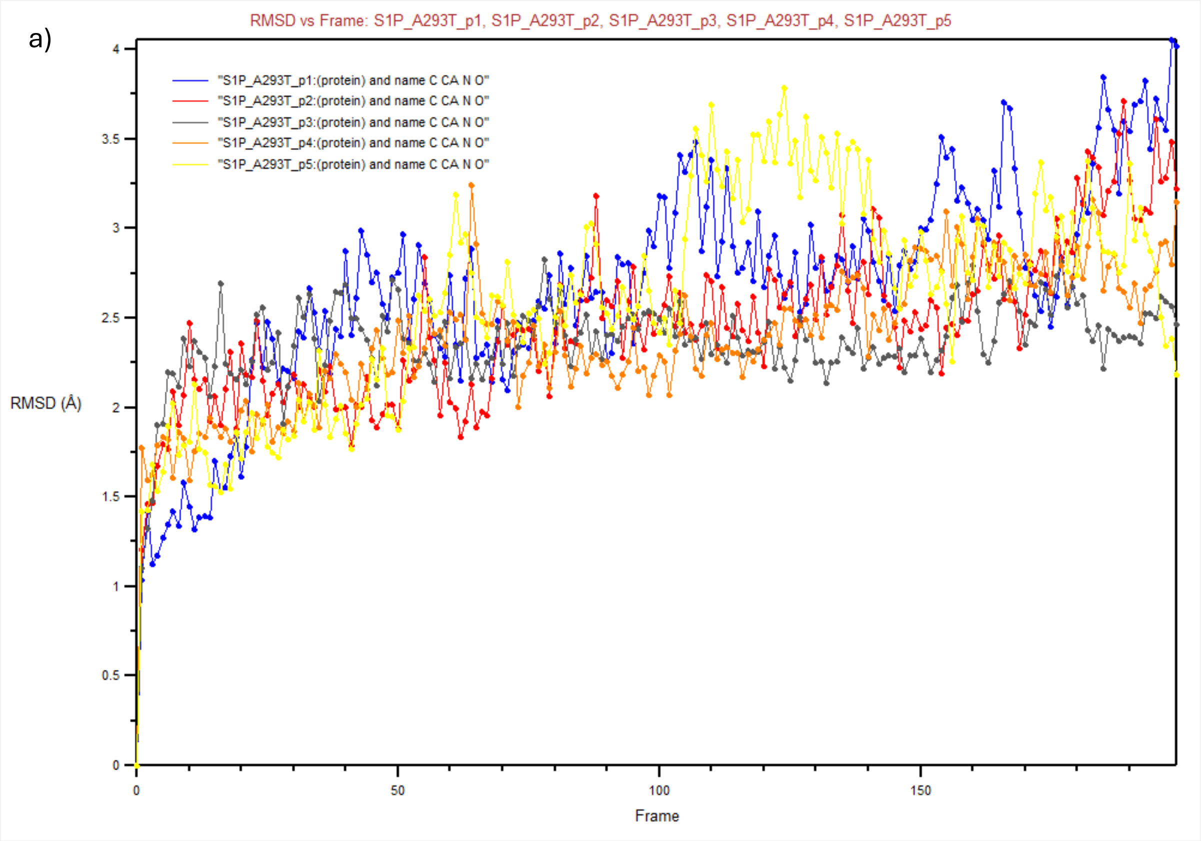

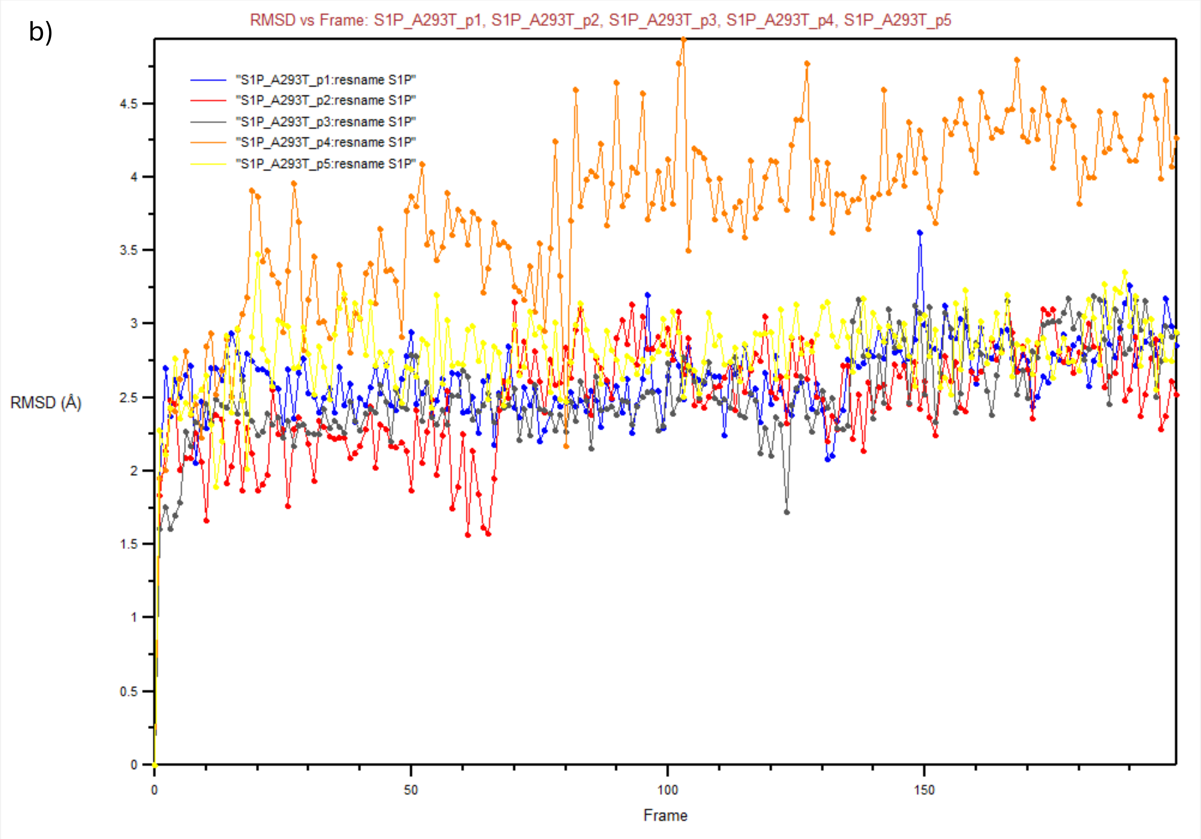

Figure S24 – RMSD of a) protein with A293$^{7.35}$T mutation and b) S1P in relation to the frames with five parallel simulations. A total of 200 frames coincided with 100 ns of simulation.

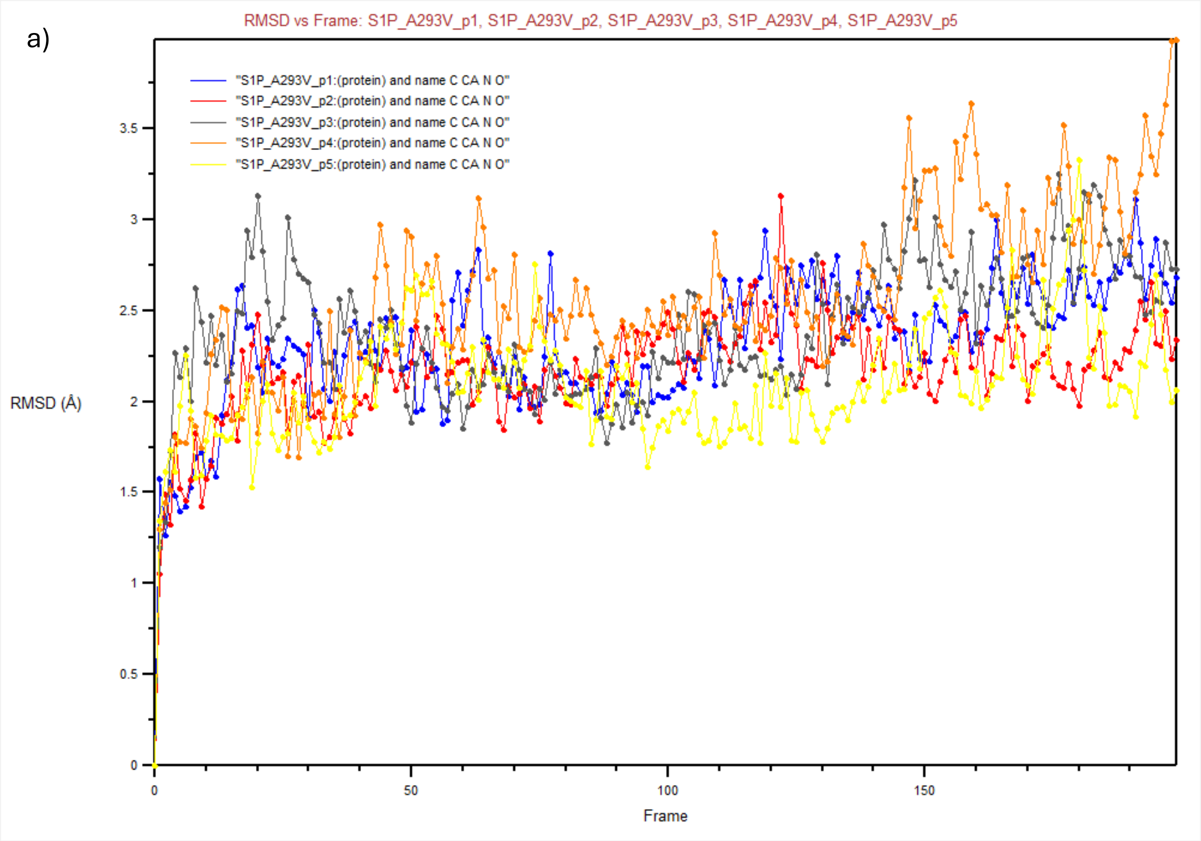

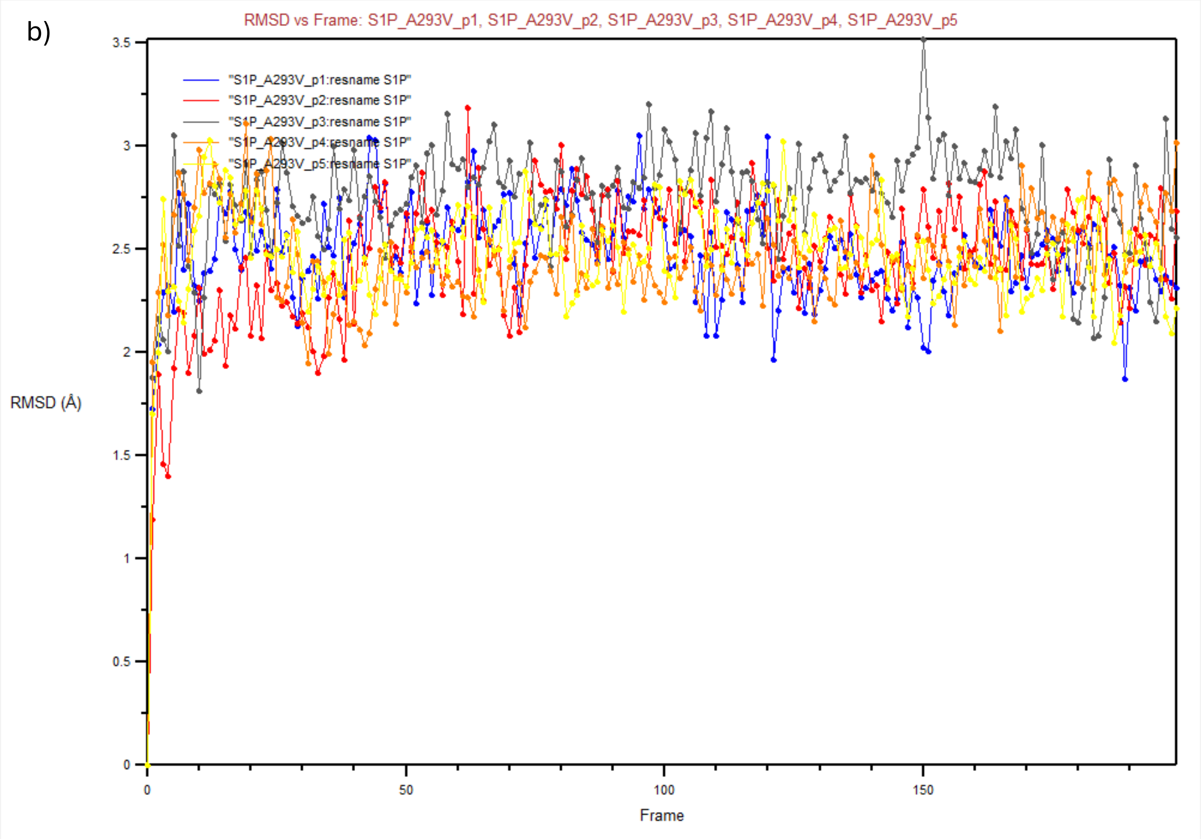

Figure S25 – RMSD of a) protein with A293$^{7.35}$V mutation and b) S1P in relation to the frames with five parallel simulations. A total of 200 frames coincided with 100 ns of simulation.

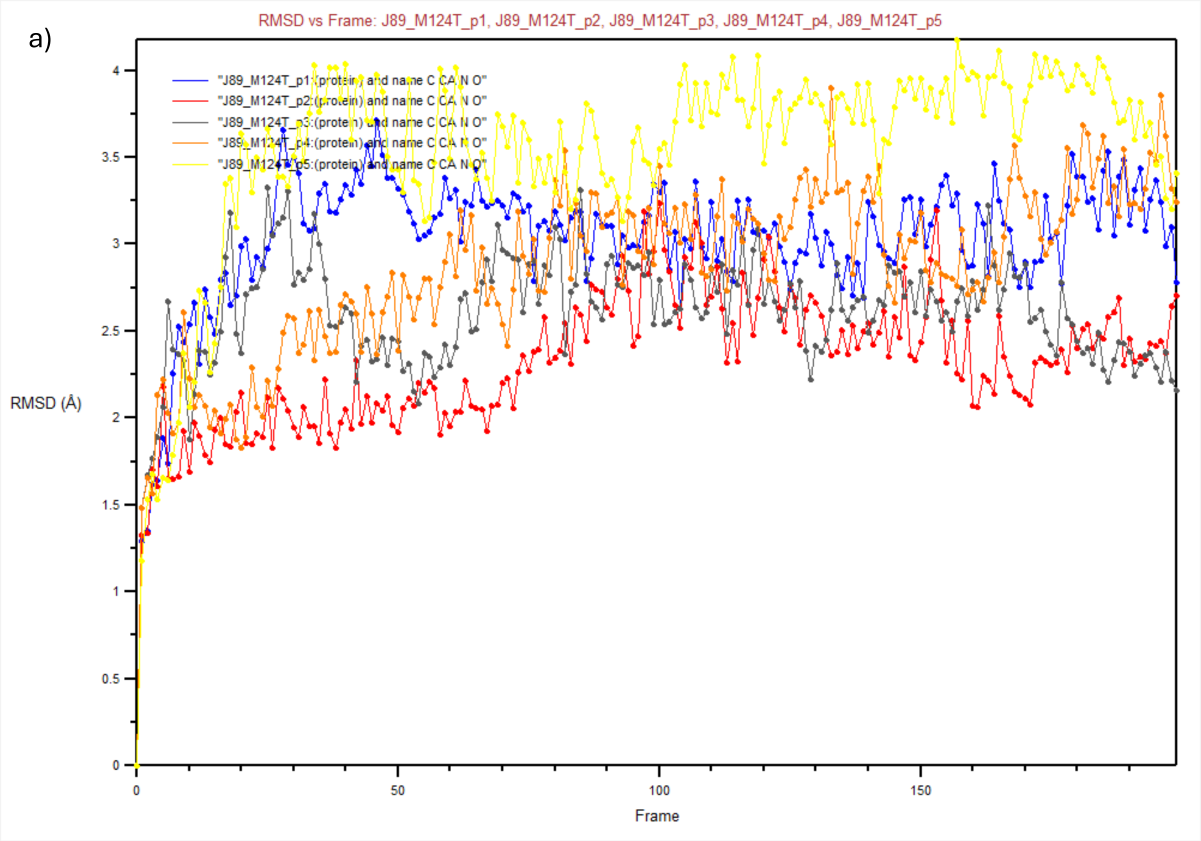

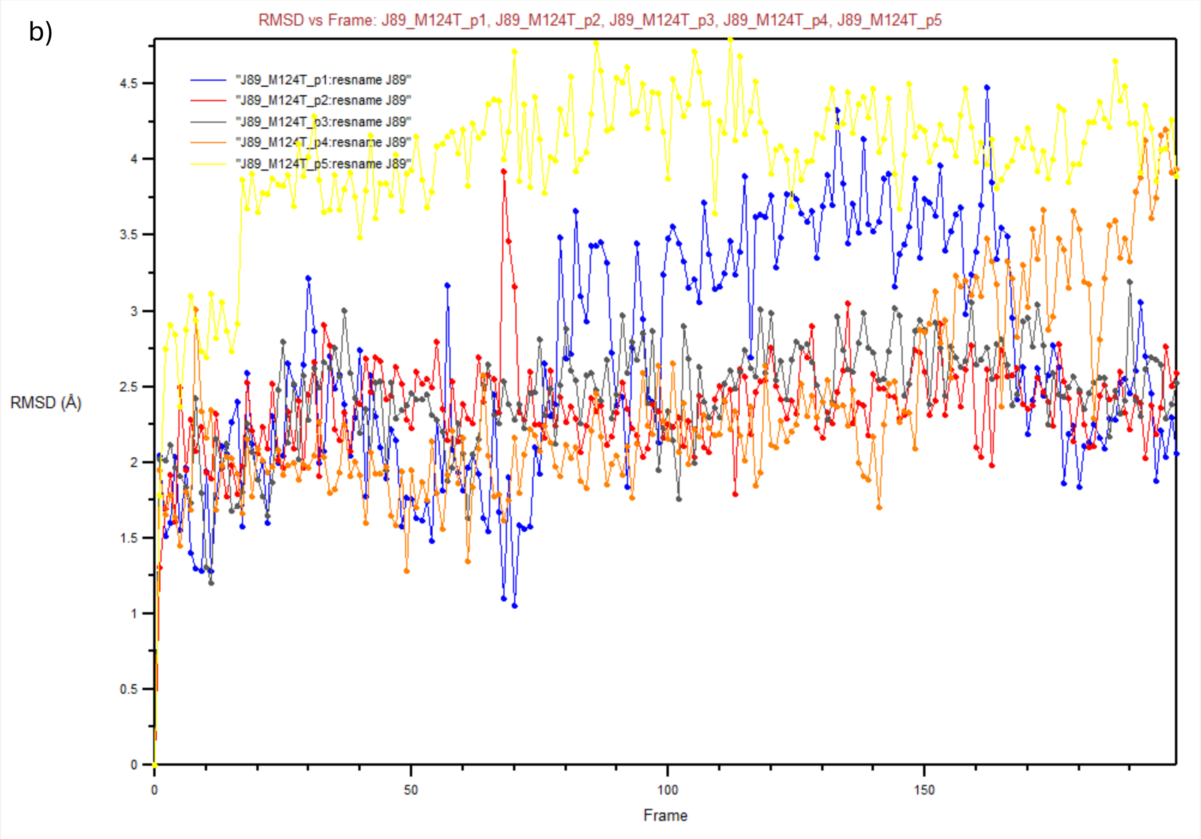

Figure S26 – RMSD of a) protein with M124$^{3.32}$T mutation and b) fingolimod in relation to the frames with five parallel simulations. A total of 200 frames coincided with 100 ns of simulation.

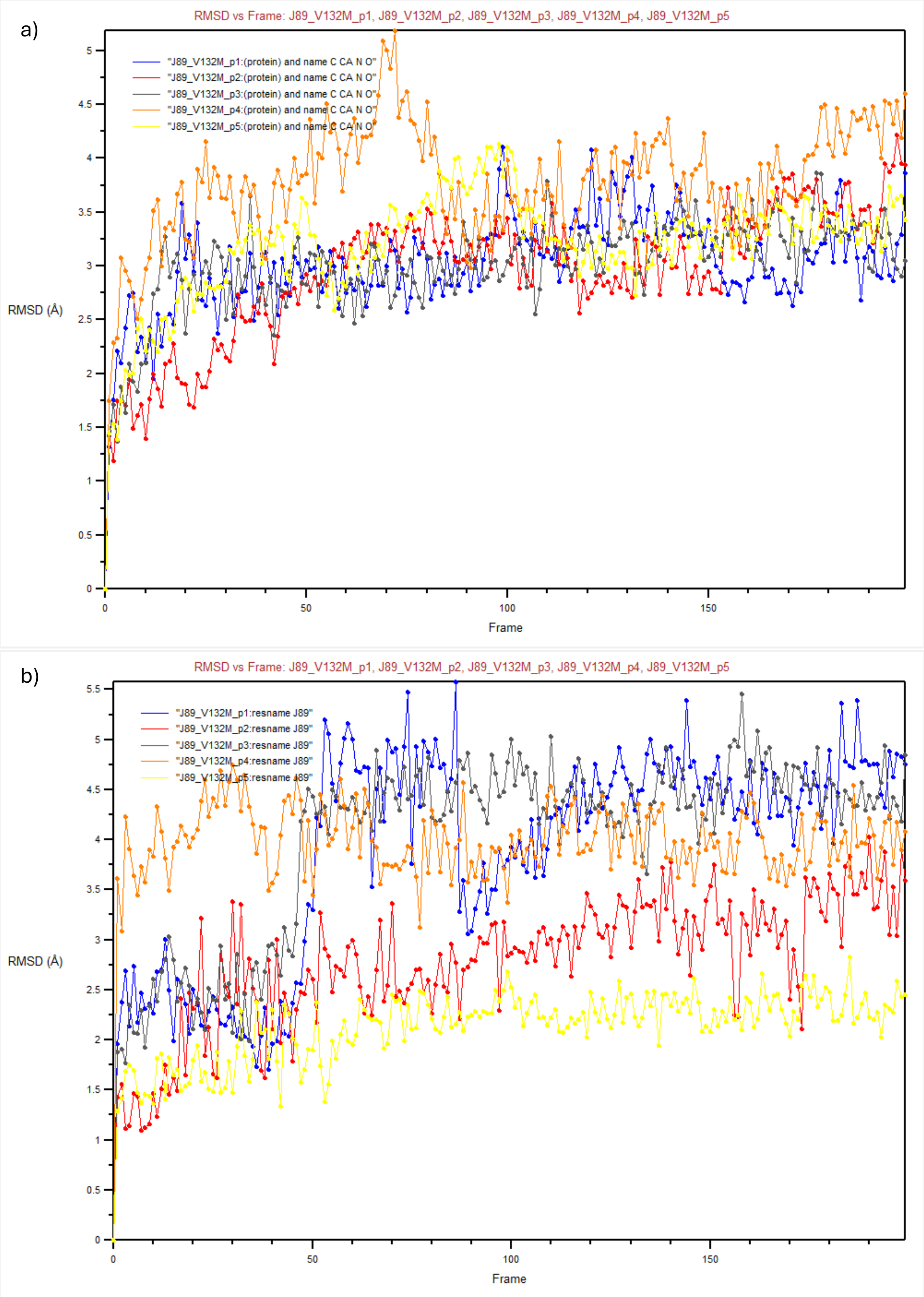

Figure S27 – RMSD of a) protein with V132$^{3.40}$M mutation and b) fingolimod in relation to the frames with five parallel simulations. A total of 200 frames coincided with 100 ns of simulation.

a)

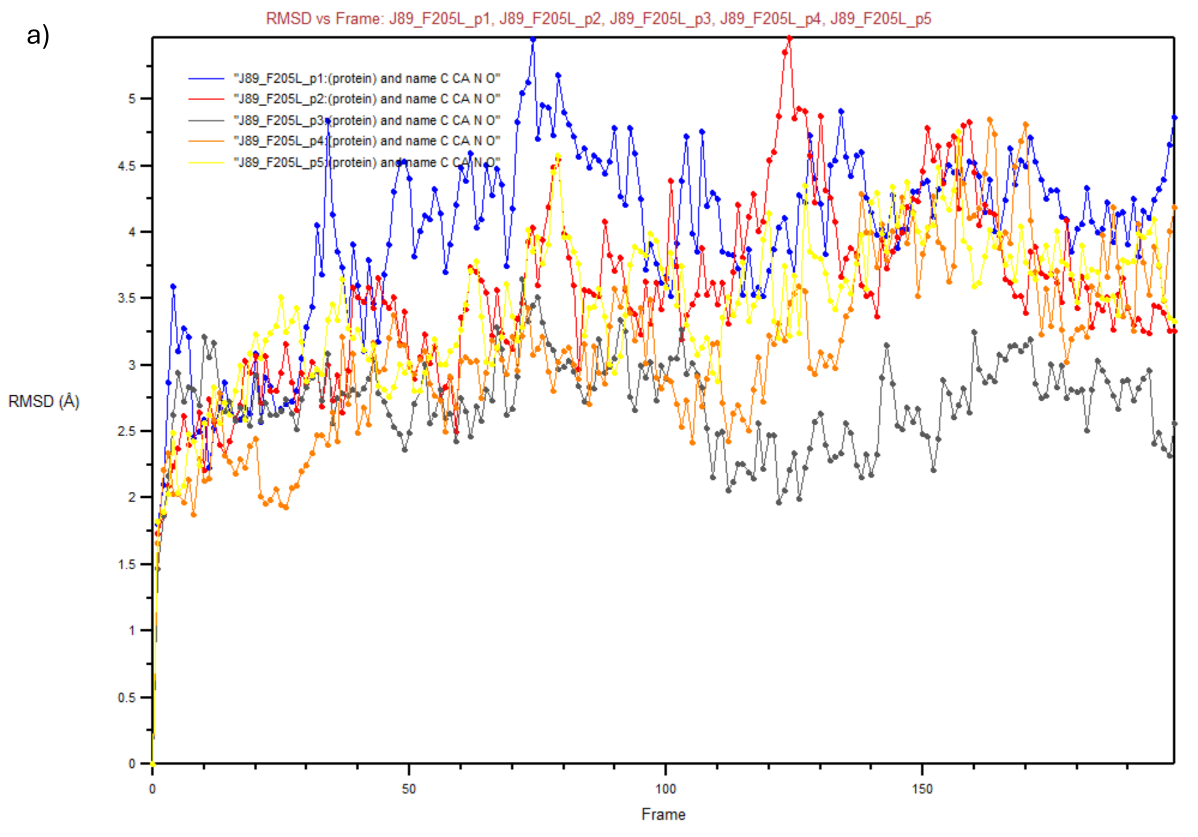

b)

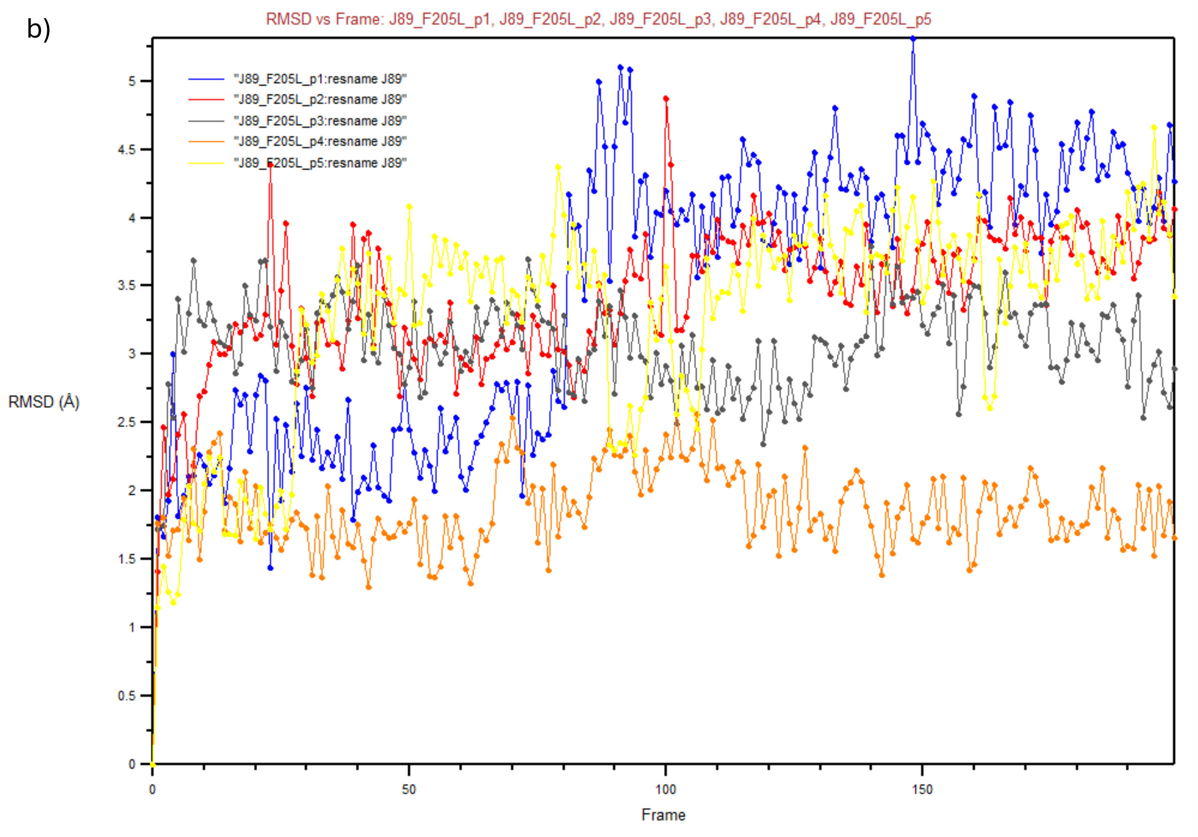

Figure S28 – RMSD of a) protein with F205$^{5.42}$L mutation and b) fingolimod in relation to the frames with five parallel simulations. A total of 200 frames coincided with 100 ns of simulation.

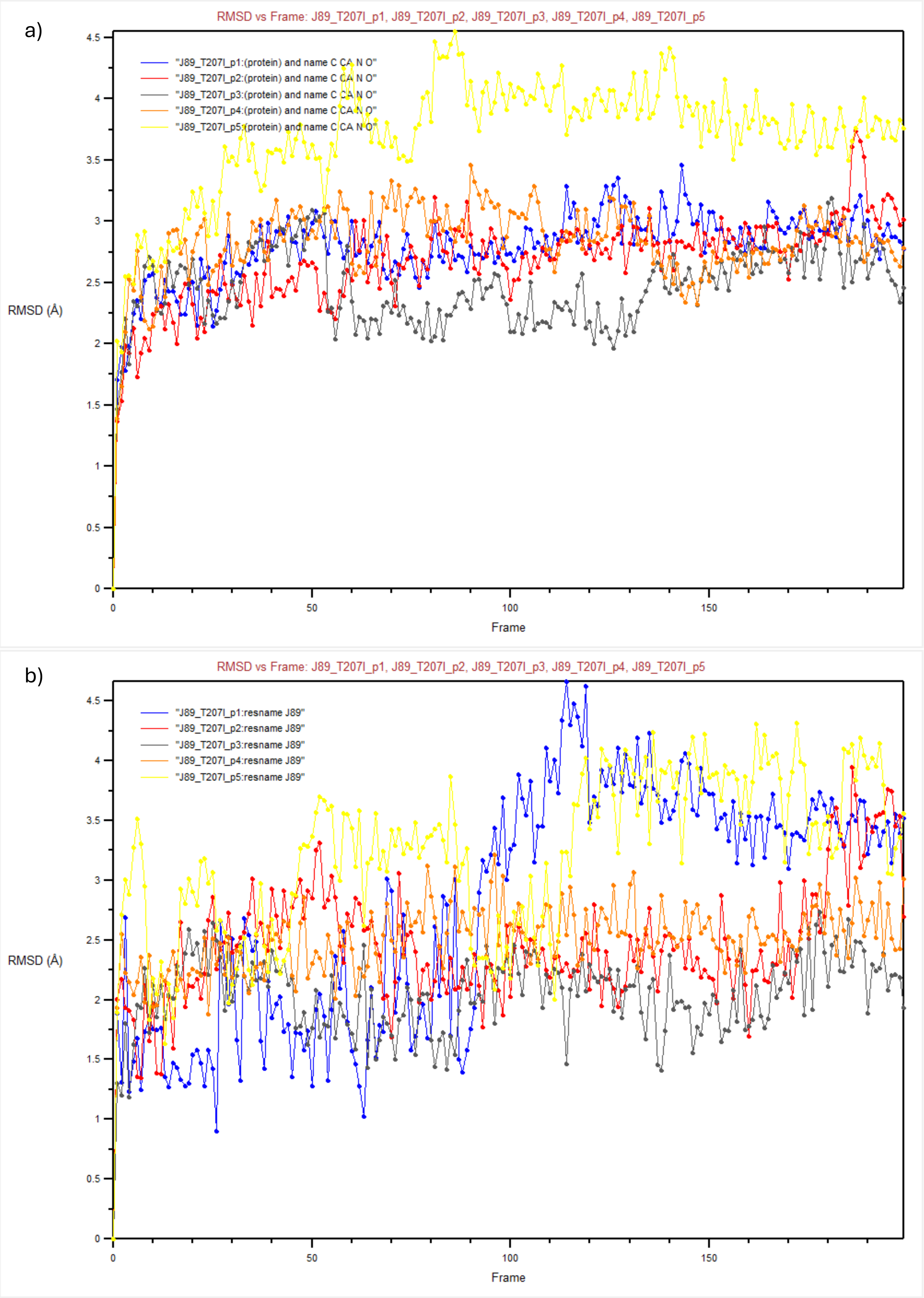

Figure S29 – RMSD of a) protein with T207$^{5.44}$I mutation and b) fingolimod in relation to the frames with five parallel simulations. A total of 200 frames coincided with 100 ns of simulation.

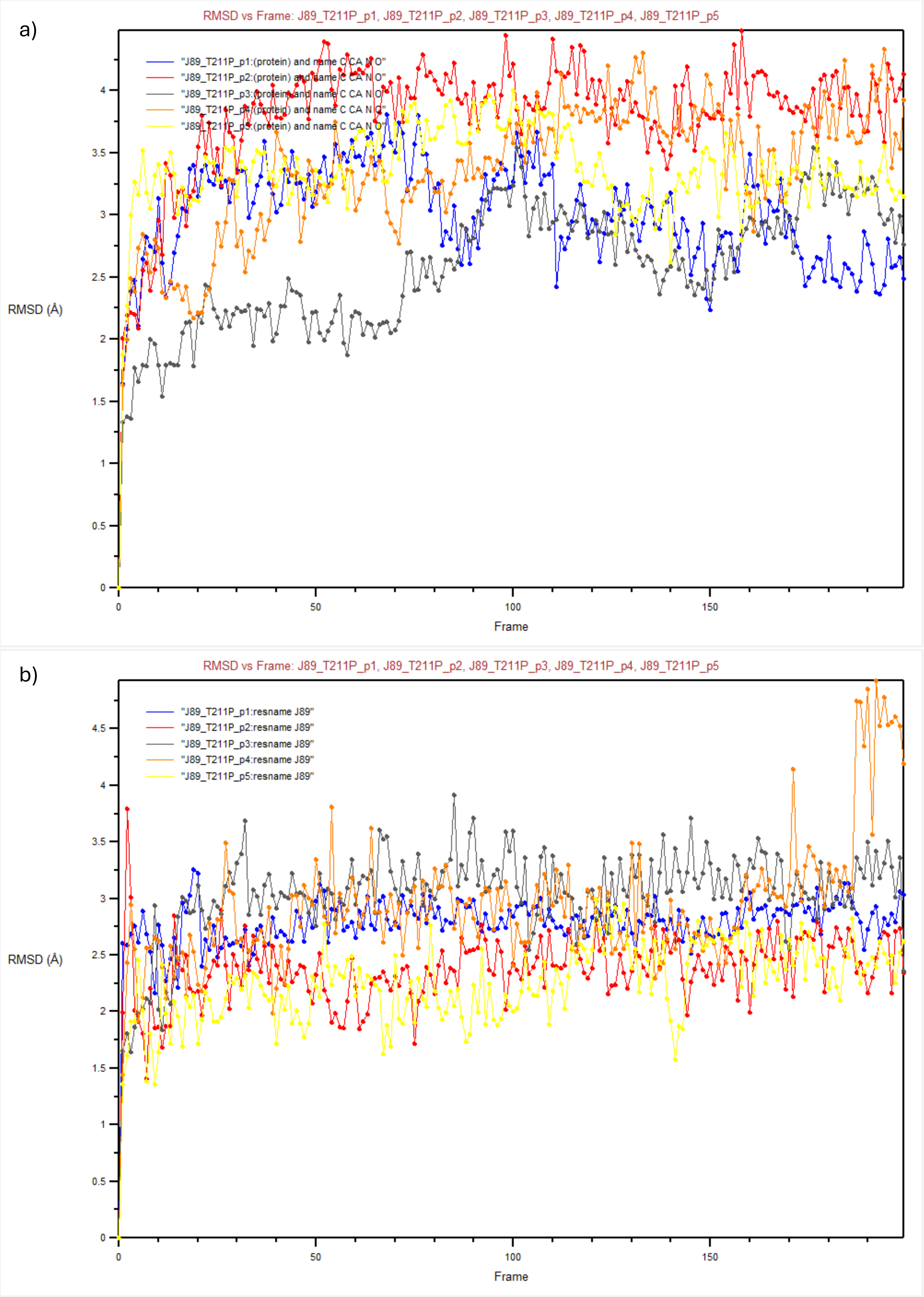

Figure S30 – RMSD of a) protein with T211$^{5.48}$P mutation and b) fingolimod in relation to the frames with five parallel simulations. A total of 200 frames coincided with 100 ns of simulation.

a)

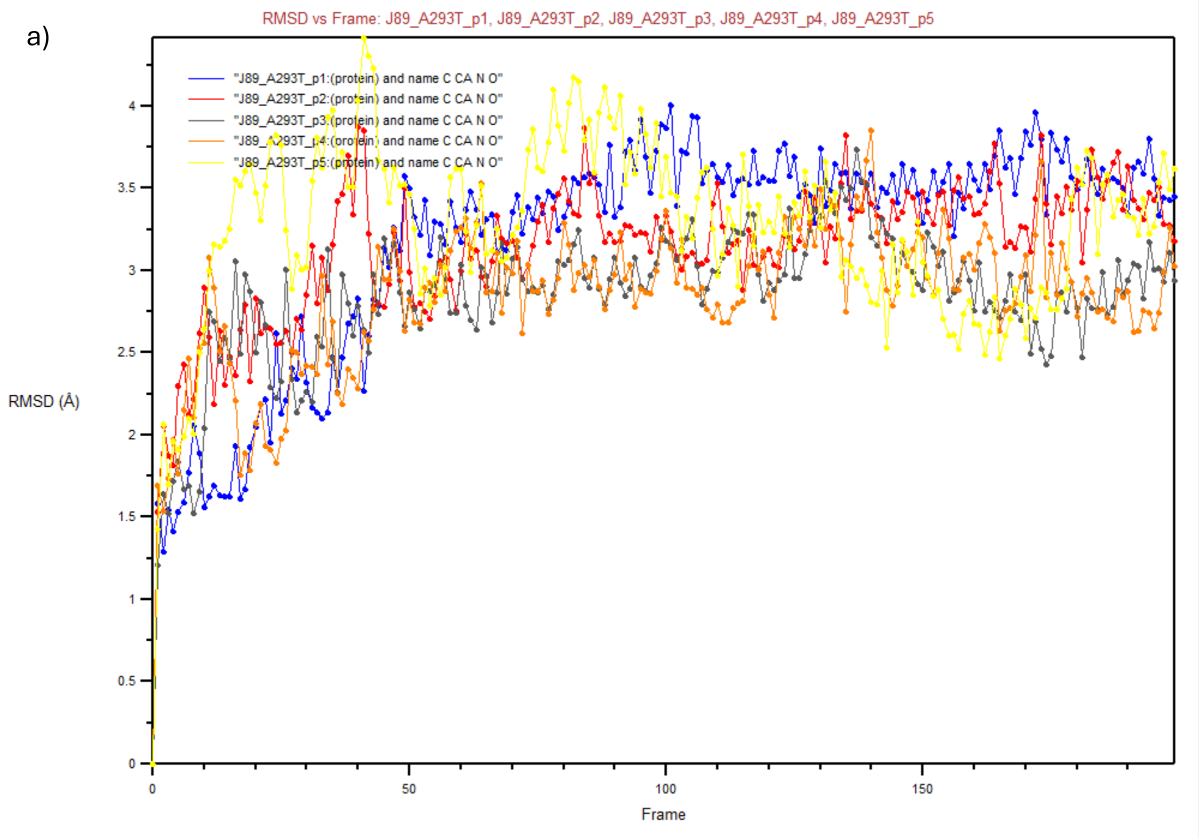

b)

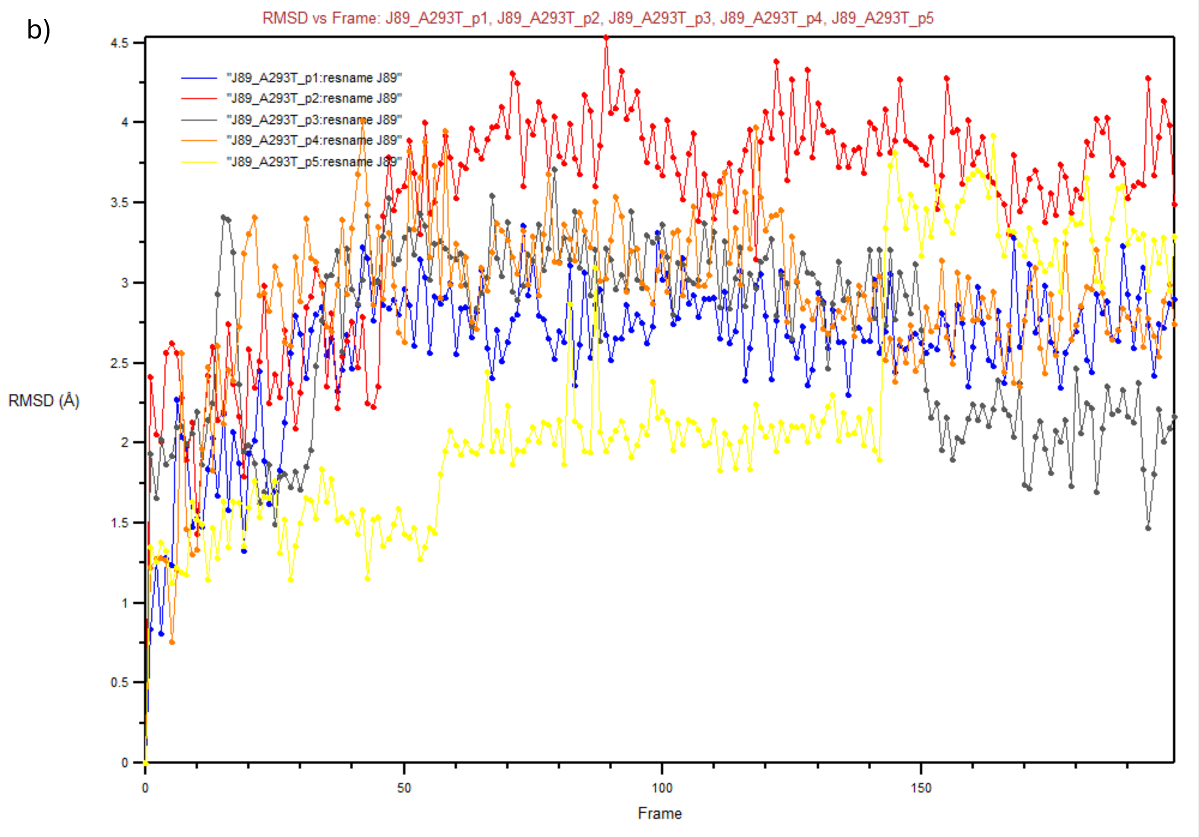

Figure S31 – RMSD of a) protein with A293$^{7.35}$T mutation and b) fingolimod in relation to the frames with five parallel simulations. A total of 200 frames coincided with 100 ns of simulation.

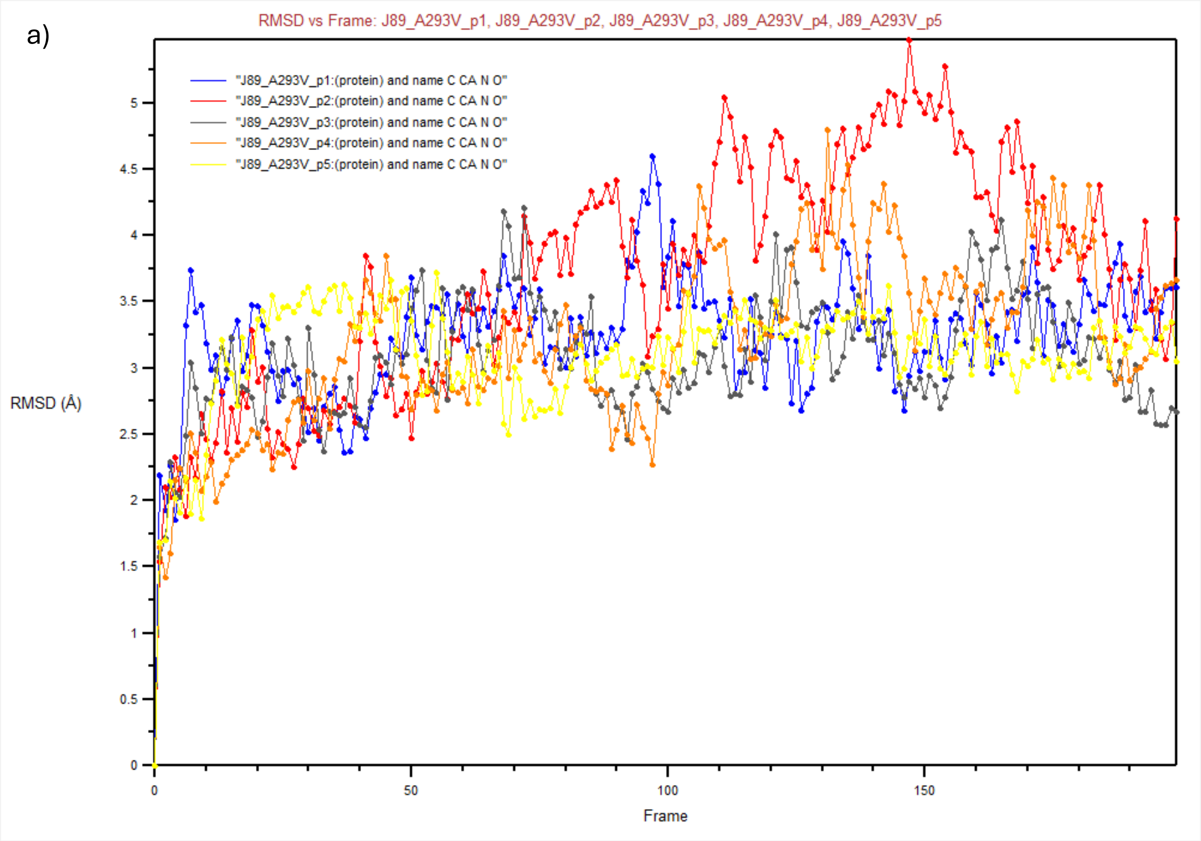

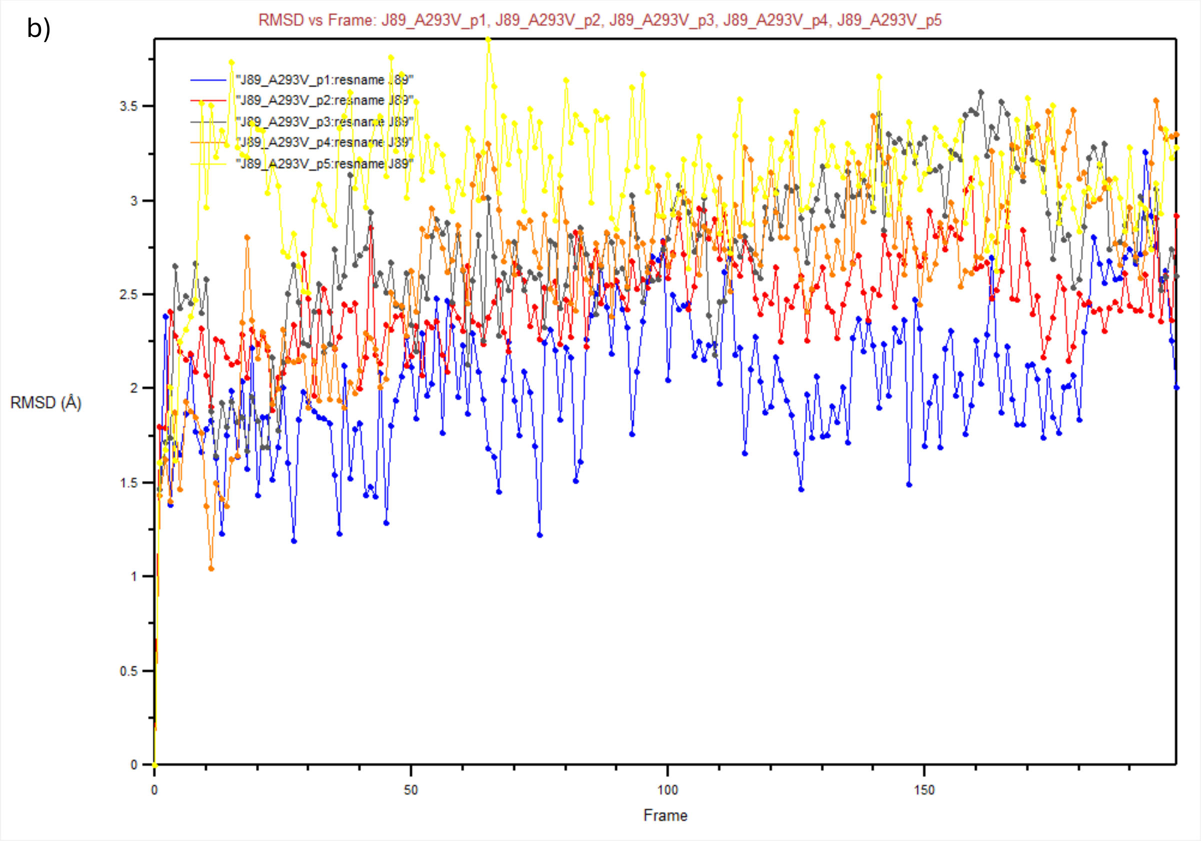

Figure S32 – RMSD of a) protein with A293$^{7.35}$V mutation and b) fingolimod in relation to the frames with five parallel simulations. A total of 200 frames coincided with 100 ns of simulation.

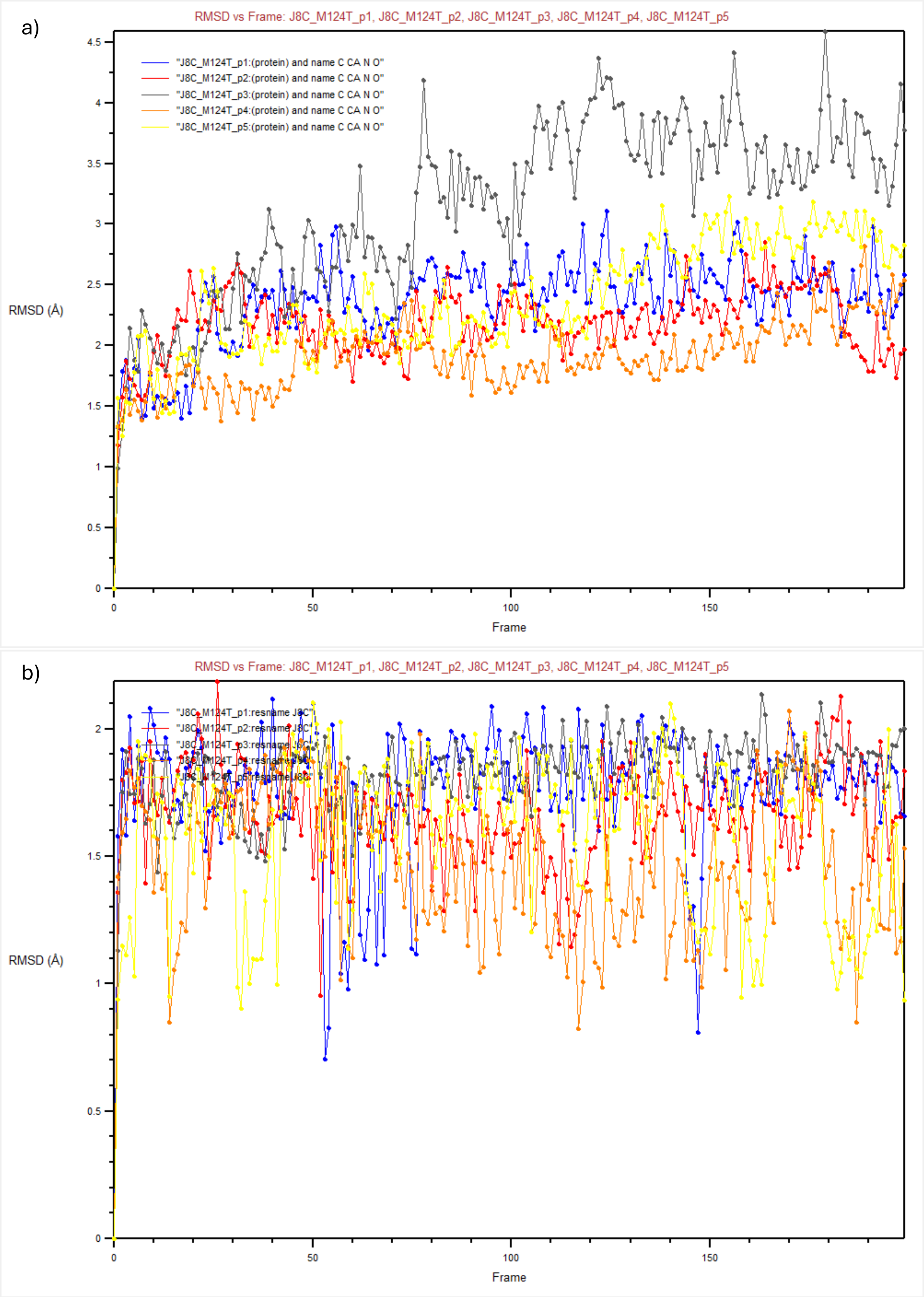

Figure S33 – RMSD of a) protein with $M124^{3.32}T$ mutation and b) siponimod in relation to the frames with five parallel simulations. A total of 200 frames coincided with 100 ns of simulation.

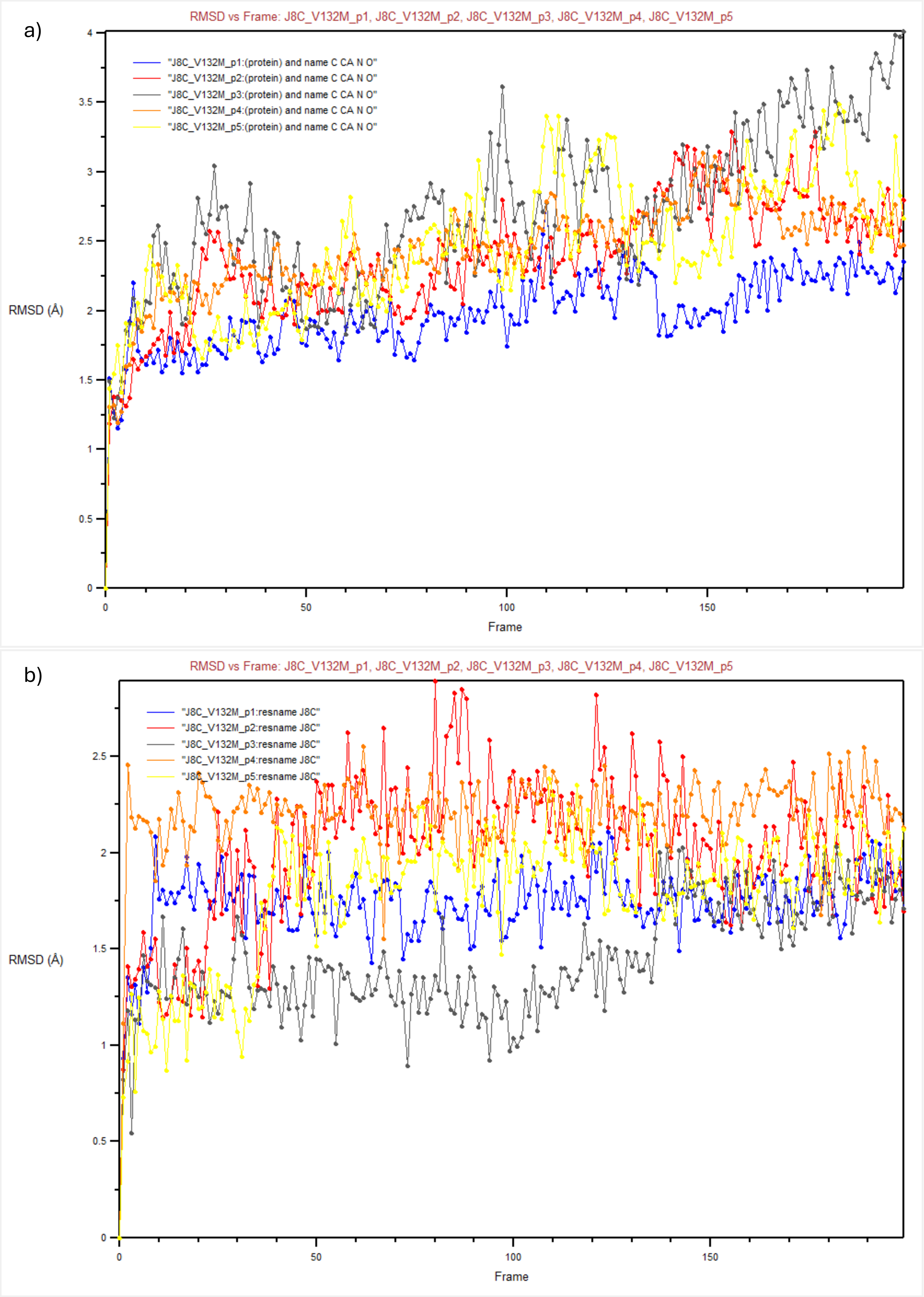

Figure S34 – RMSD of a) protein with V132$^{3.40}$M mutation and b) siponimod in relation to the frames with five parallel simulations. A total of 200 frames coincided with 100 ns of simulation.

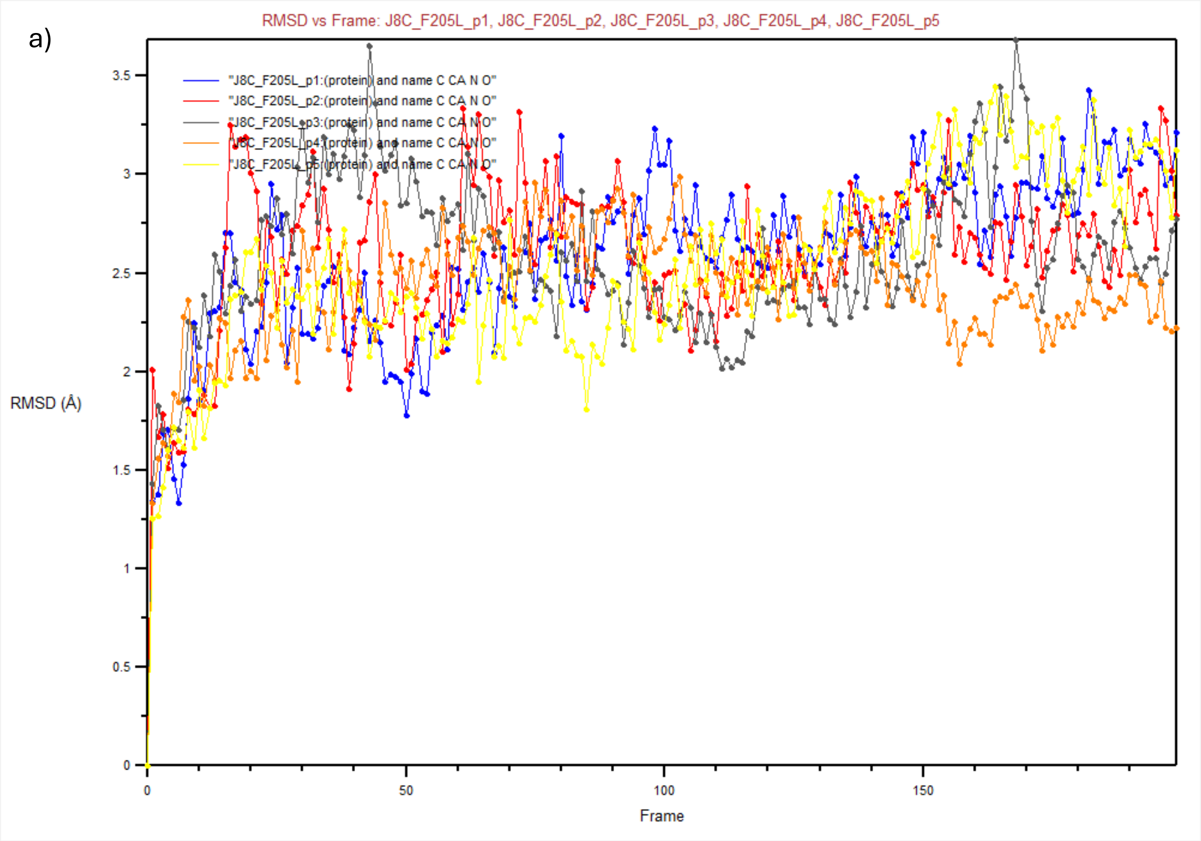

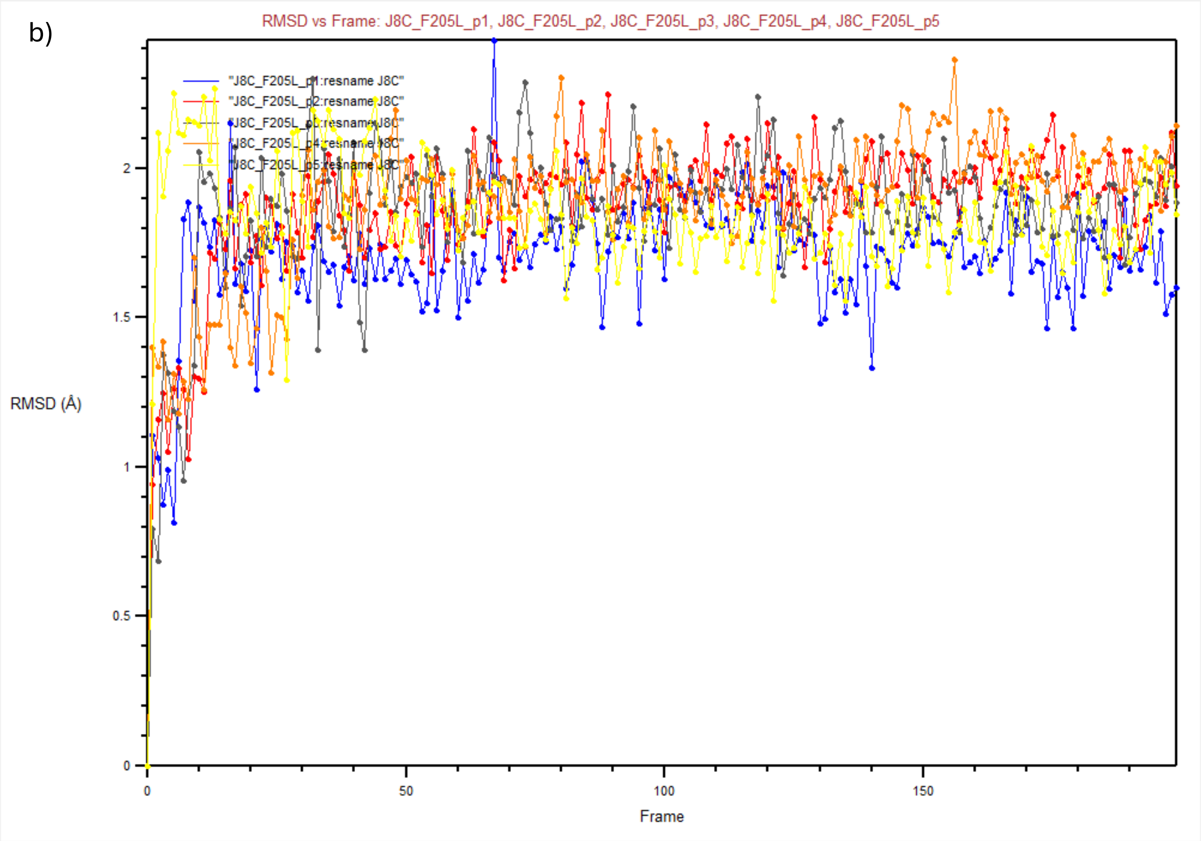

Figure S35 – RMSD of a) protein with F205$^{5.42}$L mutation and b) siponimod in relation to the frames with five parallel simulations. A total of 200 frames coincided with 100 ns of simulation.

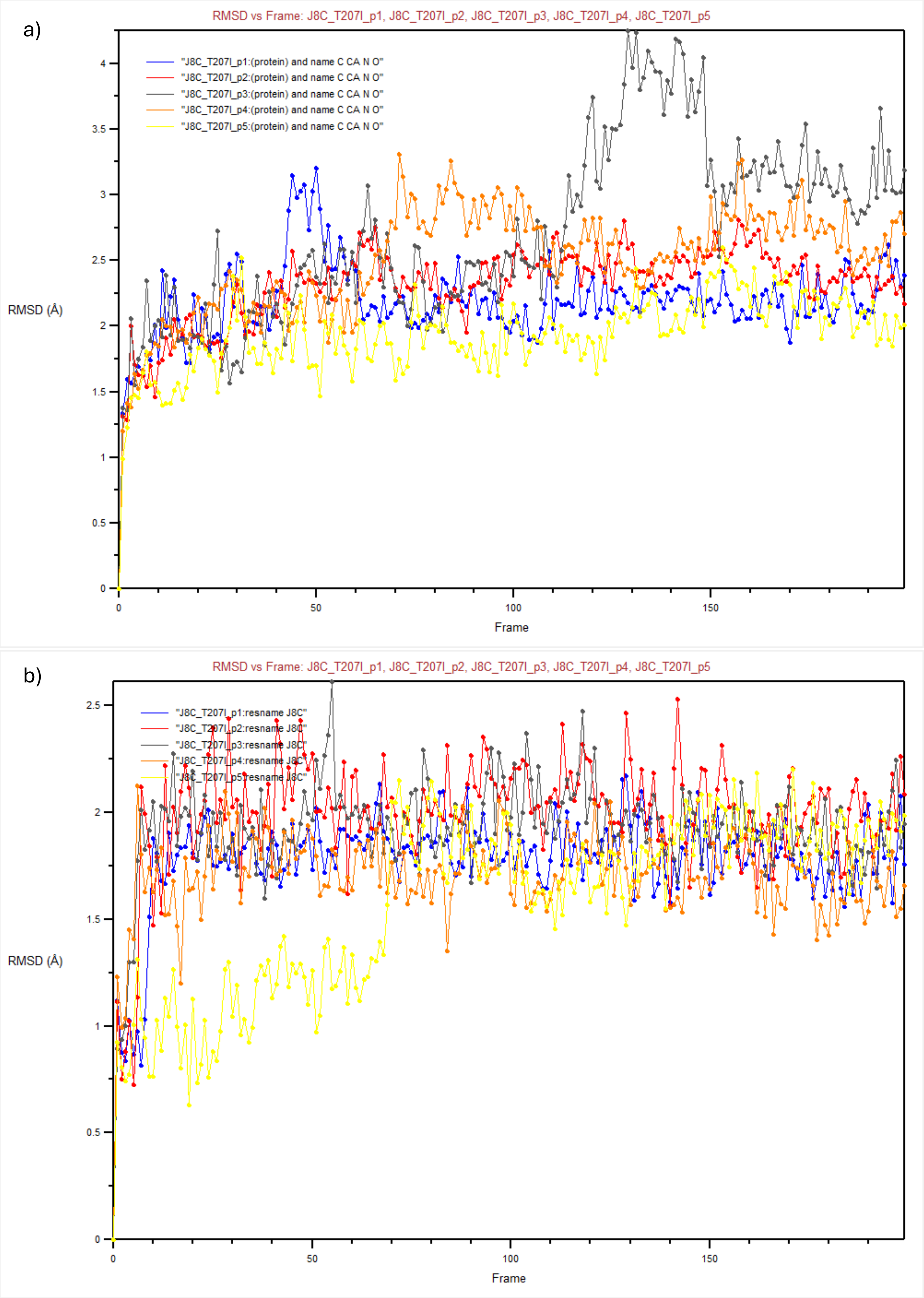

Figure S36 – RMSD of a) protein with T207$^{5.44}$I mutation and b) siponimod in relation to the frames with five parallel simulations. A total of 200 frames coincided with 100 ns of simulation.

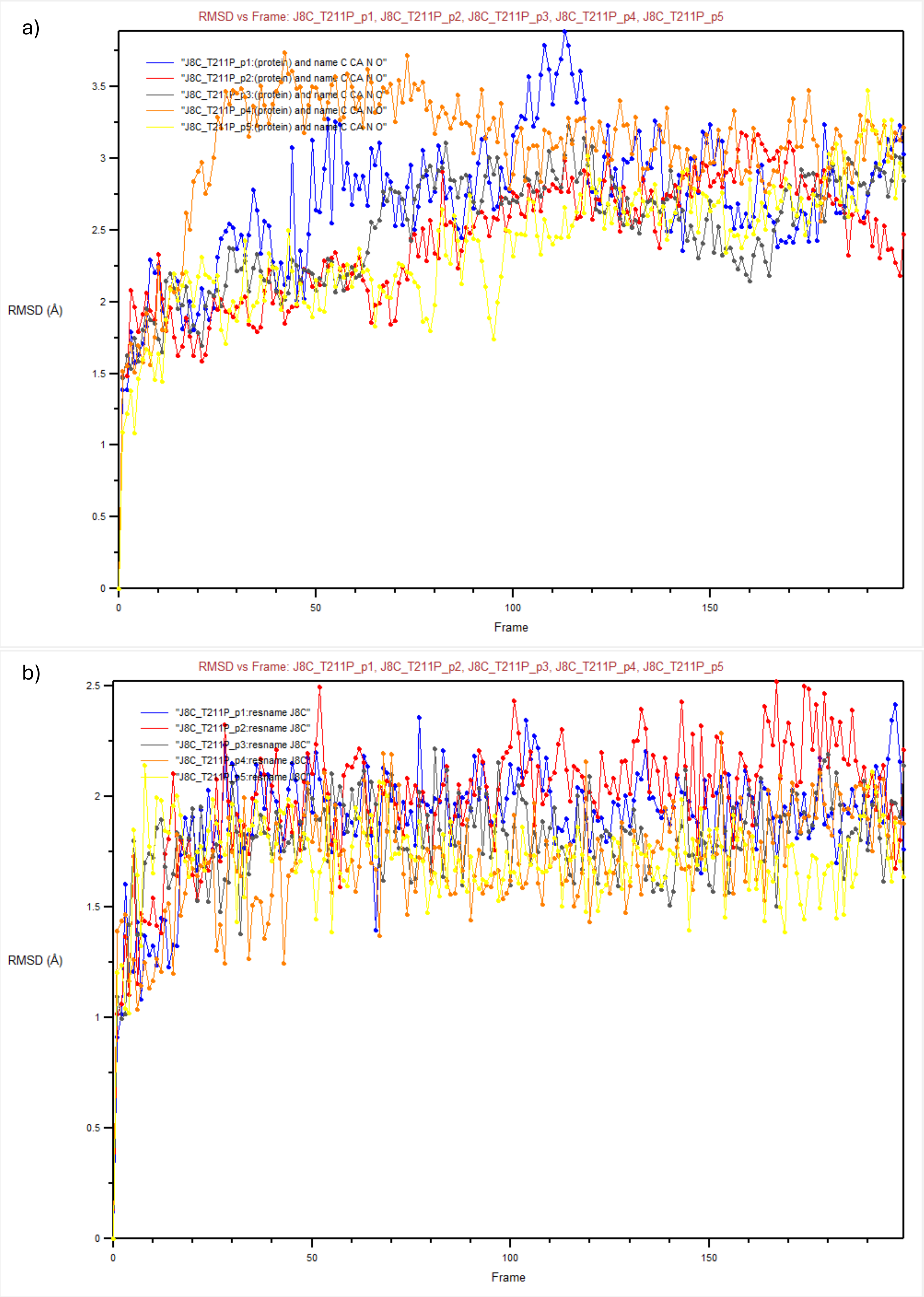

Figure S37 – RMSD of a) protein with T211$^{5.48}$P mutation and b) siponimod in relation to the frames with five parallel simulations. A total of 200 frames coincided with 100 ns of simulation.

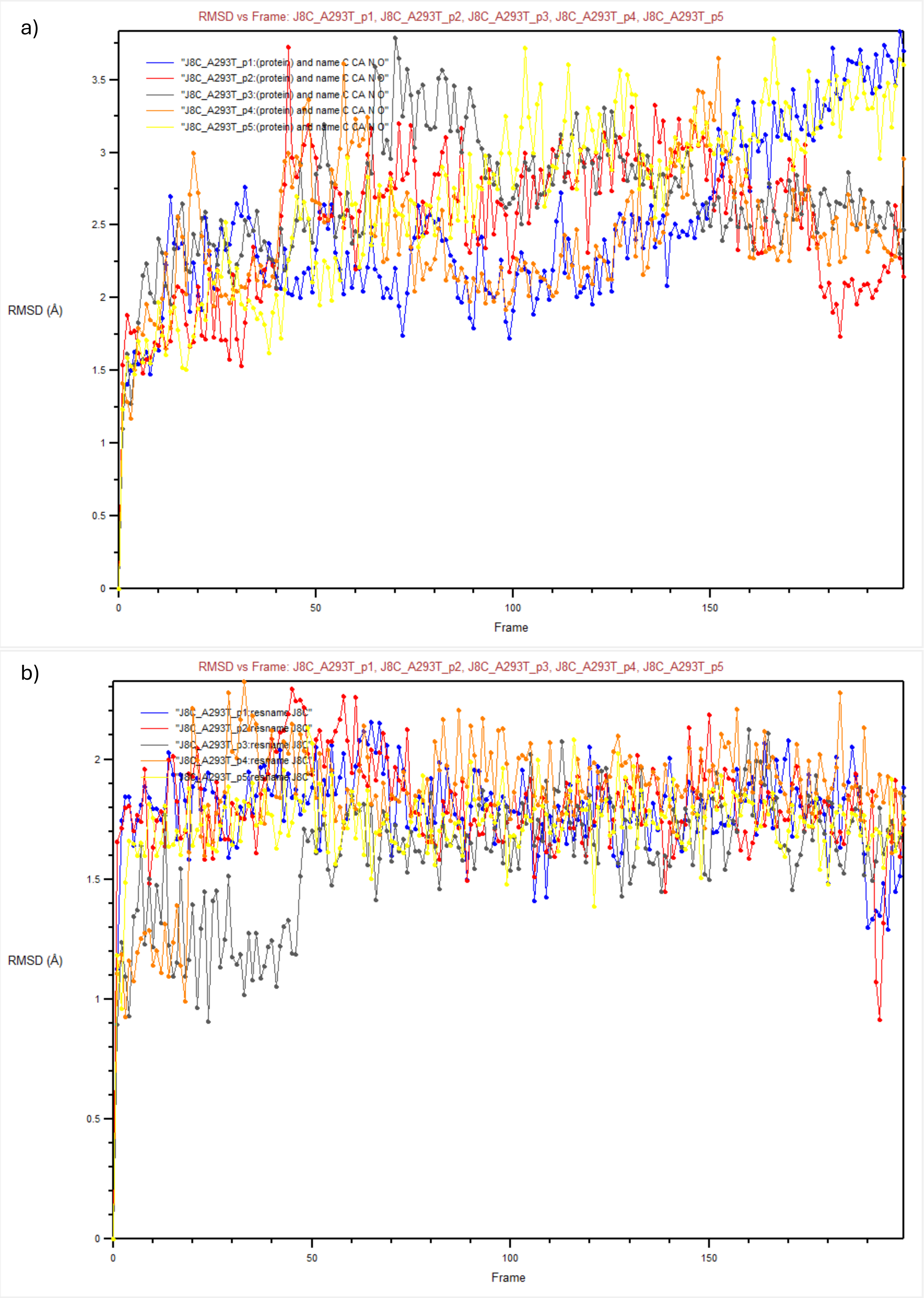

Figure S38 – RMSD of a) protein with A293$^{7.35}$T mutation and b) siponimod in relation to the frames with five parallel simulations. A total of 200 frames coincided with 100 ns of simulation.

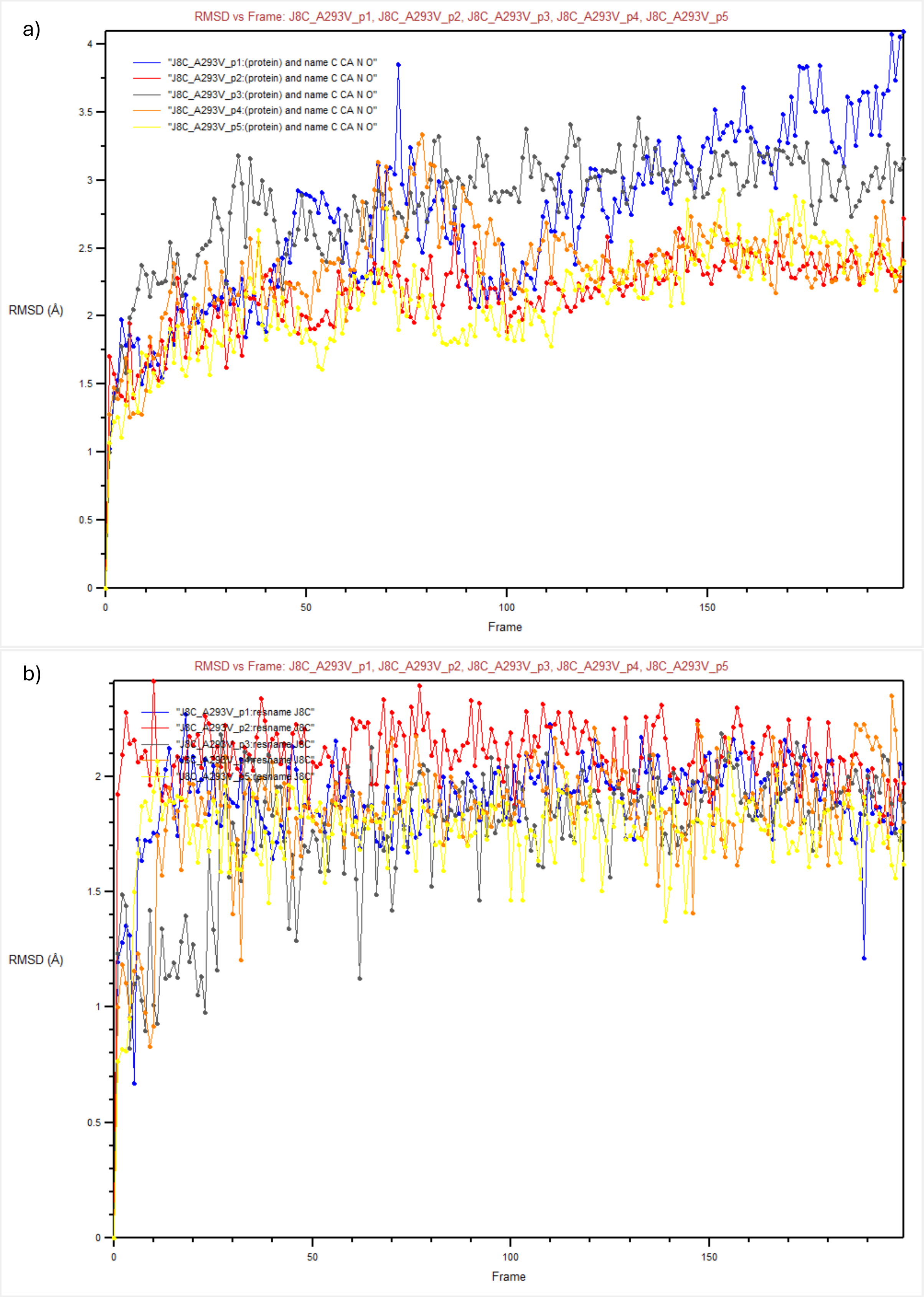

Figure S39 – RMSD of a) protein with A293$^{7.35}$V mutation and b) siponimod in relation to the frames with five parallel simulations. A total of 200 frames coincided with 100 ns of simulation.

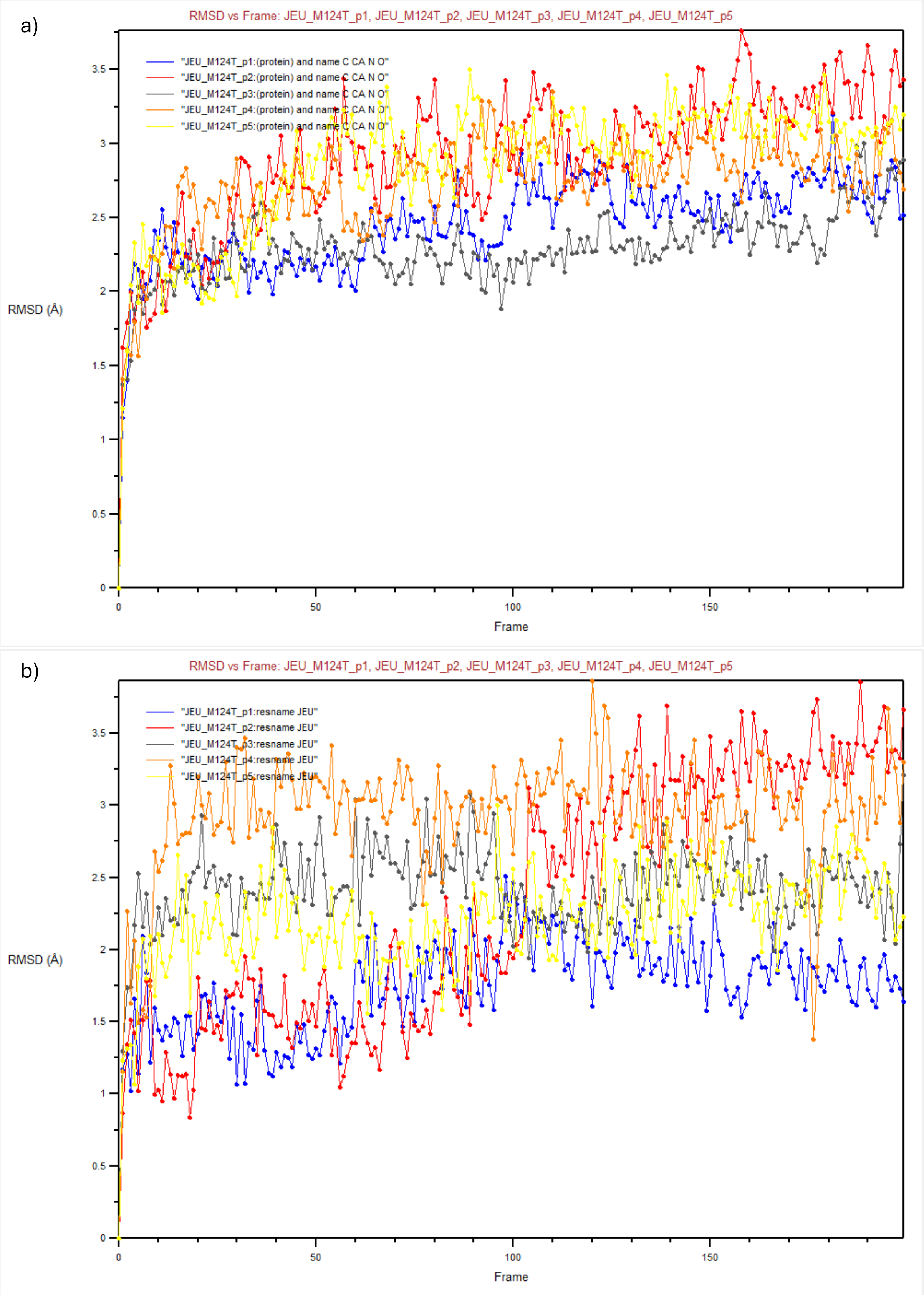

Figure S40 – RMSD of a) protein with M124$^{3.32}$T mutation and b) ozanimod in relation to the frames with five parallel simulations. A total of 200 frames coincided with 100 ns of simulation.

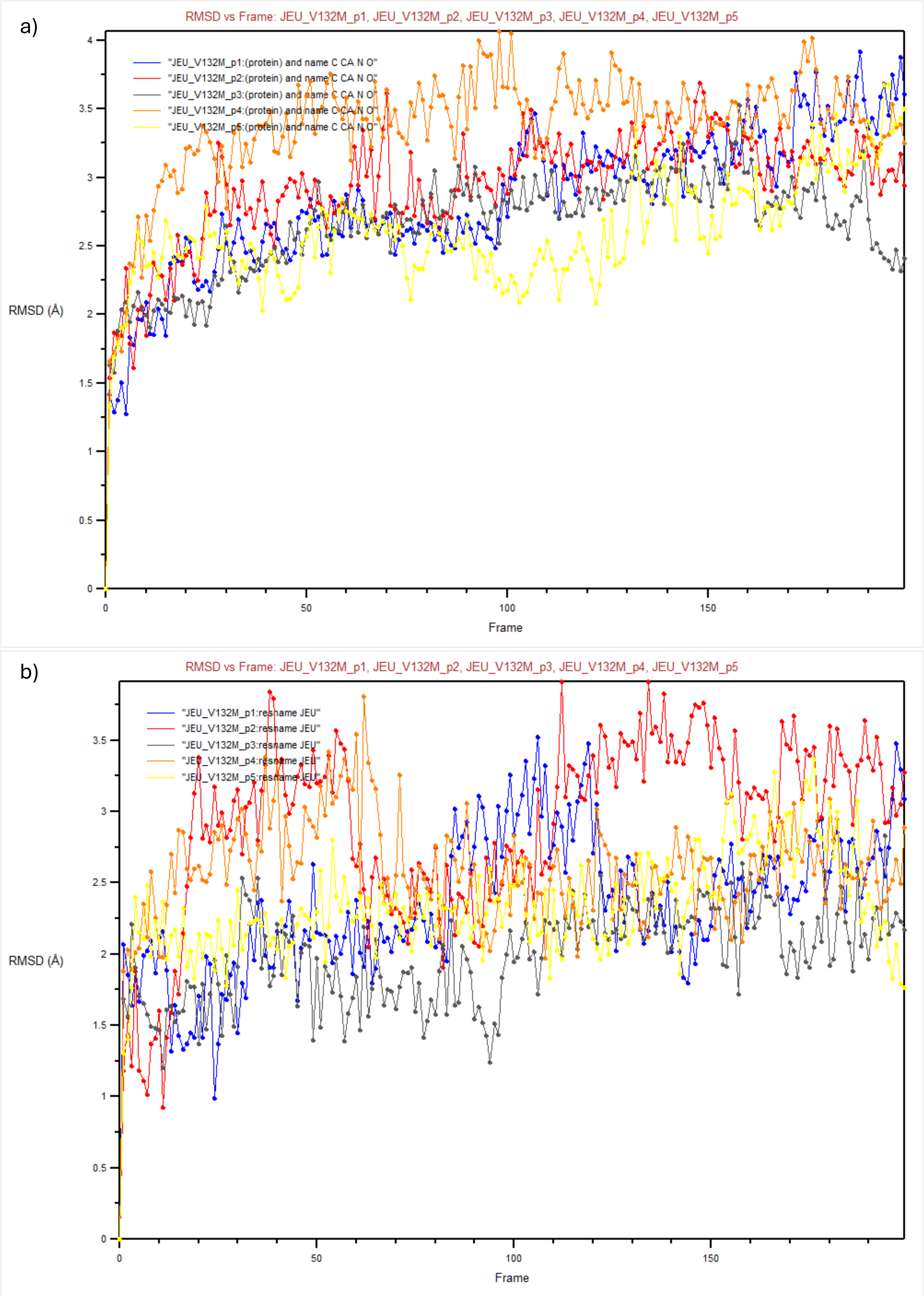

Figure S41 – RMSD of a) protein with V132$^{3.40}$M mutation and b) ozanimod in relation to the frames with five parallel simulations. A total of 200 frames coincided with 100 ns of simulation.

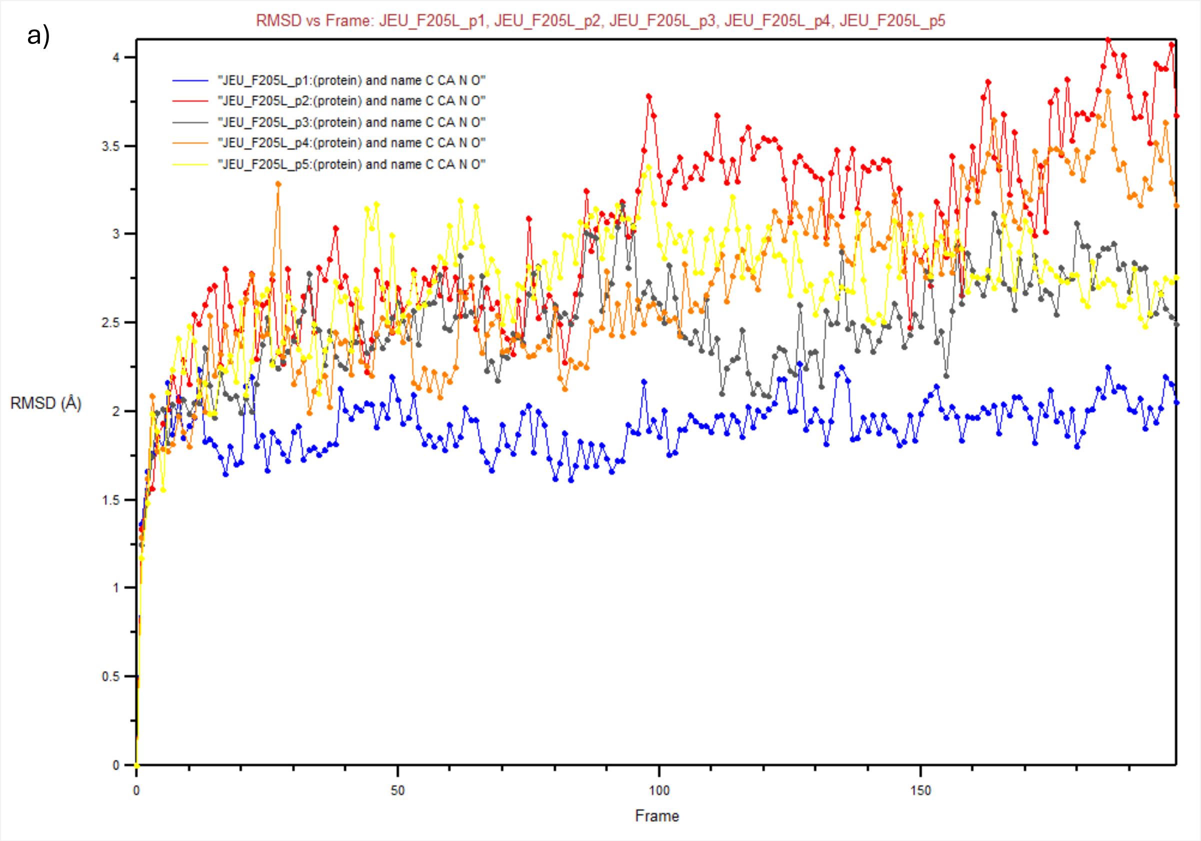

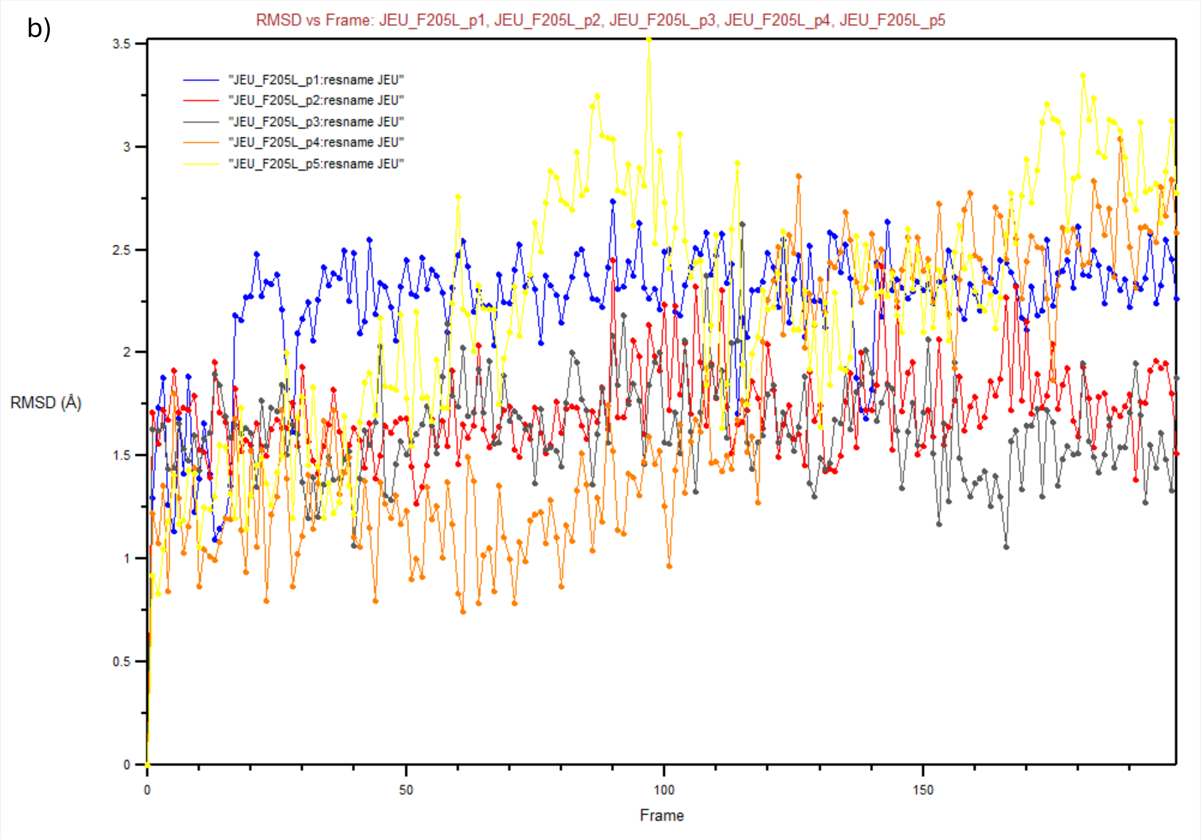

Figure S42 – RMSD of a) protein with F205$^{5.42}$L mutation and b) ozanimod in relation to the frames with five parallel simulations. A total of 200 frames coincided with 100 ns of simulation.

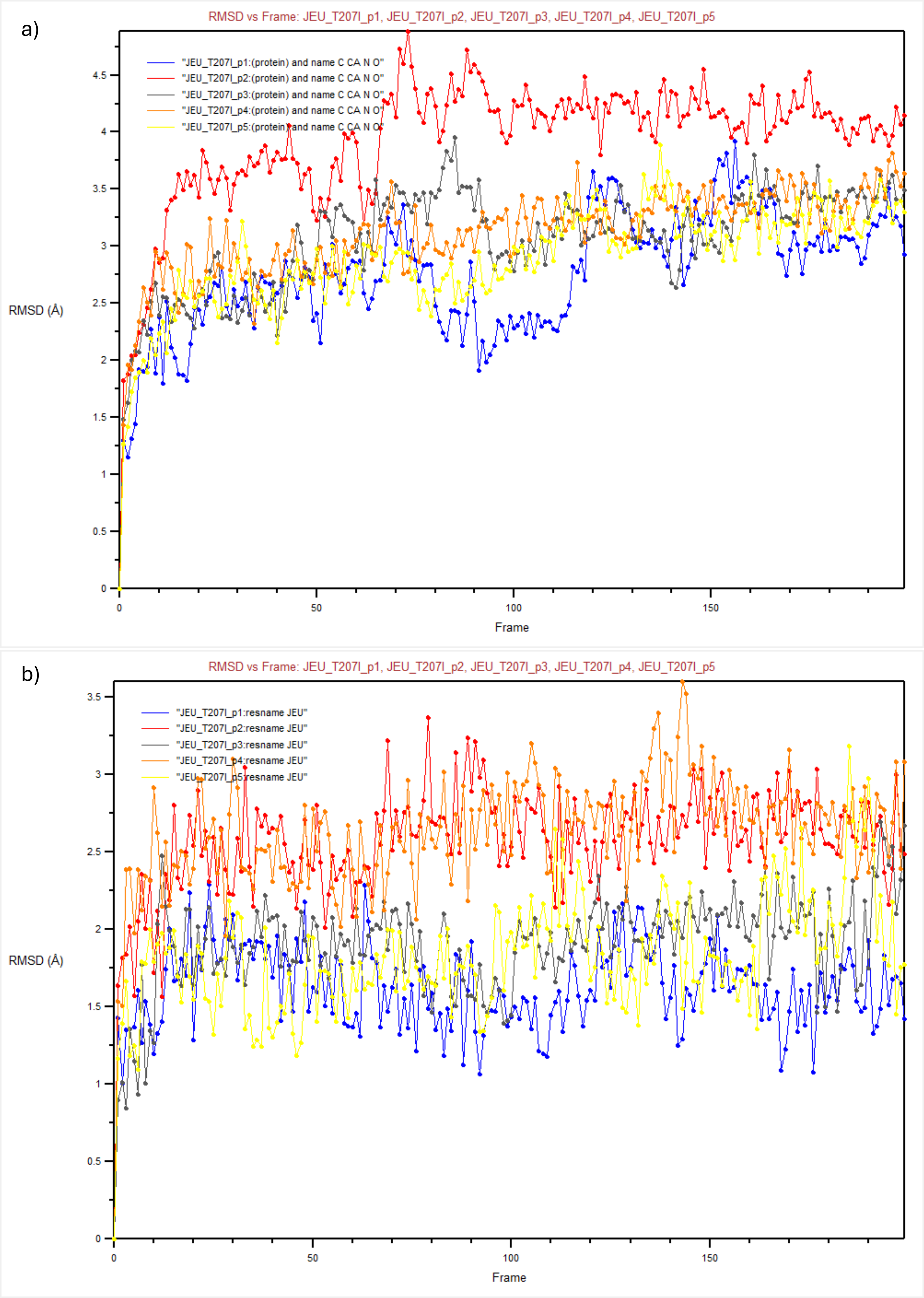

Figure S43 – RMSD of a) protein with T207$^{5.44}$I mutation and b) ozanimod in relation to the frames with five parallel simulations. A total of 200 frames coincided with 100 ns of simulation.

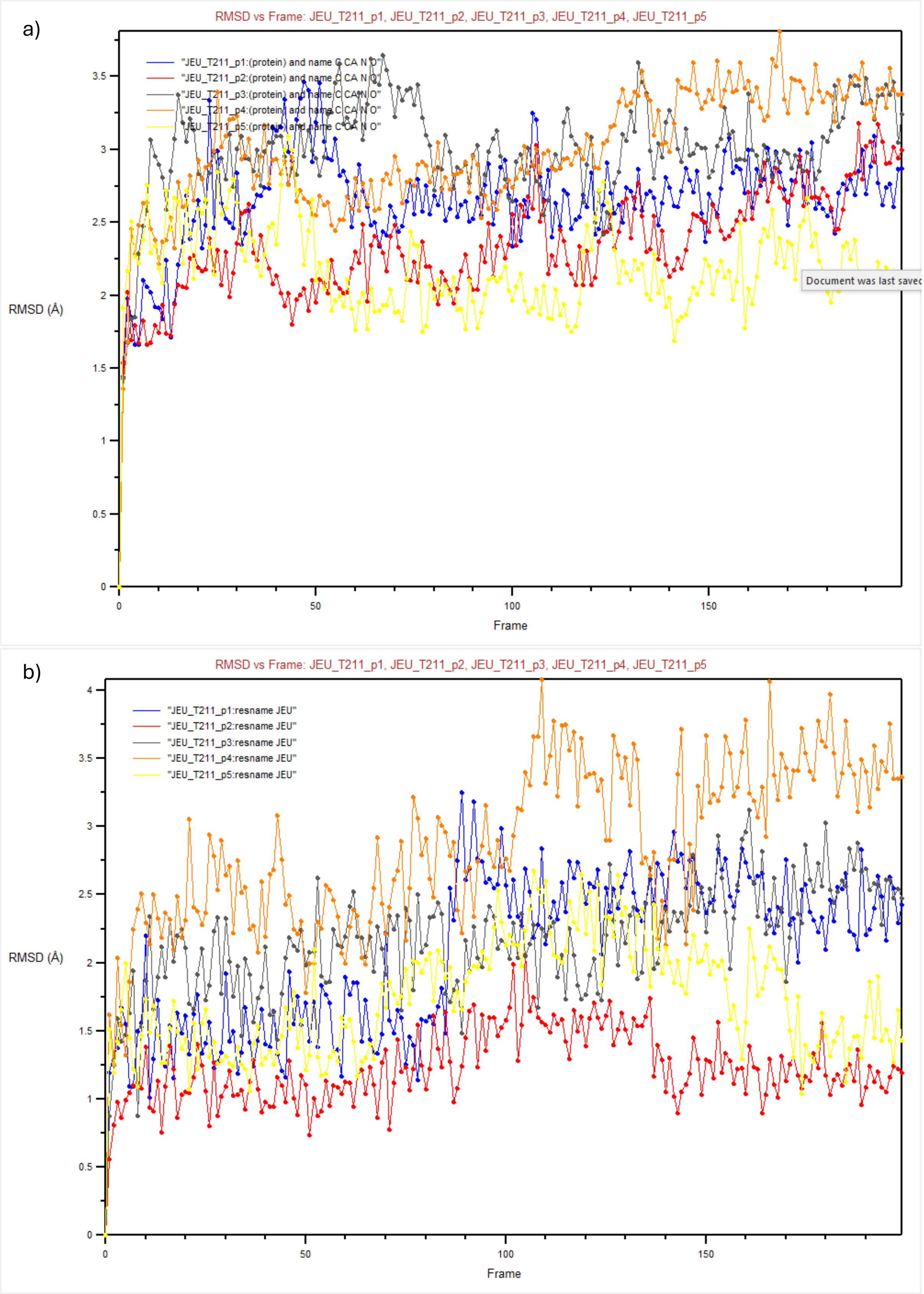

Figure S44 – RMSD of a) protein with T211$^{5.48}$P mutation and b) ozanimod in relation to the frames with five parallel simulations. A total of 200 frames coincided with 100 ns of simulation.

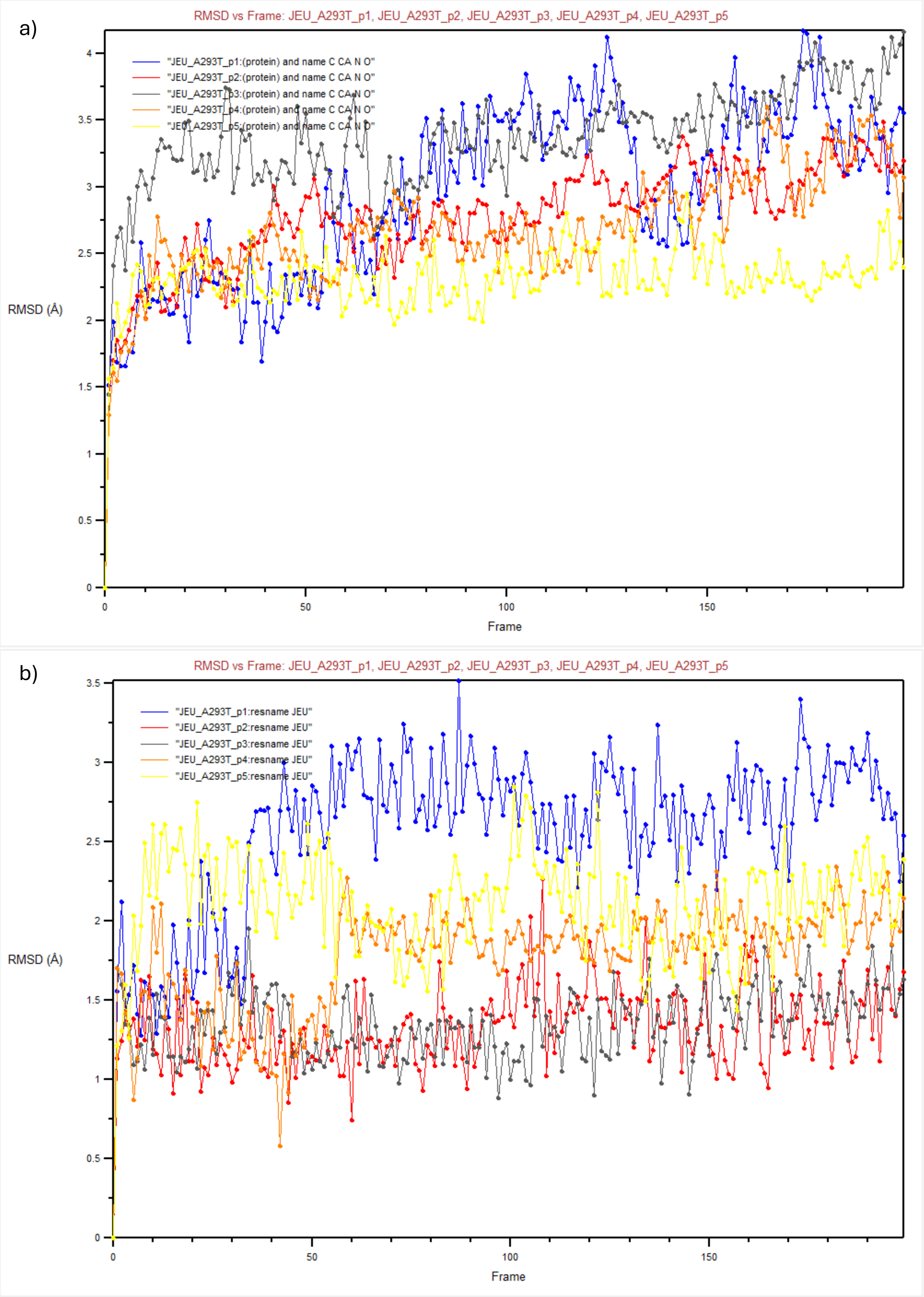

Figure S45 – RMSD of a) protein with A293$^{7.35}$T mutation and b) ozanimod in relation to the frames with five parallel simulations. A total of 200 frames coincided with 100 ns of simulation.

a)

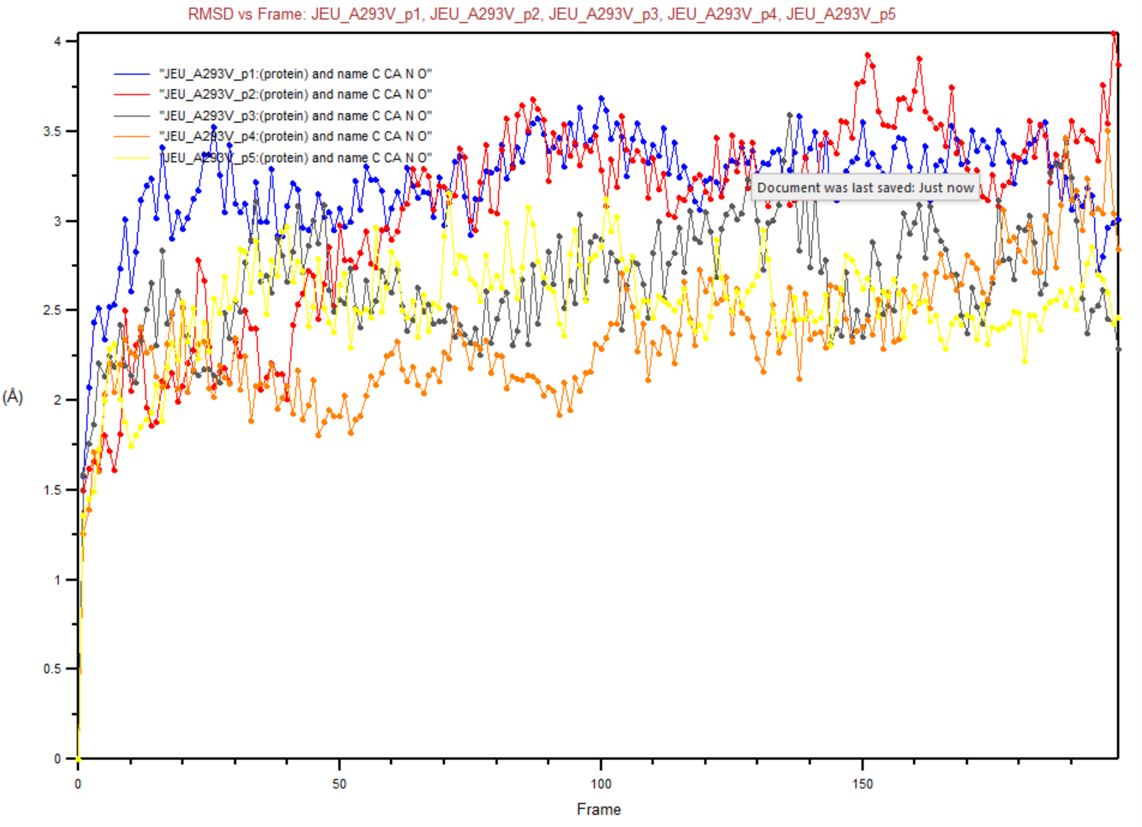

b)

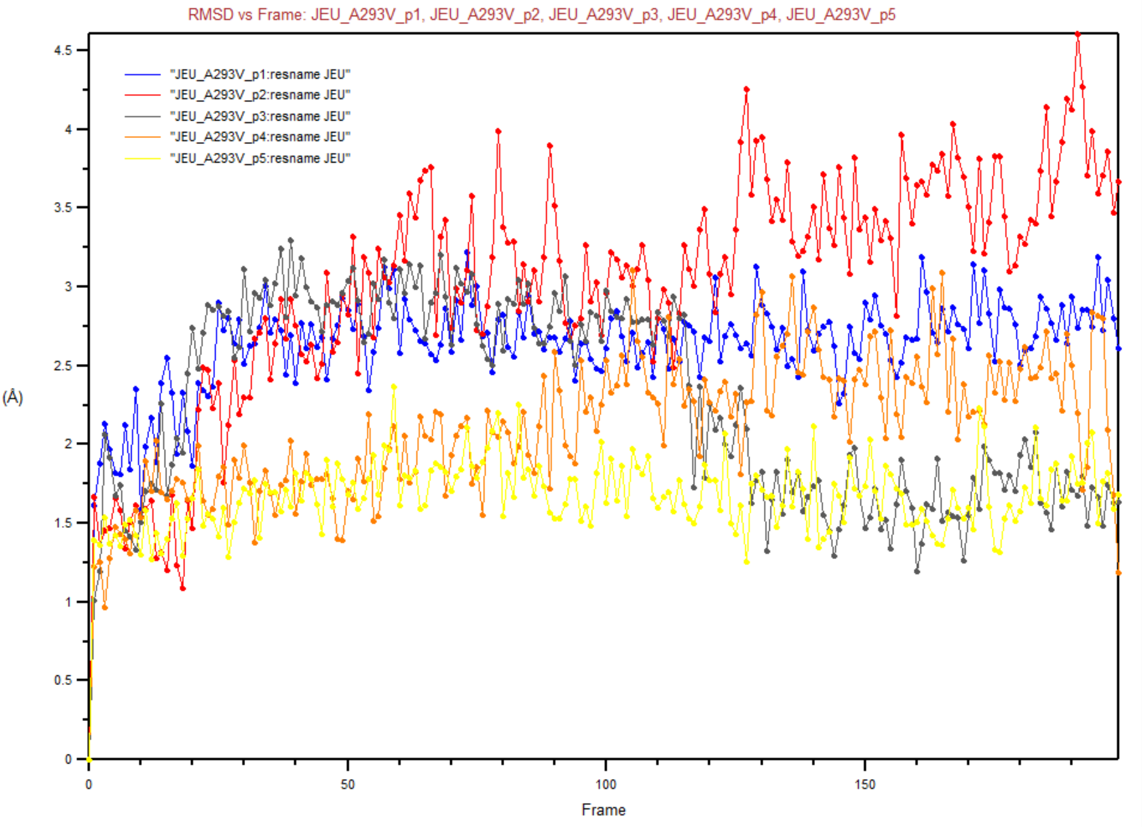

Figure S46 – RMSD of a) protein with A293$^{7.35}$V mutation and b) ozanimod in relation to the frames with five parallel simulations. A total of 200 frames coincided with 100 ns of simulation.

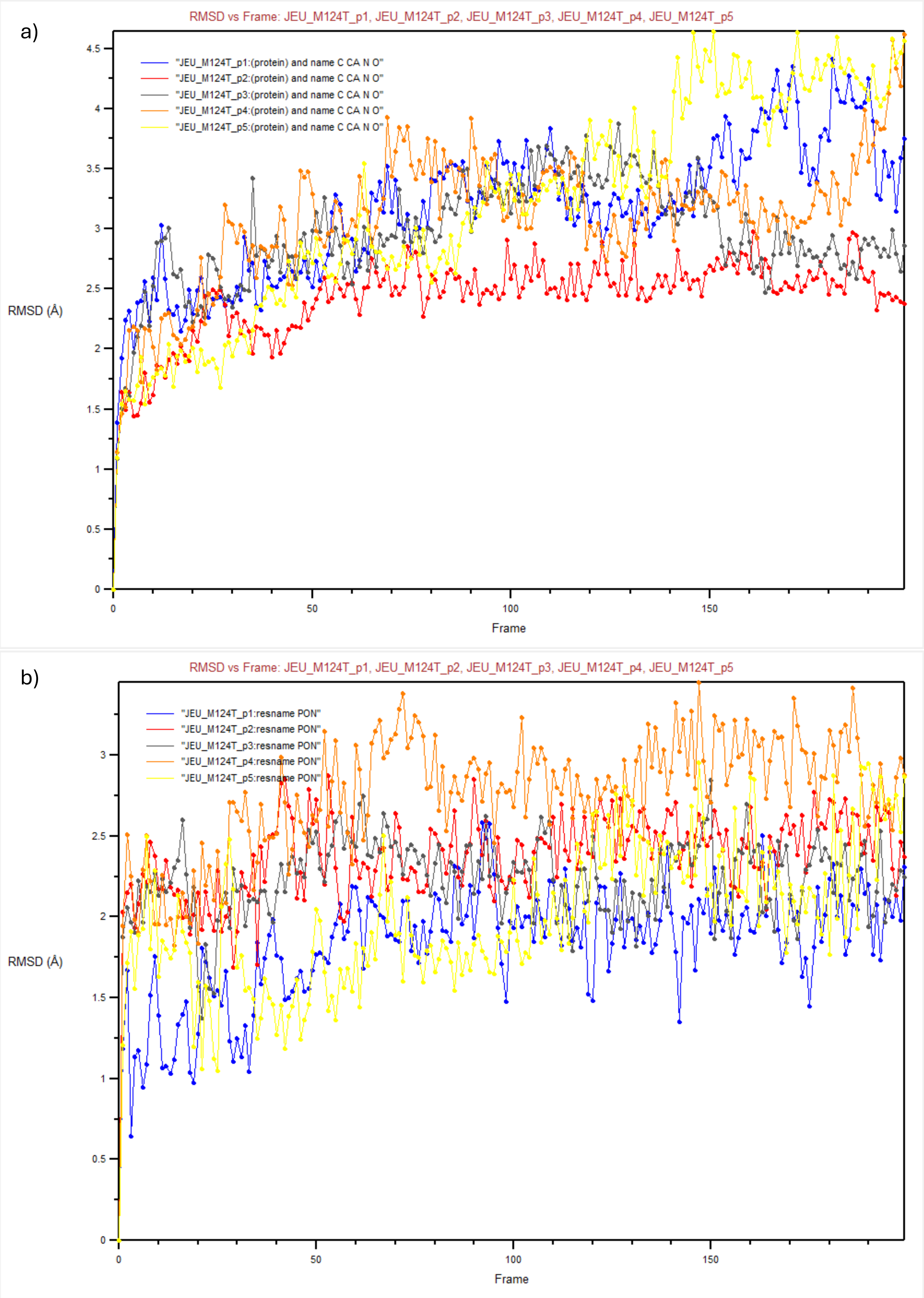

Figure S47 – RMSD of a) protein with M124$^{3.32}$T mutation and b) ponesimod in relation to the frames with five parallel simulations. A total of 200 frames coincided with 100 ns of simulation.

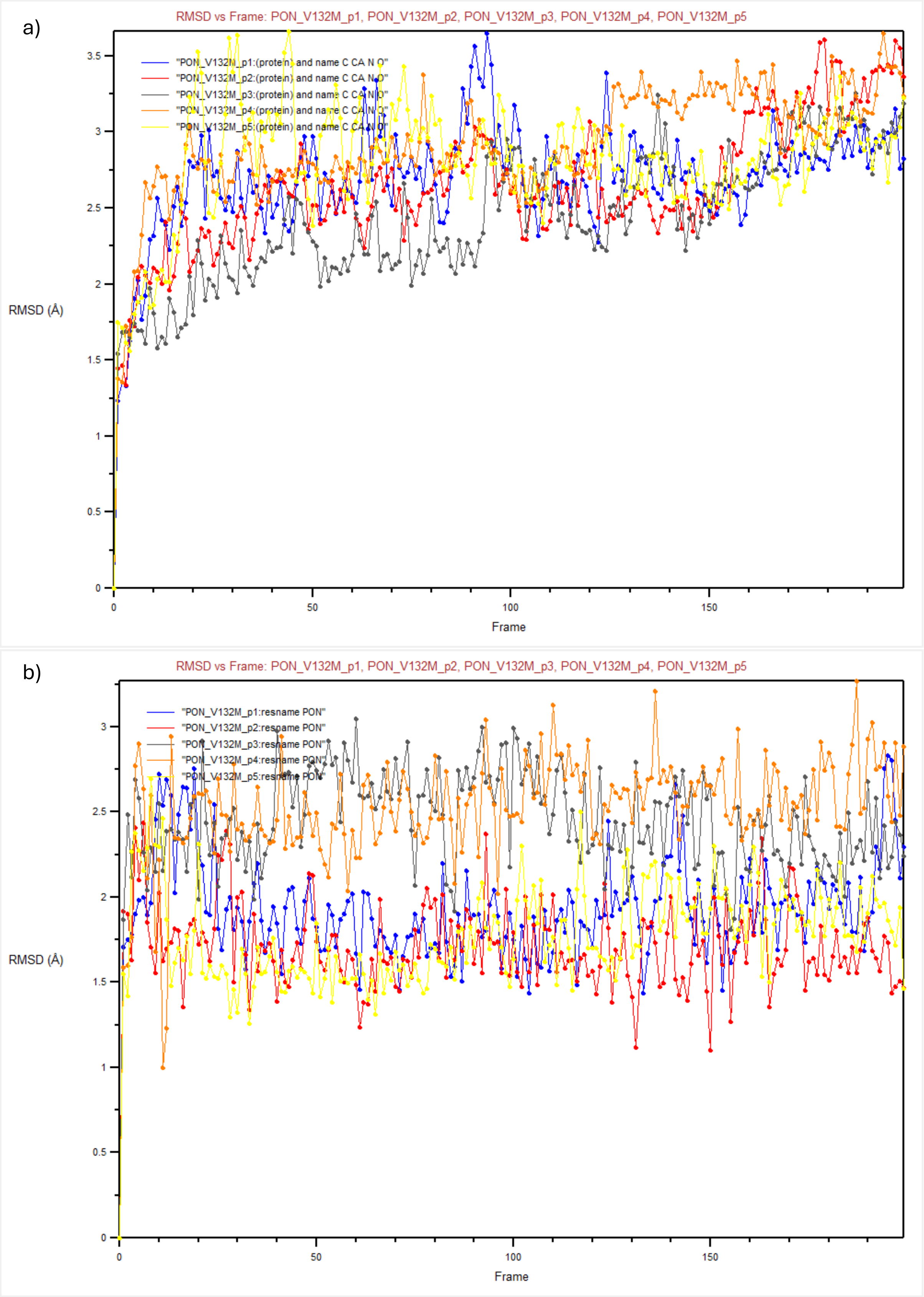

Figure S48 – RMSD of a) protein with V132$^{3.40}$M mutation and b) ponesimod in relation to the frames with five parallel simulations. A total of 200 frames coincided with 100 ns of simulation.

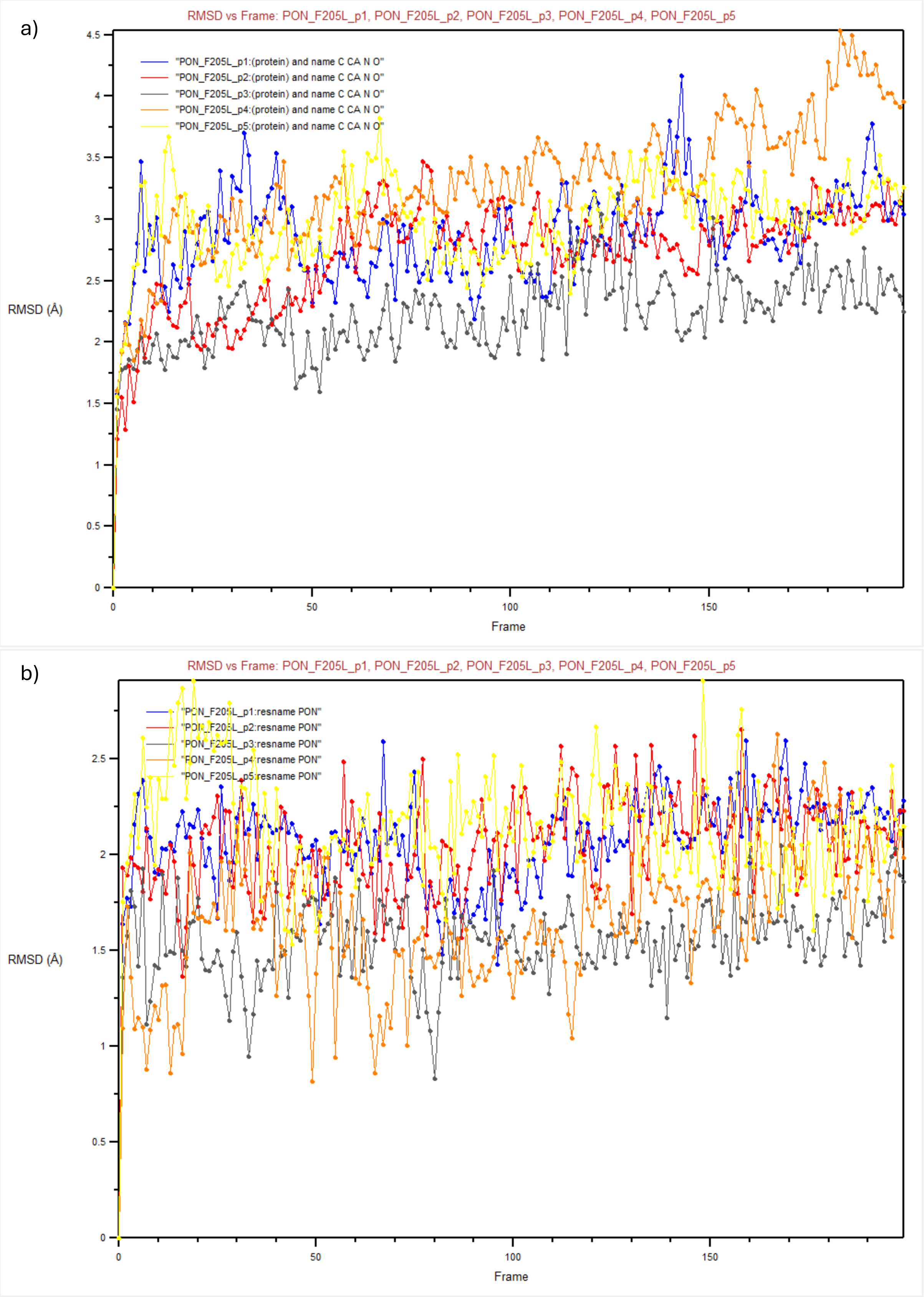

Figure S49 – RMSD of a) protein with F205$^{5.42}$L mutation and b) ponesimod in relation to the frames with five parallel simulations. A total of 200 frames coincided with 100 ns of simulation.

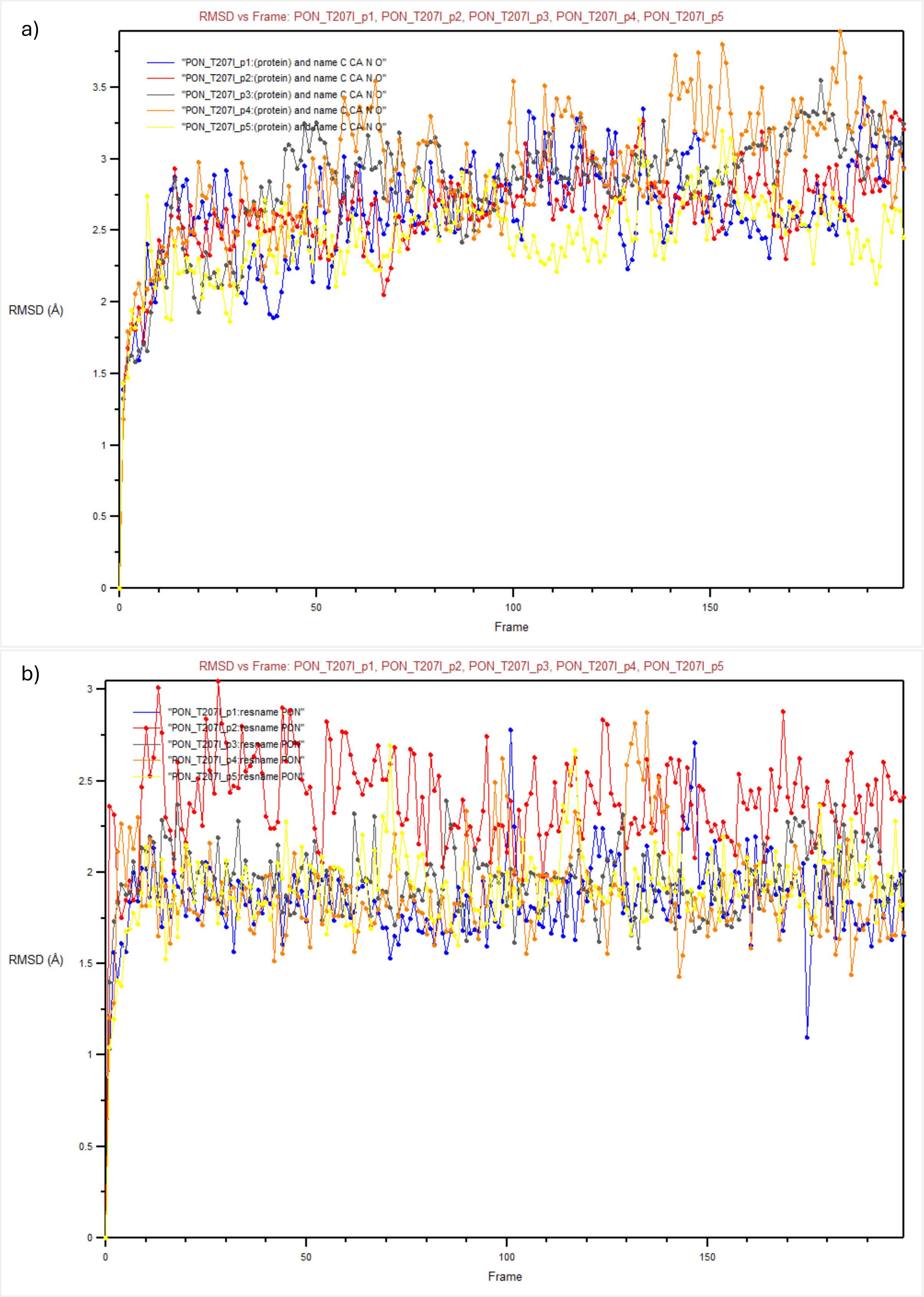

Figure S50 – RMSD of a) protein with T207$^{5.44}$I mutation and b) ponesimod in relation to the frames with five parallel simulations. A total of 200 frames coincided with 100 ns of simulation.

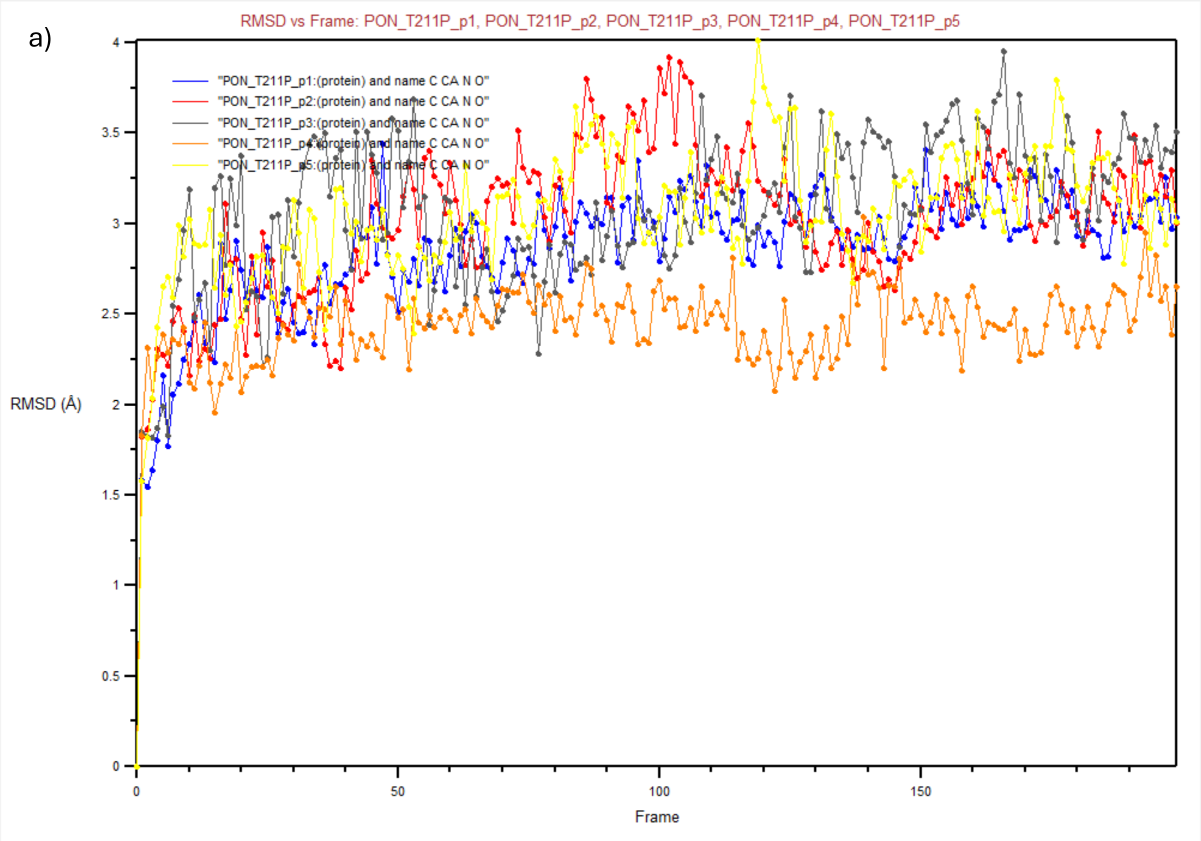

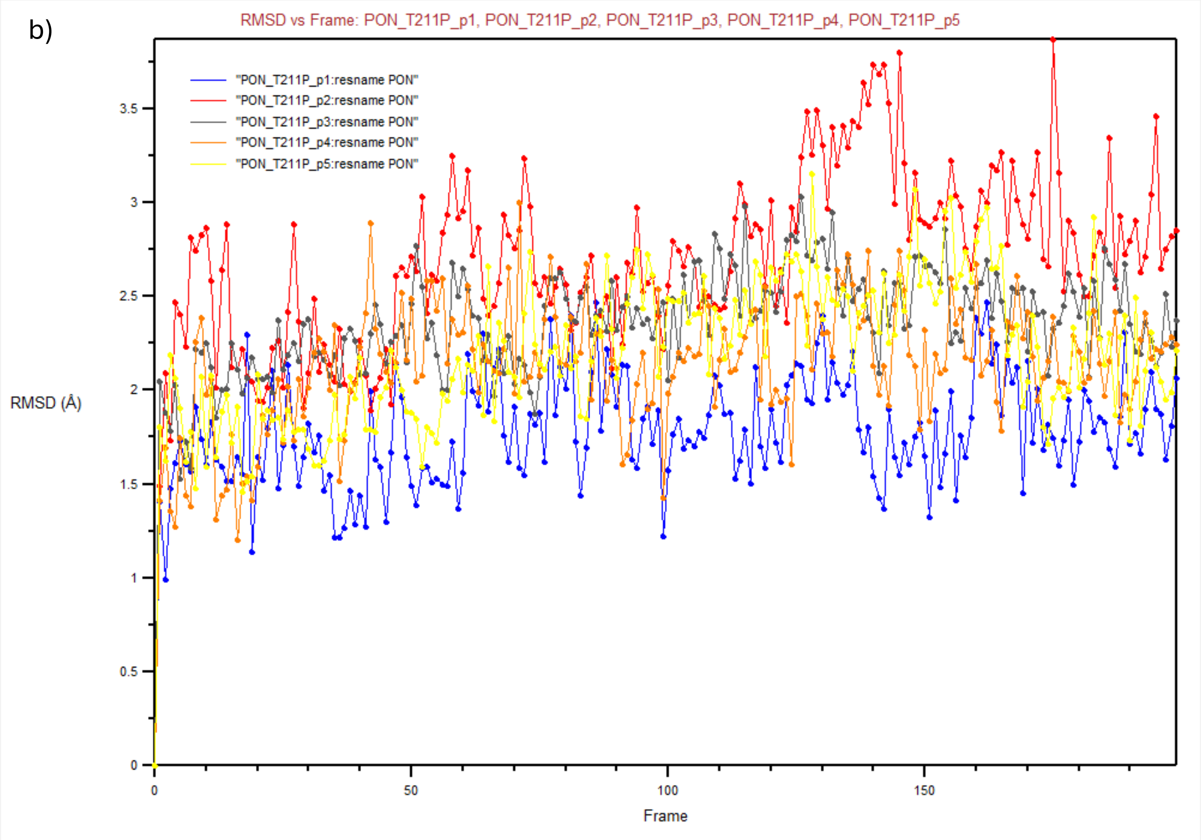

Figure S51 – RMSD of a) protein with T211$^{5.48}$P mutation and b) ponesimod in relation to the frames with five parallel simulations. A total of 200 frames coincided with 100 ns of simulation.

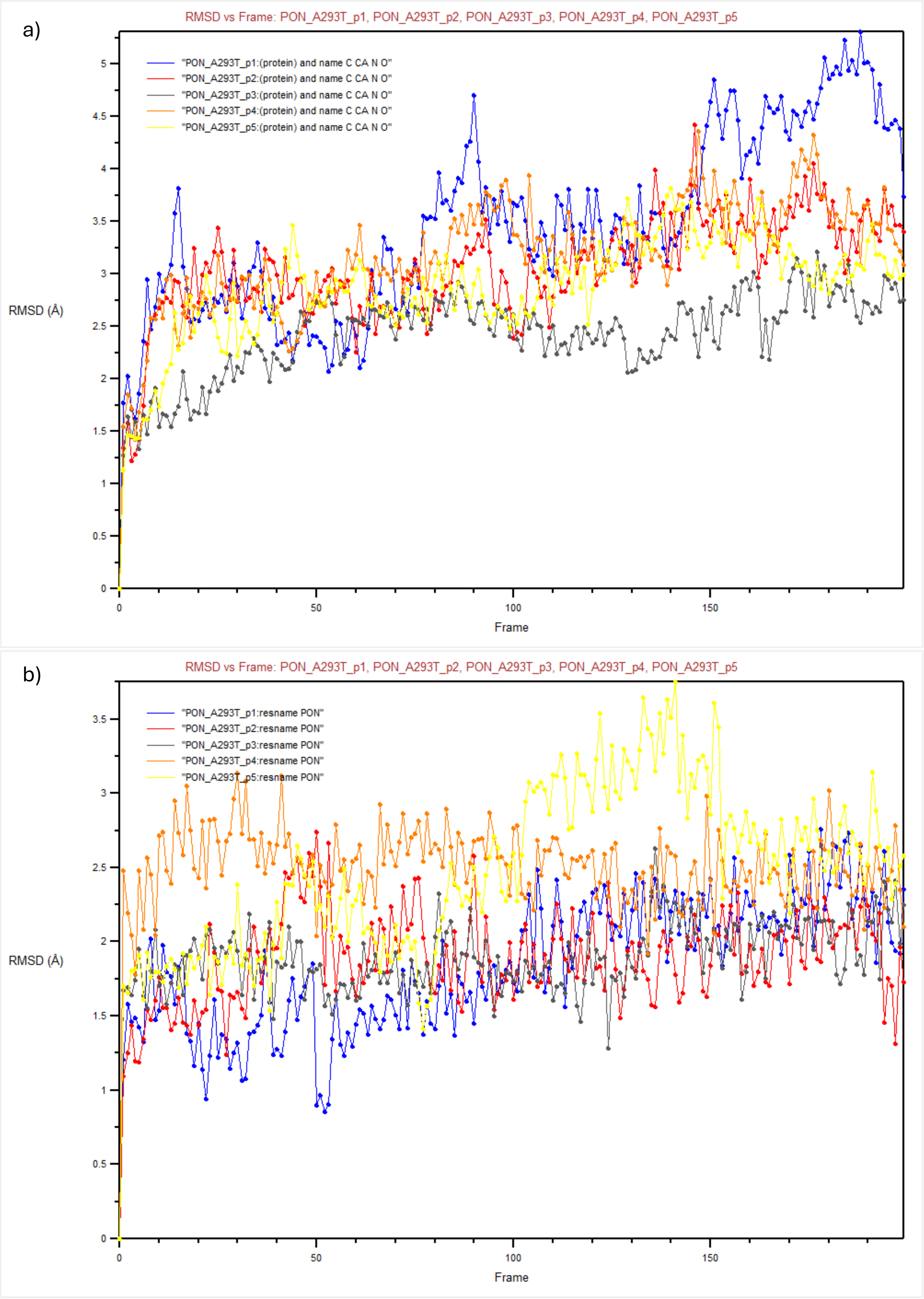

Figure S52 – RMSD of a) protein with A293$^{7.35}$T mutation and b) ponesimod in relation to the frames with five parallel simulations. A total of 200 frames coincided with 100 ns of simulation.

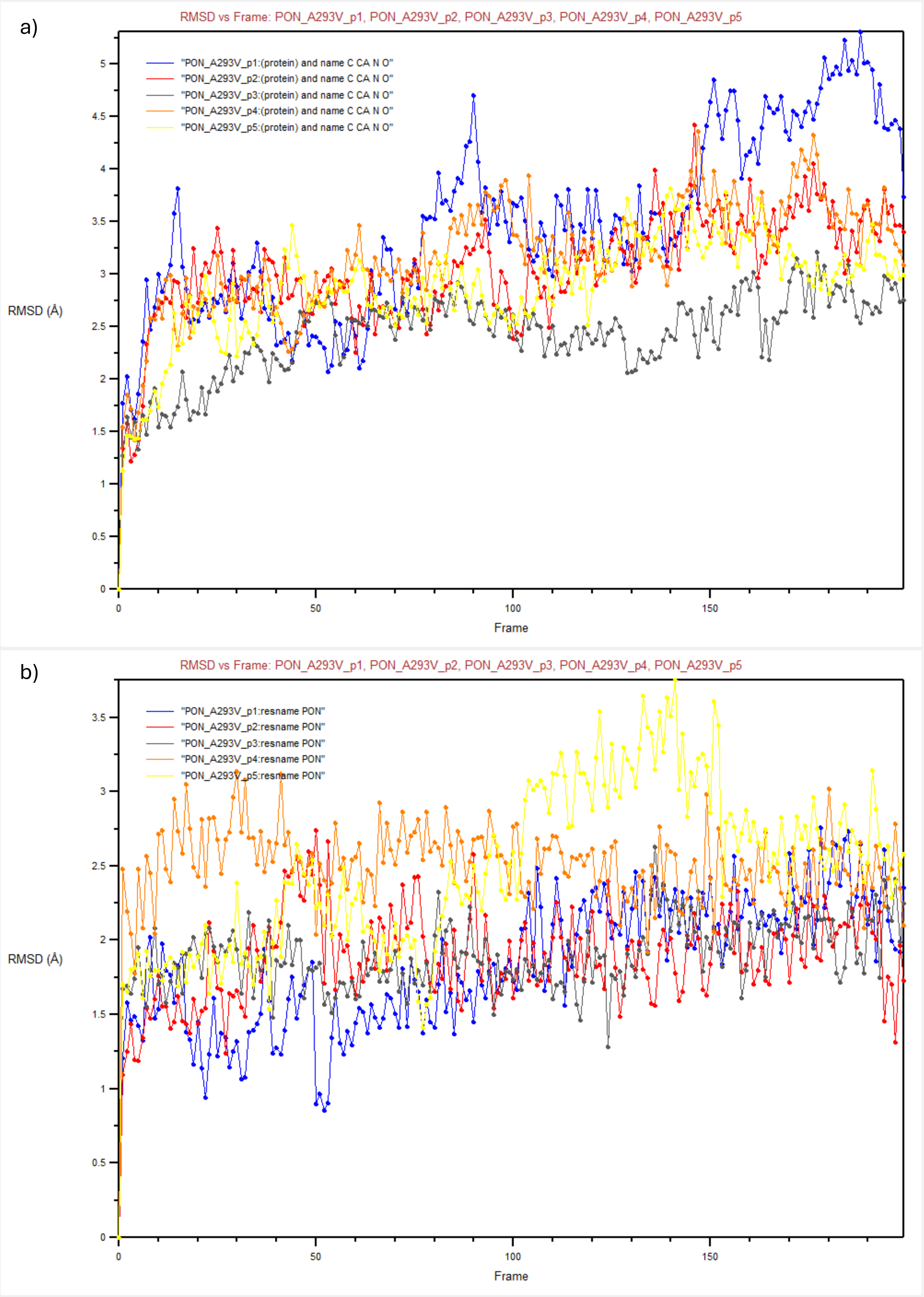

Figure S53 – RMSD of a) protein with A293$^{7.35}$V mutation and b) ponesimod in relation to the frames with five parallel simulations. A total of 200 frames coincided with 100 ns of simulation.

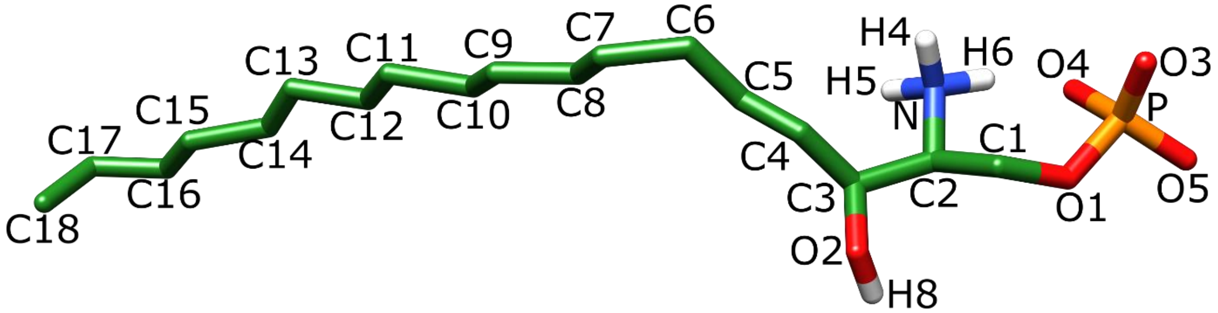

Figure S54 – S1P structure with atom numbering.

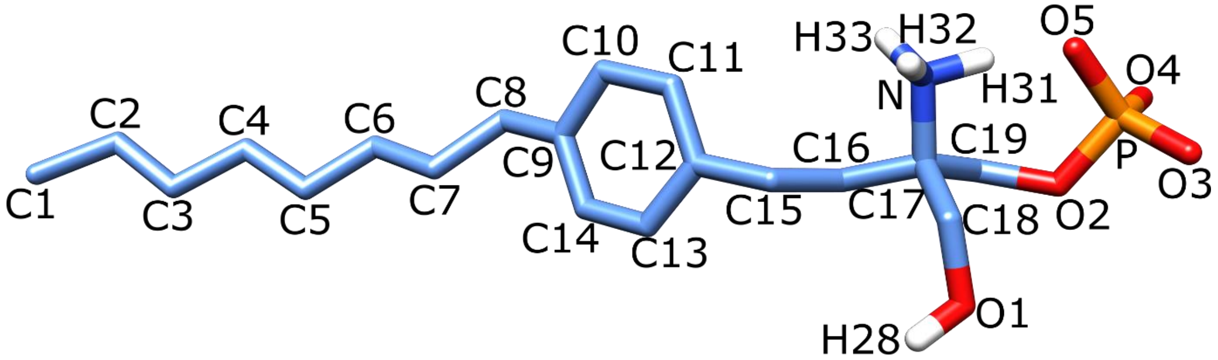

Figure S55 – Fingolimod phosphate structure with atom numbering.

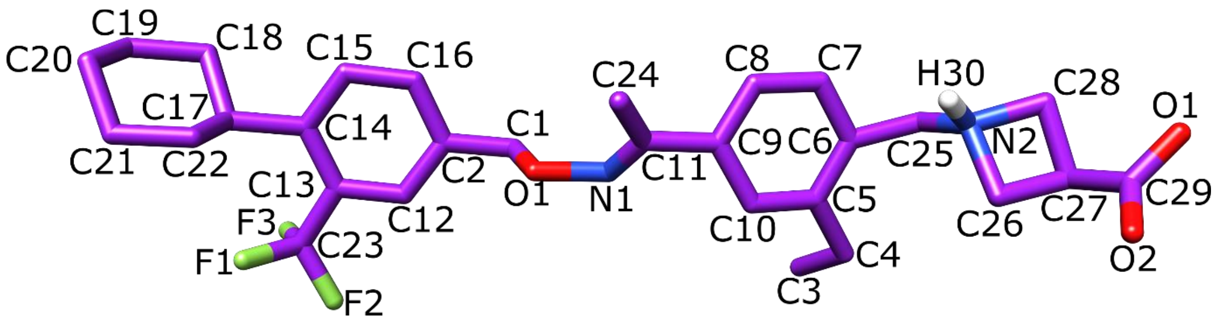

Figure S56 – Siponimod structure with atom numbering.

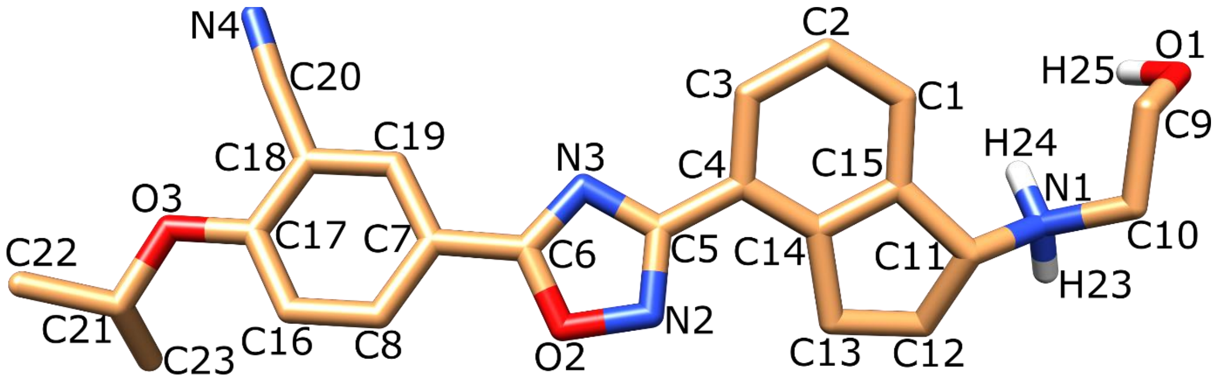

Figure S57 – Ozanimod structure with atom numbering.

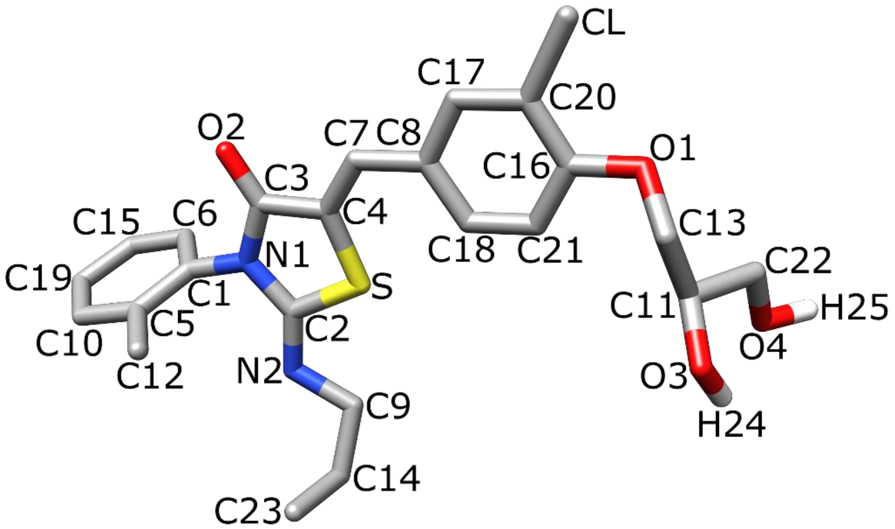

Figure S58 – Ponesimod structure with atom numbering.
